# Fluorescent Ligand-Based Discovery of Small-Molecule Sulfonamide Agonists for GPR120

**DOI:** 10.3389/fchem.2022.816014

**Published:** 2022-01-31

**Authors:** Siyue Ma, Zhenzhen Li, Yueli Yang, Ling Zhang, Minyong Li, Lupei Du

**Affiliations:** Key Laboratory of Chemical Biology (MOE), Department of Medicinal Chemistry, School of Pharmacy, Cheeloo College of Medicine, Shandong University, Jinan, China

**Keywords:** GPR120 agonist, fluorescent ligand, BRET, glucose-lowering effect, type 2 diabetes

## Abstract

As a critical member of G protein-coupled receptors (GPCRs), G protein-coupled receptor 120 (GPR120) is a potential target for many physiological diseases, such as type 2 diabetes mellitus, inflammation, and obesity. Considering that small-molecule fluorescent ligands can combine the advantages of visualization, high sensitivity and selectivity, we initially undertook an effort to develop a series of fluorescent ligands to track GPR120 and establish a method to screen GPR120 agonists. The representative fluorescent ligand N1 possesses suitable optical property, equitable biological activity, and high fluorescence imaging feasibility, therefore, based on compound N1, we subsequently founded a bioluminescence resonance energy transfer (BRET) competition binding assay to screen three series of sulfonamide GPR120 agonists we developed herein. The activity evaluation results revealed that compound D5 was a potent GPR120 agonist with high activity and selectivity. Moreover, compound D5 exhibited a significant glucose-lowering effect in db/db mice, which indicates its potential application in the treatment of type 2 diabetes mellitus *in vivo*. It is anticipated that our fluorescent ligand-based method is a useful toolbox and will find broad applications in the discovery of small-molecule agonists for GPR120.

## 1 Introduction

G protein-coupled receptors (GPCRs) are the most significant type of membrane protein family involved in signal transduction on the cell surface. (Civelli et al., 2013) They can stimulate signals outside the cell membrane into the cell through receptors on the cell membrane concerning the mechanisms of many diseases. (Ichimura et al., 2012; Hauser et al., 2017; Gurevich and Gurevich, 2019) G protein-coupled receptor 120 (GPR120) is one member of the GPCRs family, known as the *ω*3 fat receptor 1. Like other GPCRs, it contains an extracellular N-terminal domain, an intracellular C-terminal domain, (Shimpukade et al., 2012) and seven transmembrane structures. (Leung and Zhang, 2014) GPR120 is highly expressed in the small intestine of humans and mice, and abundantly in adipocytes and macrophages. (Hirasawa et al., 2005) Its expression level on tissues and cells reveals that GPR120 may be related to the presence of many diseases, such as diabetes, inflammation, and obesity. (Leung and Zhang, 2014; Hilgendorf et al., 2019; Miyamoto et al., 2019) Therefore, GPR120 can induce the secretion of GLP-1 under the induction of ω-3 fatty acids, which can inhibit eating and promote insulin synthesis. (Alvarez-Curto et al., 2016) Also, when a ligand activates GPR120, *β*-arrestin2 can be recruited to the cell membrane and coupled with GPR120, and then enter the cytoplasm through endocytosis, thereby inhibiting the binding of TAB1 and TAK1, and achieving the purpose of interfering with pro-inflammatory signaling. (Yan et al., 2013) Therefore, GPR120 has become an essential target for diabetes, inflammation, and obesity.

**SCHEME 1:**
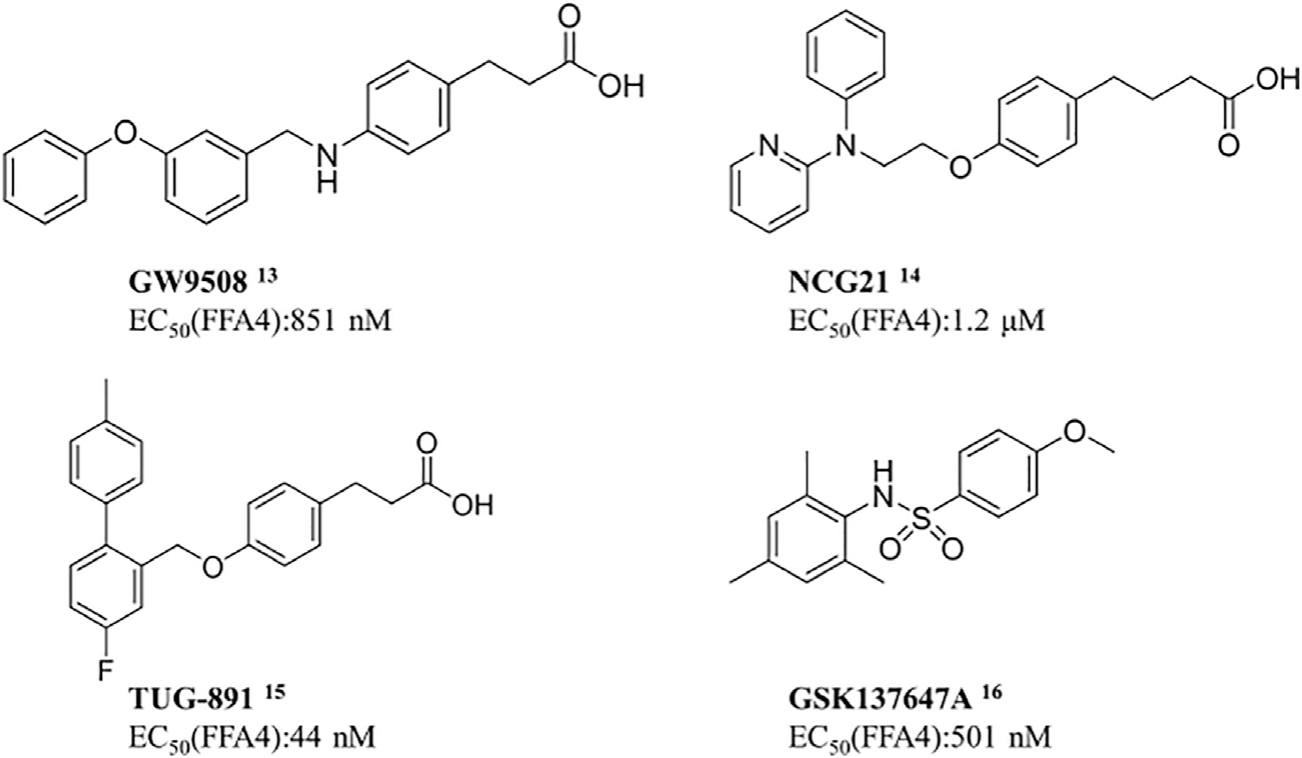
Current agonists of GPR120.

Scientists have discovered and researched many natural and synthetic agonists targeting GPR120. (Milligan et al., 2017) GW9508 is the first GPR120 small molecule agonist determined in recent years. (Garrido et al., 2006) In 2010, Suzuki et al. found that NCG21 could promote the secretion of GLP-1 in STC-1. (Sun et al., 2010) In 2012, a small molecule TUG-891 targeting GPR40 was found to have high selectivity to GPR120 as well. (Azevedo et al., 2016) All these three are aromatic fatty carboxyl compounds. In 2014, Steven et al. reported the first small sulfonamide compound, GSK137647A, with high agonistic activity with GPR120. (Sparks et al., 2014) *In vitro* studies exhibited that the molecule discloses the best selectivity so far (Scheme 1). (Hansen and Ulven, 2016)

With the deepening of the research on GPR120, it is necessary to develop a GPR120 fluorescent ligand to monitor the receptor-ligand interaction in a real-time manner (Ma et al., 2014) to establish a method to screen more potent GPR120 agonists. (Iliopoulos-Tsoutsouvas et al., 2018) Small-molecule fluorescent tracers are divided into three parts: recognition groups, linker chains, and fluorophores. (Ma et al., 2016) We have successfully designed and synthesized a series of aromatic acid-based fluorescent ligands on the scaffold of TUG-1197 previously. (Liu et al., 2019) Because GSK137647A has high selectivity, we chose it as a recognition group to develop another series of small-molecule fluorescent ligands herein. Because the ligand-binding region of GPR120 belongs to a high-viscosity and low-polarity environment, we chose the environmentally sensitive fluorophores naphthalenediimide (Klymchenko, 2017; Liu et al., 2017) to make the probe emit weak fluorescence in an aqueous solution, while in the low-polarity environment emits strong fluorescence.

Bioluminescence resonance energy transfer (BRET) is a mature method for detecting the interactions between protein-protein and has been applied to GPCRs signal transduction and drug screening. (Stoddart et al., 2015) When a bioluminescence donor and a fluorescent acceptor are close enough to interact, energy transfer will occur. In the BRET experiments, we chose Renillaluciferase (Rluc) as a bioluminescence donor and naphthalimide as an acceptor because the excitation spectrum of naphthalimide fluorophore and the emission spectrum of the Rluc receptor tag is close to the ideal overlap. We established a BRET competition binding assay with the most potent probe to screen other ligands on GPR120. Agonists with good preliminary BRET screening results are used for subsequent calcium ion (Ca^2+^) assay, bias test, (Rajagopal et al., 2011) and blood glucose experiment in mice. (McCoull et al., 2017)

## 2 Results and Discussion

### 2.1 The Design of Fluorescent Ligands (N1-N3)

**SCHEME 2:**
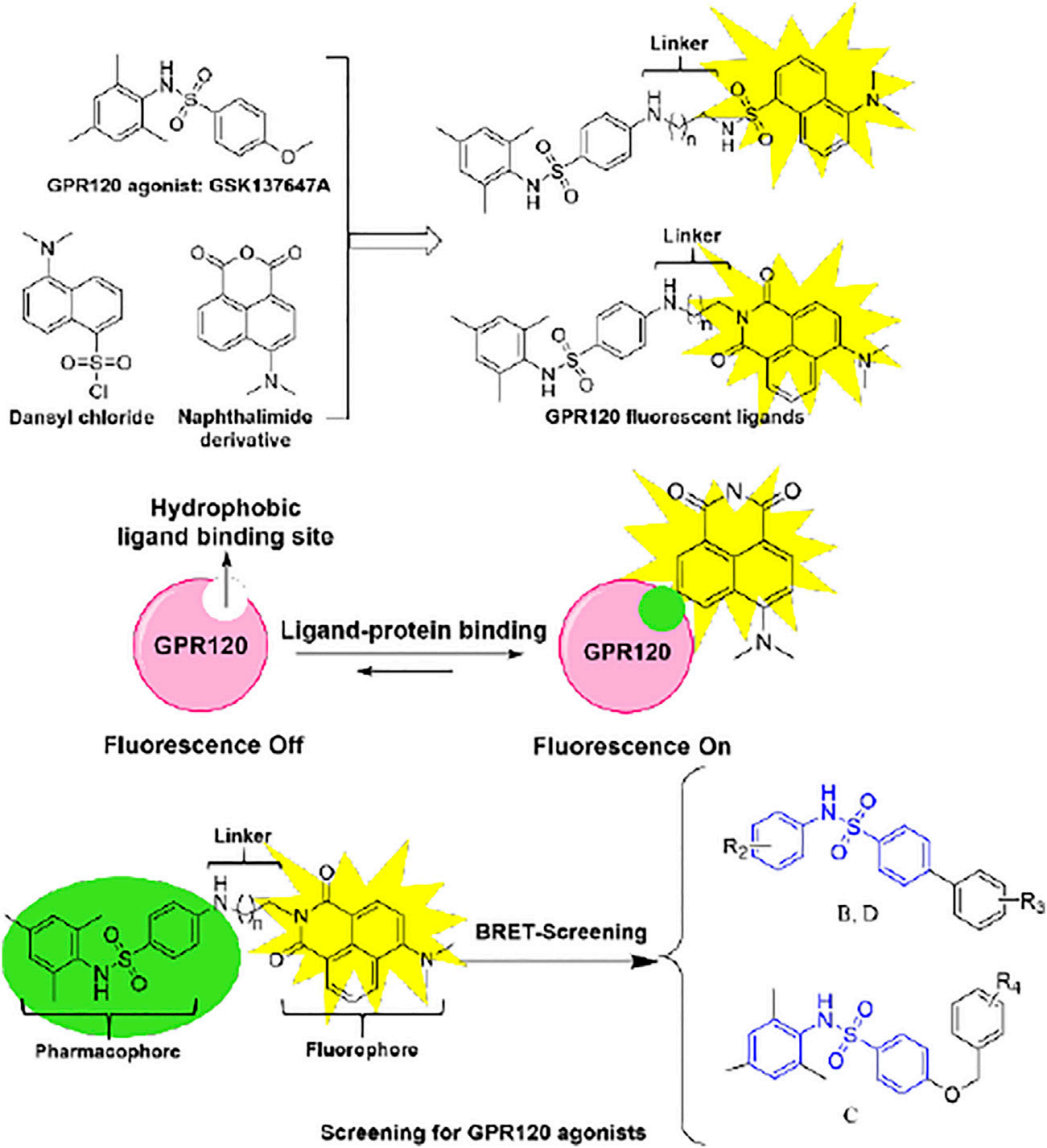
Design flow and mechanism of GPR120 fluorescent ligands.

GPR120 ligands with fluorescent properties can monitor the interaction between ligand and GPR120 in real-time. Because the ligand-binding area is of high viscosity and low polarity, we need to develop environment-sensitive fluorescent ligands. GSK137647A has good pharmacological activity and selectivity to GPR120 than G protein-coupled receptor 40 (GPR40), so we selected it as the pharmacophore. For the fluorophore, we selected the naphthalimide, which is environment-sensitive. Considering that the length of linkers will also affect fluorescent ligands’ activity, we designed different lengths of linkers (Scheme 2).

### 2.2 Synthesis of Fluorescent Ligands (N1-N3)

**SCHEME 3:**
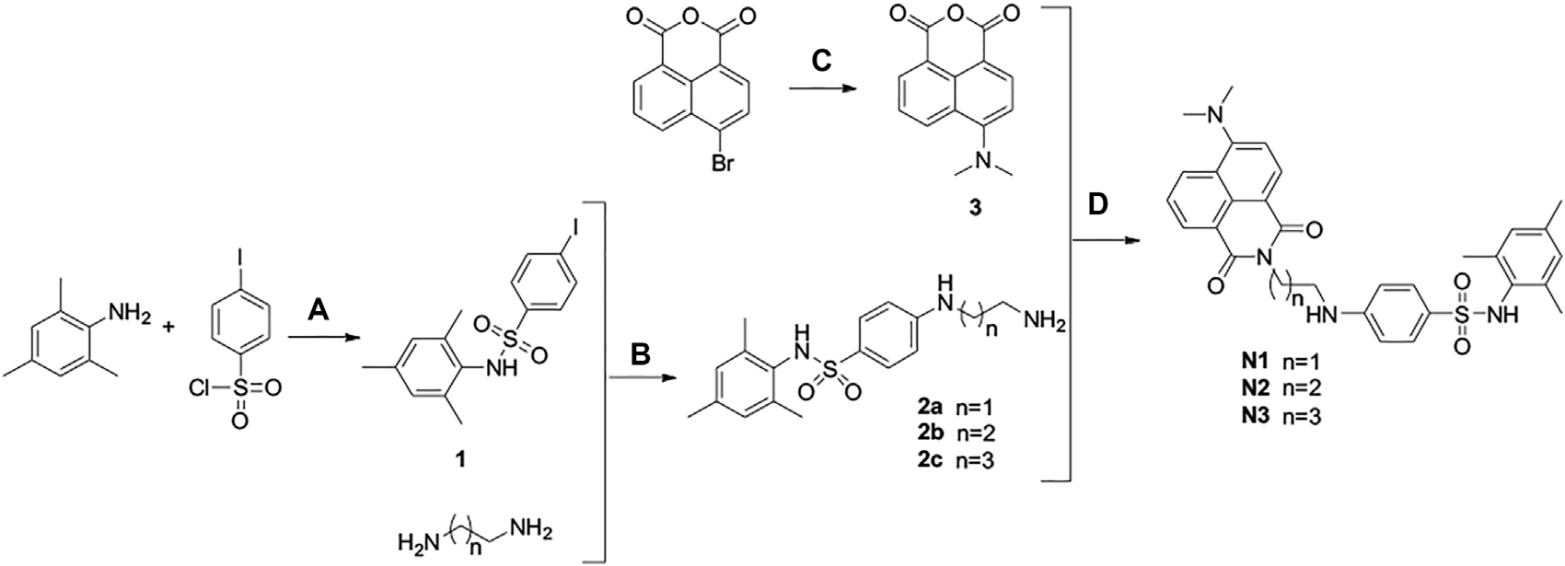
Synthesis of ligands N1-N3. Reaction conditions and reagents: (a) pyridine, dichloromethane, 6°h; (b) CuI, N_2_, 100°C, 16°h; (c) DCM, CuSO_4_·5H_2_O, DMF, 150°C, 12°h; (d) absolute ethanol, 80°C, 7 h.

The synthetic route of fluorescent ligands N1-N3 is depicted in Scheme 3, in which intermediate 1 is afforded by sulfonamide reaction between 2,4,6-trimethylaniline and *p*-iodobenzenesulfonyl chloride. After a two-step substitution reaction, we obtained the fluorescent ligands N1-N3.

### 2.3 The Spectroscopic and Pharmacologic Properties of Fluorescent Ligands

The excitation wavelength, emission wavelength, fluorescence quantum, and ultraviolet maximum absorption wavelength of fluorescent ligands are depicted in Table 1 and Supplementary Figure S7. The stokes shift values are all higher than 60 nm (Supplementary Figures S1–S3) in PBS buffer (pH = 7.4). The results display that the fluorescence intensity surges with increasing concentration. Besides, the fluorescence spectra of fluorescent ligands were determined in four different polar solvents (H_2_O, DMSO, EA, MeCN). The results indicate that when the probe is in a high-polar solvent (H_2_O), the fluorescence is quenched, and the fluorescence intensity is weak. When the probe is in a low polar solvent (MeCN), its fluorescence intensity increases (Supplementary Figures S4–S6). Consequently, these fluorescent ligands can be used as environmentally sensitive small-molecule fluorescent ligands. The cell-based experimental results indicate that the intensity of probe N1 is much greater than that of N2 and N3. With the increasing carbon chain length, the brightness exhibited a significant weakening trend. The reason of this phenomenon is that the increasing chain length tends to extend to the hydrophilic solvent area, which quenches the fluorescence and causes the fluorescence to weaken. The brightness of HEK293 cells was significantly greater than that of PC-3 cells at the same concentration (Supplementary Figures S8–S10).

**TABLE 1:** Spectroscopic properties and pharmacological properties of our fluorescent probes.

Probe	pEC_50_ ^a^		MeCN	PBS^b^			
	Ca^2+^ assay	BRET	λ_abs_ (nm)	λ_ex_ (nm)	λ_em_ (nm)	SS^c^ (nm)	Φ^d^ (%)
N1	6.05 ± 0.07	6.37 ± 0.56	419	444	562	118	3.73
N2	5.20 ± 0.03	6.25 ± 0.06	418	439	562	123	2.28
N3	5.05 ± 0.35	5.75 ± 0.35	417	438	560	122	1.03
GSK137	7.35 ± 0.59	6.75 ± 0.48					
647A							

a pEC_50_ is a biological activity value obtained from the Ca^2+^ flow and the *β*-arrestin-2 activity test.

b PBS: main ingredients: Na_2_HPO_4_, KH_2_PO_4_, NaCl, and KCl.

c SS: stokes shift.

d Φ: absolute quantum yield.

The reported agonist GSK134647A was selected as the positive control in both the GPR120 Ca^2+^ assay and the BRET activity assay. The results of the Ca^2+^ flow assay (pEC_50_: 7.35 ± 0.59) and the BRET activity tests (pEC_50_: 6.75 ± 0.48) were within acceptable ranges, and as a result, these two methods were used to evaluate the probes we developed.

The experimental results presented that the short linker in fluorescent ligand may contribute to bioactivity (Table 1), in which probe N1 has high activity up to the positive control GSK134647A. In the GPR40 Ca^2+^ activity test, the reported agonist TAK875 was selected as a control. (Li et al., 2017) In brief, the Ca^2+^ response to GPR40 of probe N1 was much lower than that of TAK875, which proved that ligand N1 had a particular selectivity for GPR120 (Supplementary Figure S13).

### 2.4 Bioluminescence Resonance Energy Transfer Experiments With Fluorescent Ligands

Rluc was selected as a bioluminescence donor in the BRET experiments because the excitation spectrum of naphthalimide probes and the Rluc’s emission spectrum are close to the ideal overlap. We selected the most potent probe **N1** to conduct BRET experiments (Jenkins et al., 2010; Hansen et al., 2017)

#### 2.4.1 Bioluminescence Resonance Energy Transfer Saturation Experiment of Probe N1

**FIGURE 1:**
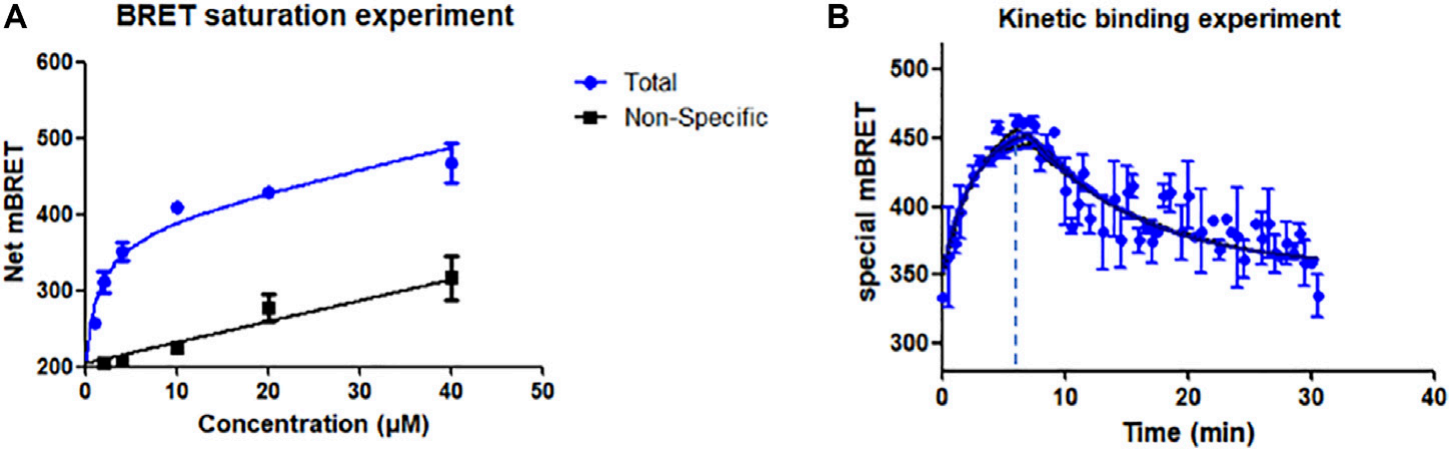
**(A)** Saturation experiment of the affinity of probe N1 at GPR120. 15 μM GSK134647A co-incubation for non-specific experiments; **(B)** Kinetic binding experiments of N1; 6 min after the addition of fluorescent probe (500 nM), GSK137647A (10 μM) was added to make the probe dissociate from GPR120.

The specific and non-specific curves of the compound show that as the concentration increases, the compound binds non-specifically to the target and affects the experimental results (Figure 1A). The K_d_ value of N1 was calculated to be 1.12 ± 0.6 μM. When the concentration of N1 is less than 2.76 μM, the non-specific binding of N1 to the target can be ignored. In the BRET competition experiment, the maximum concentration of probe N1 did not exceed 1000 nM, so it proved that the probe’s non-specific binding did not affect the results of the BRET competition experiment.

#### 2.4.2 Bioluminescence Resonance Energy Transfer Kinetic Experiment of Probe N1

Kinetic experiments indicate the rate of binding and dissociation of N1 to GPR120. The experimental results showed that the binding of N1 to GPR120 reached saturation within 6 min. After adding excess positive drug GSK137647A, compound N1 gradually dissociated from GPR120 and reached a stable state at 30 min. The kinetic binding experiment establishes that off-rate k_off_ = 0.068 min^−1^, on-rate k_on_ = 249000 min^−1^ M^−1^, and K_d_ = 273 nM, respectively (Figure 1B).

The difference of K_d_ values obtained by the BRET saturation experiment and the kinetic experiment is less than 10 times, which can be mutually verified. The experimental results prove the affinity of the fluorescent ligand N1 for GPR120.

#### 2.4.3 Bioluminescence Resonance Energy Transfer Competitive Experiment of Probe N1 (Preliminary Screening Method)

**FIGURE 2:**
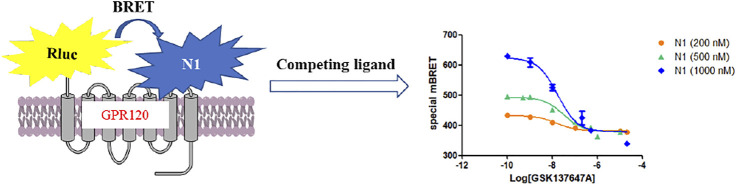
The mechanism of BRET competitive experiment.

Different concentrations of GPR120 ligands can compete with N1 at the target site. As the BRET value decreases, the competition curve can be obtained to evaluate the activity of ligands (Figure 2).

The activities of the GPR120 agonists (GSK134647A, TUG-891) and antagonist (AH-7614) measured by BRET competitive binding experiments were similar to those obtained by Ca^2+^ assay, and the theoretical values reported (Table 2, Supplementary Figure S11). (Moniri, 2016) In BRET competitive experiments of probes with different concentrations (Figure 2, Supplementary Figure S12), when probe N1 had a greater concentration, the BRET value obtained was higher, consistent with the principle of BRET activity determination.

**TABLE 2:** Comparison of BRET results to reference data.

Compd	pK_i_ ^a^ (N1)	pEC_50_ ^b^ (Ca^2+^ assay)	pEC_50_ ^c^ (Literature)
GSK137647A	6.96 ± 0.32	7.35 ± 0.59	6.30 ± 0.2^16^
TUG-891	7.06 ± 0.35	7.42 ± 0.15	7.02 ± 0.09^15^
AH-7614	<4.5	<4.5	<4.5^16^

^a^pK_i_ is the result of BRET competitive binding experiments. Probe N1 is 400 nM.

^b^pEC_50_ (Ca^2+^ assay) is the result of the Ca^2+^ assay.

c pEC_50_ is the theoretical value reported.

BRET competition experiments display that probe N1 can construct a GPR120 agonist activity screening method at 400 nM and will be applied to screen the modified agonists.

### 2.5 The Design Strategy of Modified Compounds

**FIGURE 3:**
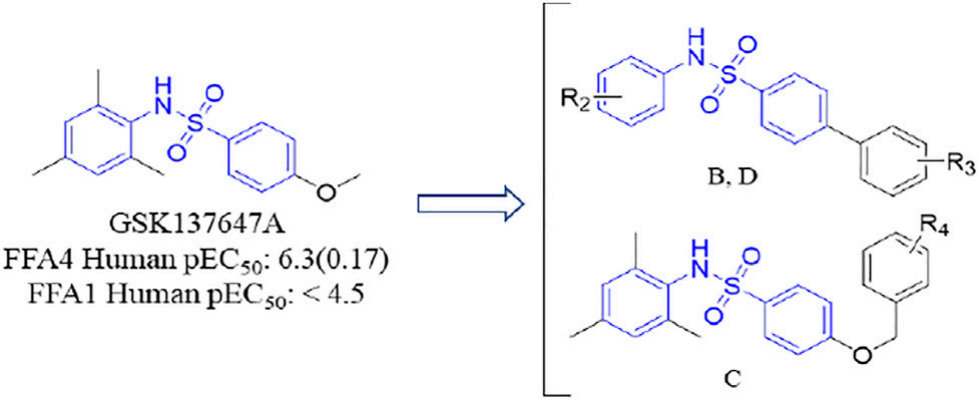
The design strategy of GPR120 small-molecule agonists.

**SCHEME 4:**
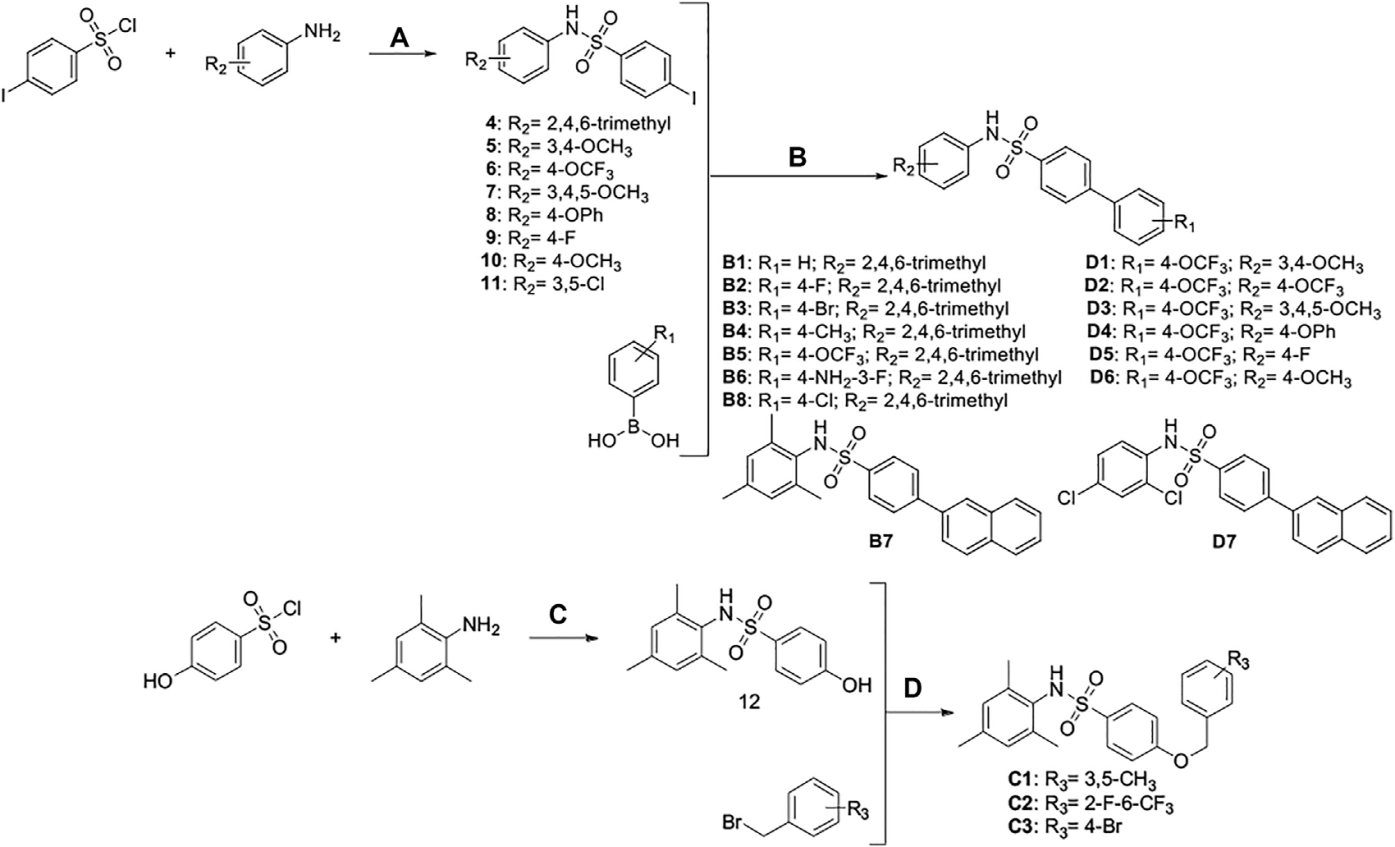
Synthesis and preparation of the modified compounds (B1-B8; C1-C3; D1-D7). Reaction conditions and reagents: (a) pyridine, dichloromethane, 6 h; (b) 1,1′-PdCl_2_(dppf), K_2_CO_3_, N_2_, 80°C, EtOH, 1 h; (c) pyridine, dichloromethane, 6 h; (d) K_2_CO_3_, MeCN, 7 h.

As mentioned earlier, the sulfonamide agonist GSK137657A has high selectivity and activity of promoting insulin secretion. We found that its phenyl ring conjugated structure is related to the selectivity of GPR120. Based on this, starting from the core structure of GSK137647A, three new types of agonists were developed herein (Figure 3; Supplementary Table S1; Scheme 4).

### 2.6 The Biological Activity of Modified Compounds

The survival rate of modified compounds is greater than 50% at 50 μM, and in subsequent cell experiments, the concentration of the compounds does not exceed 20 μM, it is proved that all compounds have acceptable cytotoxicity (Supplementary Figure S14).

#### 2.6.1 Preliminary Screening

**FIGURE 4:**
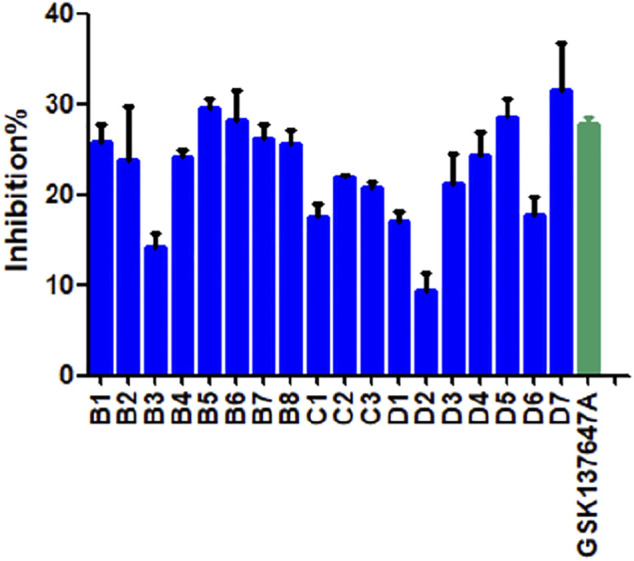
The BRET activity results of modified compounds.

We conducted preliminary activity screening of the modified 3 series (B series; C series; D series) of small-molecule agonists based on the established BRET screening method. The results present that the inhibition rates of compounds B5-7, D5, and D7 are higher than GSK137647A (Figure 4). It is proved that based on increasing the conjugated structure, the introduction of electronegative F atoms can increase the biological activity of compounds, so we have conducted in-depth research on these 5 compounds (B5, B6, B7, D5, D7).

#### 2.6.2 *In vitro* Activity Test of Preferred Compounds

**FIGURE 5:**
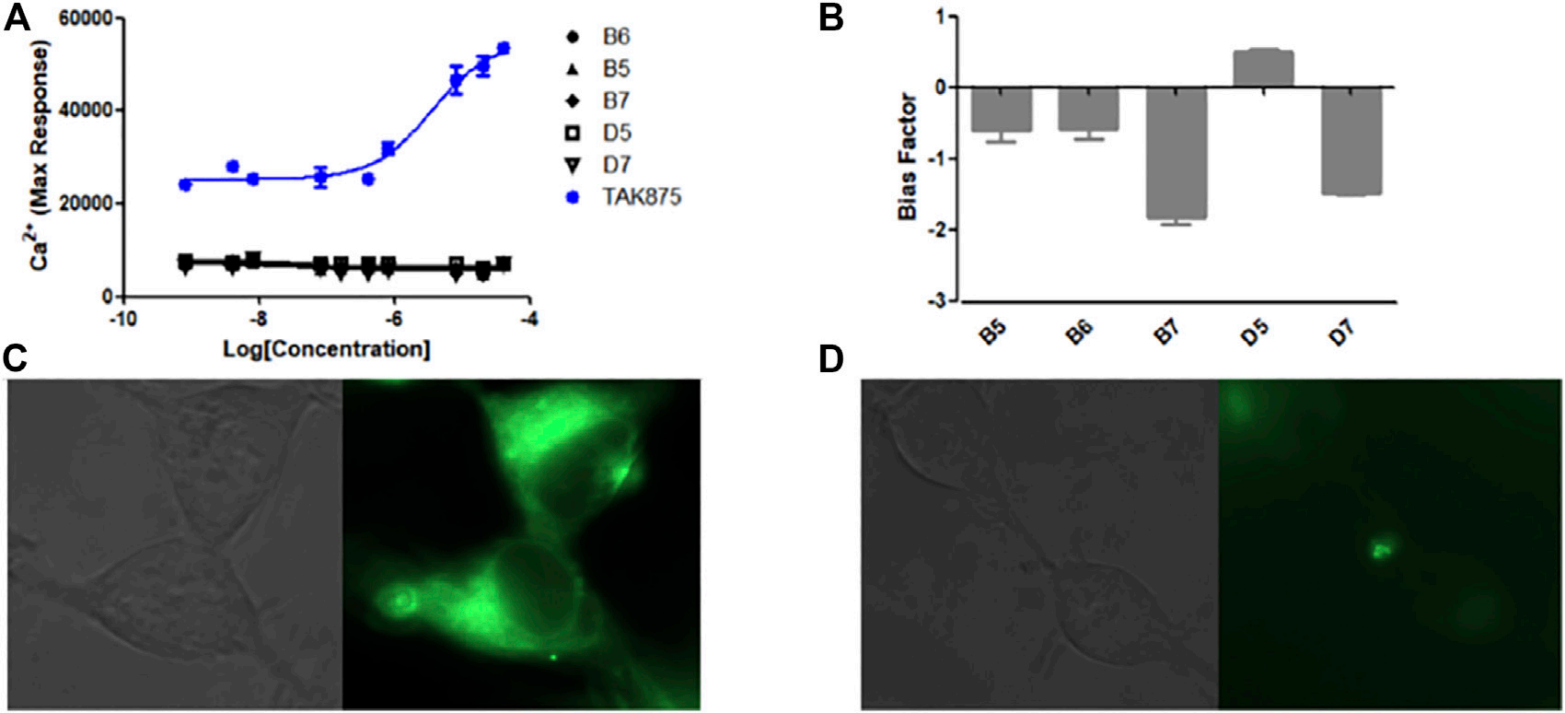
**(A)** The Ca^2+^ response of modified compounds (B5, B6, B7, D5, D7, TAK875) against GPR40. TAK875 is a positive GPR40 agonist reported in the literature; **(B)** The bias factor of compounds (B5, B6, B7, D5, D7); Exposure time was 500 ms; GFP channel; ×63 lens magnification. **(C)** Imaging result of N1 (0.5 μM) in HEK293 cells (stably transfected with GPR120); **(D)** Imaging result of N1 (0.5 μM) and D5 (10 μM) in HEK293 cells (stably transfected with GPR120).

The Ca^2+^ flow and BRET assays were performed on these five compounds (Table 3), and the results revealed that compound D5 had the best activity, and the Ca^2+^ response value of five compounds to GPR40 was much lower than TAK875 (Figure 5A). Among them, D5 is relatively biased towards the Gq pathway to promote the secretion of GLP-1 (Figure 5B). In consequence, we chose D5 for the animal blood glucose experiments.

**TABLE 3:** The pharmacological activities of selected compounds.
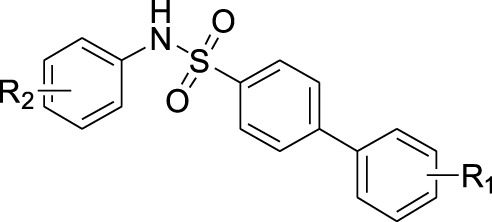


No.	Structure	FFA4 (Ca^2+^ ^a^pEC_50_)	bE_max_	FFA4 (β-arrestin2 ^a^pEC_50_)	^b^E_max_	FFA1 (Ca^2+^ ^a^pEC_50_)
B5	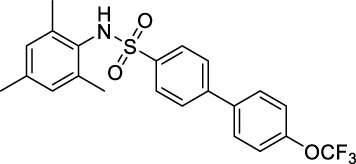	7.09 ± 0.30	1.27	7.36 ± 0.02	0.790	<4.3
B6	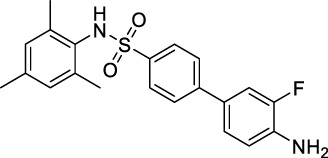	6.69 ± 0.58	1.25	6.88 ± 0.33	0.881	<4.3
B7	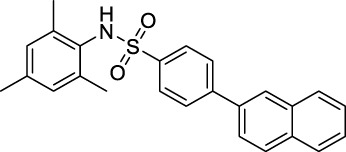	6.86 ± 0.53	0.285	7.71 ± 0.66	0.894	<4.3
D5	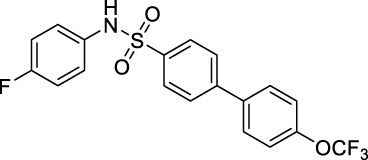	8.27 ± 0.44	1.22	7.46 ± 0.21	1.12	<4.3
D7	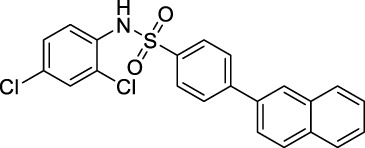	6.89 ± 0.14	0.752	7.78 ± 0.01	1.17	<4.3

a pEC_50_ is a biological activity value obtained from the Ca^2+^ ion activity test and the *β*-arrestin-2 activity test method.

b E_max_: The positive compound GSK137647A (Ca^2+^ pEC_50_: 7.35 ± 0.59; β-arrestin2: 6.75 ± 0.48) was used as a standard.

### 2.7 Competitive Cell Imaging of D5 and Fluorescent Ligand N1

The experimental results show (Figure 5C) that only incubating N1 with cells can emit strong fluorescence. However, after incubating an excessive amount of D5 with N1, the fluorescence intensity decreased significantly (Figure 5D), which to a certain extent indicates the binding ability of compound D5 and GPR120.

### 2.8 The Animal Blood Glucose Experiments

**FIGURE 6:**
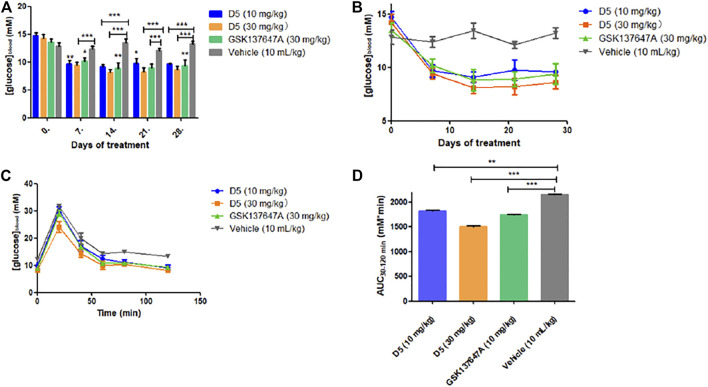
**(A, B)** The fasting blood glucose values of db/db mice after 4 weeks of continuous administration. ***p ≤ 0.001; **p ≤ 0.01; *p ≤ 0.05; **(C, D)** Blood glucose at a different time (5, 10, 30, 60, 120 min) after oral administration of glucose in mice.

Compared with GSK137647A, compound D5 can achieve the same blood glucose-lowering level at a low dose (10 mg/kg), while it can reach a higher blood-glucose-lowering level at a high dose (30 mg/kg) (Figures 6A,B). Simultaneously, the glucose tolerance test (Figures 6C,D) further proved the glucose-lowering ability of D5 in db/db mice. D5 at a low dose (10 mg/kg) reduced blood glucose AUC_0–120 min_ by 15.3%; it reduced blood glucose AUC_0–120 min_ by 30.3% at a high dose (30 mg/kg), which was higher than GSK137647A (AUC_0-120 min_ decreased by 19.3%).

The weight changes showed that the weight of the low-dose administration group (10 mg/kg) reached a stable level in the fourth week (Supplementary Figure S15); the weight of the high-dose administration group (30 mg/kg) decreased in the fourth week, which further proved the therapeutic effect of compound D5 on T2DM model mice.

## 3 Conclusion and Perspective

In this study, we designed and synthesized a series of fluorescent ligands with good fluorescence performance and excellent biological activity to GPR120. The representative fluorescent ligand N1 can be successfully used to localize and visualize GPR120 at the nanomolar level. Subsequently, we utilized BRET competitive experiment-based fluorescent ligand N1 as a fluorescent competitive substrate to establish a method that can successfully screen small-molecule agonists of GPR120. Then, we modified 3 series of small-molecule agonists based on the core of GSK137647A and conducted preliminary activity screening through the BRET activity screening method we established. Further pharmacological tests were conducted on compounds (B5, B6, B7, D5, D7) with good preliminary screening results. Compound D5 was identified as a potent GPR120 agonist with high activity and selectivity, importantly, which exhibited a significant glucose-lowering effect *in vivo*. The structure-activity relationship shows that the introduction of biphenyl conjugated structure and the electronegative F atom to the pharmacophore can increase the biological activity of the compound. This study demonstrates the potential of the fluorescent ligand-based BRET method in the screening of small-molecule agonists of GPR120. It is anticipated that such an activity screening strategy could be similarly extended to other targets of GPCRs to provide a novel method.

## 4 Experimental Section

### 4.1 Chemistry

#### 4.1.1 Reagents

All reagents are chemical pure or analytical pure, and the water used in chemical experiments is distilled water. Unless otherwise specified, reagents and solvents were used without further purification. The melting points were determined on an RY-1G melting point apparatus (uncorrected temperature before use). NMR spectra were obtained on a Bruker AV-400 spectrometer with 400 MHz for ^1^H NMR and 100 MHz for ^13^C NMR. Mass spectra (ESI mode) and high-resolution mass spectra (HR-MS) were conducted in the Analysis and Test Center of Shandong University, Jinan, China. The HPLC analysis of the compounds was performed on Agilent Technologies 1260 series high-performance liquid chromatography (Agilent Technologies Inc., Santa Clara, CA, United States) using a C18 reversed-phase column (250 mm × 4.6 mm, 5 μm, Phenomenex Inc., Torrance, CA, United States).

#### 4.1.2 Synthesis Steps

##### 4.1.2.1 4-iodo-*N*-mesitylbenzenesulfonamide (1)

2,4,6-trimethylaniline (232.07 μl, 1.65 μmol) and pyridine (399.46 μl, 4.96 μmol) were added to 10 ml of dry dichloromethane, then *p*-iodobenzene sulfonyl chloride (500 mg, 1.65 μmol) dissolved in a small amount of dichloromethane was slowly dropped into the above solution. After stirring at room temperature for 6 h, added 50 ml dichloromethane (2 × 20 ml) and 1M hydrochloric acid (2 × 10 ml) to the reaction solution for extraction, then combined the organic phases, washed with distilled water twice, and then washed with saturated brine, dried over anhydrous sodium sulfate, filtered, concentrated under reduced pressure. The crude product was purified by silica gel column chromatography with dichloromethane and methanol to afford a white solid. Yield: 520 mg, 78%. ^1^H NMR (400 MHz, DMSO) δ 9.34 (s, 1H), 7.97 (d, *J* = 7.6 Hz, 2H), 7.41 (d, *J* = 7.7 Hz, 2H), 6.83 (s, 2H), 2.19 (s, 3H), 1.90 (s, 6H). ESI-MS: m/z [M - H]^-^ calcd for C_15_H_15_INO_2_S^−^ 400.3, found 400.2.

##### 4.1.2.2 4-((2-aminoethyl)amino)-*N*-mesitylbenzenesulfonamide (2a)

Intermediate 1 (100 mg, 249.21 μmol) and cuprous iodide (4.75 mg, 24.92 μmol) were added into ethylenediamine (1.66 ml, 24.92 mmol) firstly; then the solution was heated to 100 °C for 16 h. After the solution was cooled to room temperature, concentrated most of the solution under reduced pressure at 70°C. The solution was extracted with saturated sodium bicarbonate solution (3 × 40 ml) and dichloromethane (3 × 40 ml), then it was washed with distilled water (2 × 20 ml) and saturated brine (3 × 40 ml). After that, the organic layer was combined and dried over sodium sulfate, filtered, concentrated under reduced pressure. The crude product was purified by silica gel column chromatography with dichloromethane and methanol and a small amount of triethylamine to afford a white solid. Yield: 60 mg, 72%, mp:148–149°C. ^1^H NMR (400 MHz, CDCl_3_) δ 7.47 (d, *J* = 8.3 Hz, 1H), 6.82 (s, 1H), 6.54 (d, *J* = 8.3 Hz, 1H), 3.20 (s, 1H), 2.99 (d, *J* = 5.2 Hz, 1H), 2.24 (s, 2H), 2.04 (s, 3H). ESI-MS: m/z [M + H]^+^ calcd for C_17_H_24_N_3_O_2_S^+^ 334.5, found 334.4.

##### 4.1.2.3 4-((3-aminopropyl)amino)-*N*-mesitylbenzenesulfonamide (2b)

Intermediate 1 (100 mg, 249.21 μmol) and cuprous iodide (4.75 mg, 24.92 μmol) were added into 1,3-propylene diamine (2.08 ml, 24.92 mmol) firstly; then the solution was heated to 100°C for 20 h. After the solution was cooled to room temperature, most of the solution was concentrated under reduced pressure at 70°C. The solution was extracted with saturated sodium bicarbonate solution (3 × 40 ml) and dichloromethane (3 × 40 ml), and it was washed with distilled water (2 × 20 ml) and saturated brine (3 × 40 ml). After that, the organic layer was combined and dried over sodium sulfate, filtered, concentrated under reduced pressure. The crude product was purified by silica gel column chromatography with dichloromethane and methanol and a small amount of triethylamine to afford a white solid. Yield: 48 mg, 55%, mp:151–152°C. ^1^H NMR (400 MHz, CDCl_3_) δ 7.46 (d, *J* = 8.2 Hz, 1H), 6.81 (s, 1H), 6.51 (d, *J* = 8.2 Hz, 1H), 3.25 (t, *J* = 6.3 Hz, 1H), 2.89 (t, *J* = 6.3 Hz, 1H), 2.24 (s, 1H), 2.04 (s, 3H), 1.82–1.73 (m, 1H). ESI-MS: m/z [M + H]^+^ calcd for C_18_H_26_N_3_O_2_S^+^ 348.5, found 348.5.

##### 4.1.2.4 4-((4-aminobutyl)amino)-*N*-mesitylbenzenesulfonamide (2c)

Intermediate 1 (100 mg, 249.21 μmol) and cuprous iodide (4.75 mg, 24.92 μmol) were added into 1,4-butanediamine (2.51 ml, 24.92 mmol) firstly; then the solution was heated to 100°C for 20 h. After the solution was cooled to room temperature, most of the solution was concentrated under reduced pressure at 70°C. The solution was extracted with saturated sodium bicarbonate solution (3 × 40 ml) and dichloromethane (3 × 40 ml), and it was washed with distilled water (2 × 20 ml) and saturated brine (3 × 40 ml). After that, the organic layer was combined and dried over sodium sulfate, filtered, concentrated under reduced pressure. The crude product was purified by silica gel column chromatography with dichloromethane and methanol and a small amount of triethylamine to afford a white solid. Yield: 40 mg, 44%, mp:152–153°C. ^1^H NMR (400 MHz, CDCl_3_) δ 7.45 (d, *J* = 8.3 Hz, 1H), 6.80 (s, 1H), 6.49 (d, *J* = 8.4 Hz, 1H), 5.30 (s, 1H), 3.13 (t, *J* = 6.4 Hz, 1H), 2.76 (t, *J* = 6.4 Hz, 1H), 2.23 (s, 2H), 2.03 (s, 3H), 1.70–1.62 (m, 1H), 1.60–1.48 (m, 1H). ESI-MS: m/z [M - H]^-^ calcd for C_19_H_26_N_3_O_2_S^−^ 360.5, found 360.5.

##### 4.1.2.5 6-(Dimethylamino)-1H,3H-benzo[de]isochromene-1,3-dione (3)

To a solution of 4-bromo-1,8-naphthalenedicarboxylic anhydride (1.5 g, 5.41 mmol) in DMF (40 ml) was added dimethylamine aqueous solution (2.0 g, 43.2 mmol) and copper sulfate pentahydrate (130.26 mg, 541.37 μmol). The reaction mixture was heated at 150°C for 12 h. The temperature was then allowed to reach room temperature. The reaction mixture was concentrated under reduced pressure at 70°C, diluted with ice ethanol, and filtered. The filtrate was dried to give intermediate 1 as a yellow solid. Yield: 1.10 g, 84%, m.p. 191–192°C. ^1^H NMR (400 MHz, DMSO-*d*_*6*_) δ 8.59 (d, *J* = 8.5 Hz, 1H), 8.46 (d, *J* = 7.2 Hz, 1H), 8.32 (d, *J* = 8.3 Hz, 1H), 7.76 (t, 1H), 7.20 (d, *J* = 8.4 Hz, 1H), 3.17 (s, 6H). ESI-MS: m/z [M - H]^-^ calcd for C_19_H_26_N_3_O_2_S^−^ 360.5, found 360.5.

##### 4.1.2.6 4-((2-(6-(Dimethylamino)-1,3-dioxo-1H-benzo[de]isoquinolin-2(3H)-yl)ethyl)amino)-*N*-mesitylbenzenesulfonamide (N1)

Intermediate 1a (50 mg, 149.95 μmol) and intermediate 1 (39.79 mg, 164.94 μmol) were added to 15 ml of absolute ethanol, then the reaction solution was heated to 80°C for 7 h. After the solution was cooled to room temperature, concentrated most of the solution under reduced pressure at 40°C. The crude product was purified by silica gel column chromatography with dichloromethane and methanol to afford a yellow solid. Yield: 62 mg, 74%, m.p. 230–231°C. ^1^H NMR (400 MHz, CDCl_3_) δ 8.58 (d, *J* = 7.2 Hz, 1H), 8.47 (t, 2H), 7.67 (t, *J* = 7.7 Hz, 1H), 7.41 (d, *J* = 8.2 Hz, 2H), 7.12 (d, *J* = 8.2 Hz, 1H), 6.77 (s, 2H), 6.55 (d, *J* = 8.3 Hz, 2H), 5.71 (s, 1H), 5.03 (s, 1H), 4.50 (t, *J* = 5.6 Hz, 2H), 3.60–3.50 (m, 2H), 3.13 (s, 6H), 2.22 (s, 3H), 1.98 (s, 6H). ^13^C NMR (100 MHz, DMSO) δ 164.43, 163.63, 157.02, 152.30, 137.85, 136.30, 132.98, 132.08, 131.67, 131.10, 130.21, 129.32, 128.80, 127.90, 125.46, 124.65, 122.78, 113.70, 113.43, 111.36, 44.92, 38.60, 20.89, 18.82. ESI-HRMS: m/z [M+H]^+^ calcd for C_31_H_33_N_4_O_4_S^+^ 557.2223, found 557.2215. HPLC, t_R_ = 13.564 min, 97.1% Purity, mobile phase: methanol-water (75:25, *v/v*), λ = 280 nm.

##### 4.1.2.7 4-((3-(6-(Dimethylamino)-1,3-dioxo-1H-benzo[de]isoquinolin-2(3H)-yl)propyl)amino)-*N*-mesitylbenzenesulfonamide (N2)

Intermediate 1b (80 mg, 230.23 μmol) and intermediate 1 (55.54 mg, 230.23 μmol) were added to 15 ml of absolute ethanol, then the reaction solution was heated to 80°C for 7 h. After the solution was cooled to room temperature, concentrated most of the solution under reduced pressure at 40°C. The crude product was purified by silica gel column chromatography with dichloromethane and methanol to afford a yellow solid. Yield: 46 mg, 35%, m.p. 103–104°C. ^1^H NMR (400 MHz, CDCl_3_) δ 8.59 (d, *J* = 7.2 Hz, 1H), 8.48 (t, *J* = 8.4 Hz, 2H), 7.69 (t, *J* = 7.7 Hz, 1H), 7.45 (d, *J* = 8.2 Hz, 2H), 7.13 (d, *J* = 8.1 Hz, 1H), 6.82 (s, 2H), 6.57 (d, *J* = 8.4 Hz, 2H), 5.74 (s, 1H), 5.00 (s, 1H), 4.29 (t, *J* = 6.4 Hz, 2H), 3.26–3.21 (m, 2H), 3.13 (s, 6H), 2.24 (s, 3H), 2.09–2.01 (m, 8H). ^13^C NMR (100 MHz, DMSO-*d*_*6*_) δ 164.43, 163.83, 157.16, 152.29, 137.99, 136.18, 132.84, 132.02, 131.93, 131.06, 130.12, 129.48, 128.87, 127.53, 125.39, 124.61, 122.96, 113.68, 113.45, 111.70, 44.81, 40.84, 38.07, 20.88, 18.97. ESI-HRMS: m/z [M+H]^+^ calcd for C_32_H_35_N_4_O_4_S^+^ 571.2379, found 571.2376. HPLC, t_R_ = 8.047 min, 96.0% Purity, mobile phase: methanol-water (75:25, *v/v*), λ = 280 nm.

##### 4.1.2.8 4-((4-(6-(Dimethylamino)-1,3-dioxo-1H-benzo[de]isoquinolin-2(3H)-yl)butyl)amino)-*N*-mesitylbenzenesulfonamide (N3)

Intermediate 1c (30 mg, 82.99 μmol) and intermediate 1 (20.02 mg, 82.99 μmol) were added to 15 ml of absolute ethanol, then the reaction solution was heated to 80°C for 7 h. After the solution was cooled to room temperature, concentrated most of the solution under reduced pressure at 40°C. The crude product was purified by silica gel column chromatography with dichloromethane and methanol to afford a yellow solid. Yield: 25 mg, 51%, m.p. 184–185°C. ^1^H NMR (400 MHz, CDCl_3_) δ 8.58 (d, *J* = 7.2 Hz, 1H), 8.47 (t, *J* = 11.7, 8.7 Hz, 2H), 7.67 (t, *J* = 7.9 Hz, 1H), 7.43 (d, *J* = 8.1 Hz, 2H), 7.12 (d, *J* = 8.2 Hz, 1H), 6.80 (s, 2H), 6.52 (d, *J* = 8.1 Hz, 2H), 5.72 (s, 1H), 4.43 (s, 1H), 4.23 (t, *J* = 7.0 Hz, 2H), 3.30–3.21 (m, 2H), 3.12 (s, 6H), 2.23 (s, 3H), 2.02 (s, 6H), 1.91–1.82 (m, 2H), 1.79–1.72 (m, 2H). ^13^C NMR (100 MHz, DMSO-*d*_*6*_) δ 164.21, 163.46, 157.06, 152.56, 137.64, 136.19, 132.92, 132.12, 131.80, 131.05, 130.15, 129.18, 128.72, 127.22, 125.50, 124.76, 122.67, 113.79, 113.36, 111.25, 44.94, 42.54, 26.66, 25.92, 20.78, 18.69. ESI-HRMS: m/z [M+H]^+^ calcd for C_33_H_37_N_4_O_4_S^+^ 585.2536, found 585.2534. HPLC, t_R_ = 8.178 min, 97.1% Purity, mobile phase: methanol-water (75:25, *v/v*), λ = 254 nm.

##### 4.1.2.9 4-Iodo-*N*-Mesitylbenzenesulfonamide (4)

2,4,6-trimethylaniline (232.07 μl, 1.65 μmol) and pyridine (399.46 μl, 4.96 μmol) were added to 10 ml of dry dichloromethane, followed by *p*-iodobenzene sulfonyl chloride (500 mg, 1.65 μmol) dissolved in a small amount of dichloromethane was slowly dropped into the above solution. After stirring at room temperature for 6 h, added 50 ml dichloromethane (2 × 20 ml) and 1 M hydrochloric acid (2 × 10 ml) to the reaction solution for extraction, then combined the organic phases, washed with distilled water twice, and then washed with saturated brine, dried over anhydrous sodium sulfate, filtered, concentrated under reduced pressure. The crude product was purified by silica gel column chromatography with dichloromethane and methanol to afford a white solid. Yield: 520 mg, 78%, m.p. 178–179°C. ^1^H NMR (400 MHz, DMSO-*d*_*6*_) δ 9.34 (s, 1H), 7.97 (d, *J* = 7.6 Hz, 2H), 7.41 (d, *J* = 7.7 Hz, 2H), 6.83 (s, 2H), 2.19 (s, 3H), 1.90 (s, 6H). ESI-MS: m/z [M - H]^-^ calcd for C_15_H_15_INO_2_S^−^ 400.3, found 400.2.

##### 4.1.2.10 *N*-(3,4-Dimethoxyphenyl)-4-Iodobenzenesulfonamide (5)

From 3,4-dimethoxyaniline (75.95 mg, 495.85 μmol) and 4-iodobenzenesulfonyl chloride (100 mg, 330.57 μmol), according to the synthesis method of intermediate 2 (R2 = 2,4,6-trimethyl), a white solid can be obtained. Yield: 131 mg, 91%, mp:142–143°C. ^1^H NMR (400 MHz, DMSO-*d*_*6*_) δ 9.96 (s, 1H), 7.93 (d, *J* = 8.5 Hz, 2H), 7.45 (d, *J* = 8.5 Hz, 2H), 6.80 (d, *J* = 8.6 Hz, 1H), 6.66 (d, *J* = 2.4 Hz, 1H), 6.54 (dd, *J* = 8.6, 2.4 Hz, 1H), 3.65 (d, *J* = 12.0 Hz, 6H). ESI-MS: m/z [M - H]^-^ calcd for C_14_H_13_INO_4_S^−^ 418.0, found 418.0.

##### 4.1.2.11 4-Iodo*-N*-(3,4,5-Trimethoxyphenyl)benzenesulfonamide (7)

From 3,4,5-Trimethoxyaniline (72.67 mg, 396.68 μmol) and 4-iodobenzenesulfonyl chloride (80 mg, 264.45 μmol), according to the synthesis method of intermediate 2 (R2 = 2,4,6-trimethyl), a white solid can be obtained. Yield: 110 mg, 89.4%, mp:152–153°C. ^1^H NMR (400 MHz, DMSO-*d*_*6*_) δ 10.18 (s, 1H), 7.96 (d, *J* = 8.5 Hz, 2H), 7.52 (d, *J* = 8.5 Hz, 2H), 6.37 (s, 2H), 3.66 (s, 7H), 3.57 (s, 3H). ESI-MS: m/z [M - H]^-^ calcd for C_15_H_15_INO_5_S^−^ 448.0, found 448.0.

##### 4.1.2.12 4-Iodo-*N*-(4-(trifluoromethoxy)phenyl)benzenesulfonamide (6)

From 4-trifluoromethoxyaniline (70.26 mg, 396.68 μmol) and 4-iodobenzenesulfonyl chloride (80 mg, 264.45 μmol), according to the synthesis method of intermediate 2 (R2 = 2,4,6-trimethyl), a white solid can be obtained. Yield: 102 mg, 87%, mp:149–150°C. ^1^H NMR (400 MHz, DMSO-*d*_*6*_) δ 10.60 (s, 1H), 7.96 (d, *J* = 8.5 Hz, 2H), 7.51 (d, *J* = 8.5 Hz, 2H), 7.28 (d, *J* = 8.6 Hz, 2H), 7.17 (d, *J* = 9.0 Hz, 2H). ESI-MS: m/z [M - H]^-^ calcd for C_13_H_9_F_3_INO_3_S^−^ 441.9, found 442.1.

##### 4.1.2.13 4-Iodo-*N*-(4-phenoxyphenyl)benzenesulfonamide (8)

From 4-phenoxyphenyl aniline (91.84 mg, 495.85 μmol) and 4-iodobenzenesulfonyl chloride (100 mg, 330.57 μmol), according to the synthesis method of intermediate 2 (R2 = 2,4,6-trimethyl), a white solid can be obtained. Yield: 137 mg, 85.9%, mp:162–163°C. ^1^H NMR (400 MHz, DMSO-*d*_*6*_) δ 10.23 (s, 1H), 7.95 (d, *J* = 8.5 Hz, 1H), 7.47 (d, *J* = 8.5 Hz, 1H), 7.37 (t, *J* = 8.0 Hz, 1H), 7.15–7.02 (m, 2H), 6.92 (dd, *J* = 8.2, 5.6 Hz, 3H). ESI-MS: m/z [M - H]^-^ calcd for C_18_H_13_INO_3_S^−^ 450.0, found 450.3.

##### 4.1.2.14 *N*-(4-Fluorophenyl)-4-Iodobenzenesulfonamide (9)

From 4-fluoroaniline (176.32 mg, 1.59 mmol) and 4-iodobenzenesulfonyl chloride (320 mg, 1.06 mmol), according to the synthesis method of intermediate 2 (R2 = 2,4,6-trimethyl), a white solid can be obtained. Yield: 360 mg, 90.23%, mp:146–147°C. ^1^H NMR (400 MHz, DMSO-*d*_*6*_) δ 10.30 (s, 1H), 7.94 (d, *J* = 8.5 Hz, 2H), 7.45 (d, *J* = 8.5 Hz, 2H), 7.20–6.88 (m, 4H). ESI-MS: m/z [M - H]^-^ calcd for C_12_H_8_FINO_2_S^−^ 375.9, found 376.1.

##### 4.1.2.15 4-Iodo-*N*-(4-methoxyphenyl)benzenesulfonamide(10)

From 3*-*methoxyaniline (61.07 mg, 495.85 μmol) and 4-iodobenzenesulfonyl chloride (100 mg, 330.57 μmol), according to the synthesis method of intermediate 2 (R2 = 2,4,6-trimethyl), a white oil can be obtained. Yield: 106 mg, 82.3%. ^1^H NMR (400 MHz, DMSO-*d*_*6*_) δ 10.37 (s, 1H), 7.95 (d, *J* = 8.5 Hz, 2H), 7.52 (d, *J* = 8.5 Hz, 2H), 7.14 (t, *J* = 8.4 Hz, 1H), 6.72–6.57 (m, 4H). ESI-MS: m/z [M - H]^-^ calcd for C_13_H_11_INO_3_S^−^ 388.0, found 388.4.

##### 4.1.2.16 *N*-(3,5-Dichlorophenyl)-4-iodobenzenesulfonamide (11)

From 3,5-Dichloroaniline (160.67 mg, 991.7 μmol) and 4-iodobenzenesulfonyl chloride (200 mg, 661.13 μmol), according to the synthesis method of intermediate 2 (R2 = 2,4,6-trimethyl), a white solid can be obtained. Yield: 246 mg, 83.8%, mp:158–159°C. ^1^H NMR (400 MHz, DMSO-*d*_*6*_) δ 10.26 (s, 1H), 7.96 (d, *J* = 8.5 Hz, 2H), 7.61 (d, *J* = 2.4 Hz, 1H), 7.47–7.37 (m, 3H), 7.26 (d, *J* = 8.7 Hz, 1H). ESI-MS: m/z [M - H]^-^ calcd for C_12_H_7_Cl_2_INO_2_S^−^ 425.9, found 426.0.

##### 4.1.2.17 *N*-Mesityl-[1,1′-biphenyl]-4-sulfonamide (B1)

Intermediate 2 (200 mg, 479.27 μmol), potassium carbonate (198.71 mg, 1.44 mmol), [1,1′-bis(diphenylphosphino) dicene Iron] palladium dichloride (35.07 mg, 47.93 μmol) and phenylboronic acid (58.44 mg, 479.27 μmol) were added to the mixed liquor of 1.6 ml of distilled water and 8 ml of absolute ethanol, pumped off nitrogen three times, heated to 80°C under the nitrogen protection for 1 h. After the solution was cooled to room temperature, concentrated most of the solution under reduced pressure at 40°C. The crude product was purified by silica gel column chromatography with Petroleum ether and ethyl acetate to afford white solids. Yield: 150 mg, 85.2%, mp:165–166°C. ^1^H NMR (400 MHz, DMSO-*d*_*6*_) δ 9.28 (s, 1H), 7.89 (d, *J* = 8.4 Hz, 2H), 7.74 (t, *J* = 7.9 Hz, 4H), 7.52 (t, *J* = 7.5 Hz, 2H), 7.44 (t, *J* = 7.3 Hz, 1H), 6.82 (s, 2H), 2.19 (s, 3H), 1.93 (s, 6H). ^13^C NMR (100 MHZ, DMSO-*d*_*6*_) δ 144.33, 141.22, 138.82, 137.84, 136.82, 131.13, 129.62, 129.49, 129.02, 127.72, 127.59, 127.48, 20.76, 18.52. ESI-HRMS: m/z [M+H]^+^ calcd for C_21_H_22_NO_2_S^+^ 352.1371, found 352.1378. HPLC, t_R_ = 8.896 min, 99.8% Purity, mobile phase: acetonitrile-water (75:25, *v/v*), λ = 254 nm.

##### 4.1.2.18 4′-Fluoro-*N*-Mesityl-[1,1′-Biphenyl]-4-Sulfonamide (B2)

From intermediate 2 (150 mg, 359.45 μmol) and 4-fluorophenylboronic acid (50.29 mg, 359.45 μmol), according to the synthesis method of B1, a white solid can be obtained. Yield: 112 mg, 80.8%, mp:214–215 °C. ^1^H NMR (400 MHz, DMSO-*d6*) δ 9.29 (s, 1H), 7.90 (d, *J* = 8.5 Hz, 2H), 7.79 (d, *J* = 8.6 Hz, 2H), 7.73 (d, *J* = 8.4 Hz, 2H), 7.57 (d, *J* = 8.5 Hz, 2H), 6.82 (s, 2H), 2.19 (s, 3H), 1.92 (s, 5H). ^13^C NMR (100 MHz, DMSO-d6) δ 142.95, 141.54, 137.82, 137.62, 136.84, 133.96, 131.08, 129.58, 129.49, 129.29, 127.74, 127.64, 20.77, 18.72. ESI-HRMS: m/z [M+K]^+^ calcd for C_21_H_20_FNO_2_SK^+^ 408.0836, found 408.0838. HPLC, t_R_ = 10.863 min, 99.0% Purity, mobile phase: acetonitrile-water (75:25, *v/v*), λ = 254 nm.

##### 4.1.2.19 4′-Bromo-*N*-Mesityl-[1,1′-Biphenyl]-4-Sulfonamide (B3)

From intermediate 2 (150 mg, 359.45 μmol) and 4-bromophenylboronic acid (72.19 mg, 359.45 μmol), according to the synthesis method of B1, a white solid can be obtained. Yield: 145 mg, 90.4%, mp:176–177°C. ^1^H NMR (400 MHz, DMSO-*d*_*6*_) δ 9.29 (s, 1H), 7.89 (d, *J* = 8.5 Hz, 2H), 7.78–7.66 (m, 6H), 6.82 (s, 2H), 2.19 (s, 3H), 1.93 (s, 6H). ^13^C NMR (100 MHZ, DMSO-*d*_*6*_) δ 142.96, 141.55, 138.01, 137.80, 136.81, 132.51, 131.09, 129.58, 129.49, 127.69, 122.61, 20.78, 18.51. ESI-HRMS: m/z [M+H]^+^ calcd for C_21_H_21_BrNO_2_S^+^ 430.0476, found 430.0468. HPLC, t_R_ = 15.161 min, 95.3% Purity, mobile phase: acetonitrile-water (70:30, *v/v*), λ = 300 nm.

##### 4.1.2.20 *N*-Mesityl-4′-Methyl-[1,1′-Biphenyl]-4-Sulfonamide (B4)

From intermediate 2 (150 mg, 359.45 μmol) and 4-methylphenylboronic acid (48.87 mg, 359.45 μmol), according to the synthesis method of B1, a white solid can be obtained. Yield: 117 mg, 85.3%, mp:195–196°C. ^1^H NMR (400 MHz, DMSO-*d*_*6*_) δ 9.25 (s, 1H), 7.86 (d, *J* = 8.5 Hz, 1H), 7.68 (dd, *J* = 18.8, 8.3 Hz, 2H), 7.32 (d, *J* = 8.0 Hz, 1H), 6.82 (s, 1H), 2.37 (s, 1H), 2.19 (s, 2H), 1.93 (s, 3H). ^13^C NMR (100 MHZ, DMSO-*d*_*6*_) δ 144.23, 140.87, 138.56, 137.83, 136.79, 135.90, 131.14, 130.20, 129.48, 127.57, 127.36, 127.29, 21.18, 20.88, 18.82. ESI-HRMS: m/z [M+H]^+^ calcd for C_22_H_24_NO_2_S^+^ 366.1528, found 366.1449. HPLC, t_R_ = 10.444 min, 99.3% Purity, mobile phase: acetonitrile-water (75:25, *v/v*), λ = 254 nm.

##### 4.1.2.21 *N*-Mesityl-4′-(Trifluoromethoxy)-[1,1′-Biphenyl]-4-Sulfonamide (B5)

From intermediate 2 (60 mg, 143.78 μmol) and 4-trifluoromethoxyphenylboronic acid (29.61 mg, 143.78 μmol), according to the synthesis method of B1, a white solid can be obtained. Yield: 57 mg, 87.8%, mp:169–170°C. ^1^H NMR (400 MHz, DMSO-*d*_*6*_) δ 9.30 (s, 1H), 7.90 (t, *J* = 8.0 Hz, 4H), 7.74 (d, *J* = 8.3 Hz, 2H), 7.51 (d, *J* = 8.4 Hz, 2H), 6.82 (s, 2H), 2.19 (s, 3H), 1.93 (s, 6H). ^13^C NMR (100 MHz, DMSO-*d*_*6*_) δ 149.03 (s), 142.83 (s), 141.65 (s), 138.13 (s), 137.83 (s), 136.84 (s), 131.04 (s), 129.53 (s), 129.49 (s), 127.95 (s), 127.63 (s), 122.07 (s), 120.52 (q, *J* = 284.0 Hz), 20.87 (s), 18.80 (s). ESI-HRMS: m/z [M+H]^+^ calcd for C_22_H_21_F_3_NO_3_S^+^ 436.1194, found 436.1184. HPLC, t_R_ = 12.019 min, 99.1% Purity, mobile phase: acetonitrile-water (75:25, *v/v*), λ = 254 nm.

##### 4.1.2.22 4′-Amino-3′-Fluoro-*N*-Mesityl-[1,1′-Biphenyl]-4-Sulfonamide (B6)

From intermediate 2 (100 mg, 239.63 μmol) and 4-amino-3-fluorophenylboronic acid (37.13 mg, 239.63 μmol), according to the synthesis method of B1, a white solid can be obtained. Yield: 70 mg, 72.9%, mp:176–177°C. ^1^H NMR (400 MHz, DMSO-*d*_*6*_) δ 9.18 (s, 1H), 7.79 (d, *J* = 8.5 Hz, 2H), 7.62 (d, *J* = 8.5 Hz, 2H), 7.48 (dd, *J* = 13.1, 1.9 Hz, 1H), 7.36 (dd, *J* = 8.3, 1.9 Hz, 1H), 6.93–6.73 (m, 3H), 5.49 (s, 2H), 2.19 (s, 3H), 1.92 (s, 6H). ^13^C NMR (100 MHz, DMSO-*d*_*6*_) δ 151.25 (d, *J* = 237.1 Hz), 143.56 (s), 139.78 (s), 137.84 (s), 137.65 (d, *J* = 13.0 Hz), 136.74 (s), 131.20 (s), 129.46 (s), 127.50 (s), 126.15 (s), 126.08 (d, *J* = 6.5 Hz), 123.62 (d, *J* = 2.3 Hz), 116.81 (d, J = 5.2 Hz), 113.74 (d, J = 19.3 Hz), 20.88 (s), 18.83 (s). ESI-HRMS: m/z [M+H]^+^ calcd for C_21_H_22_FN_2_O_2_S^+^ 385.1386, found 385.1371. HPLC, t_R_ = 5.114 min, 98.2% Purity, mobile phase: acetonitrile-water (75:25, *v/v*), λ = 300 nm.

##### 4.1.2.23 *N*-Mesityl-4-(Naphthalen-2-yl)benzenesulfonamide (B7)

From intermediate 2 (60 mg, 143.78 μmol) and 2-naphthylbenzeneboronic acid (24.73 mg, 143.78 μmol), according to the synthesis method of B1, a white solid can be obtained. Yield: 48 mg, 80%, mp:211–212°C. ^1^H NMR (400 MHz, DMSO-*d*_*6*_) δ 9.31 (s, 1H), 8.36 (s, 1H), 8.05 (dd, *J* = 8.3, 6.9 Hz, 4H), 8.00–7.95 (m, 1H), 7.92 (dd, *J* = 8.6, 1.7 Hz, 1H), 7.78 (d, *J* = 8.4 Hz, 2H), 7.58 (dt, *J* = 5.4, 3.3 Hz, 2H), 6.83 (s, 2H), 2.19 (s, 3H), 1.95 (s, 6H). ^13^C NMR (100 MHZ, DMSO-*d*_*6*_) δ 144.17, 141.27, 137.85, 136.83, 136.12, 133.67, 133.15, 131.14, 129.51, 129.22, 128.89, 128.00, 127.66, 127.19, 127.14, 126.61, 125.33, 20.89, 18.85. ESI-HRMS: m/z [M+H]^+^ calcd for C_25_H_24_NO_2_S^+^ 402.1528, found 402.1519. HPLC, t_R_ = 11.465 min, 99.8% Purity, mobile phase: acetonitrile-water (75:25, *v/v*), λ = 254 nm.

##### 4.1.2.24 4′-Chloro-*N*-Mesityl-[1,1′-Biphenyl]-4-Sulfonamide (B8)

From intermediate 2 (150 mg, 359.45 μmol) and 4-chlorophenylboronic acid (56.21 mg, 359.45 μmol), according to the synthesis method of B1, a white solid can be obtained. Yield: 121 mg, 83.8%, mp:214–215°C. ^1^H NMR (400 MHz, DMSO-*d*_*6*_) δ 9.30 (s, 1H), 7.89 (d, *J* = 8.4 Hz, 2H), 7.79 (d, *J* = 8.6 Hz, 2H), 7.73 (d, *J* = 8.5 Hz, 2H), 7.57 (d, *J* = 8.6 Hz, 2H), 6.82 (s, 2H), 2.18 (s, 3H), 1.92 (s, 6H). ^13^C NMR (100 MHZ, DMSO-*d*_*6*_) δ 142.96, 141.53, 137.83, 137.62, 136.85, 133.96, 131.07, 129.58, 129.49, 129.29, 127.73, 127.64, 20.77, 18.75. ESI-HRMS: m/z [M+H]^+^ calcd for C_21_H_21_ClNO_2_S^+^ 386.0982, found 386.0983. HPLC, t_R_ = 10.809 min, 99.3% Purity, mobile phase: acetonitrile-water (75:25, *v/v*), λ = 254 nm.

##### 4.1.2.25 *N*-(3,4-Dimethoxyphenyl)-4′-(Trifluoromethoxy)-[1,1′-Biphenyl]-4-Sulfonamide (D1)

From intermediate 2 (100 mg, 229.74 μmol), 4-trifluoromethoxyphenylboronic acid (47.31 mg, 229.74 μmol), according to the synthesis method of B1, a white solid can be obtained. Yield: 87 mg, 80.7%, mp:145–146°C. ^1^H NMR (400 MHz, DMSO-*d*_*6*_) δ 9.99 (s, 1H), 7.94–7.74 (m, 7H), 7.48 (d, *J* = 8.4 Hz, 3H), 6.81 (d, *J* = 8.6 Hz, 1H), 6.71 (d, *J* = 2.3 Hz, 1H), 6.61 (d, *J* = 2.3 Hz, 1H), 3.65 (d, *J* = 9.6 Hz, 8H). ^13^C NMR (100 MHz, DMSO-*d*_*6*_) δ 149.26 (s), 149.04 (s), 146.63 (s), 143.11 (s), 139.20 (s), 138.10 (s), 130.90 (s), 129.56 (s), 127.96 (s), 122.01 (s), 120.48 (q, *J* = 256.0 Hz), 114.03 (s), 112.57 (s), 106.95 (s), 56.04 (s), 55.86 (s). ESI-HRMS: m/z [M+H]^+^ calcd for C_21_H_19_F_3_NO_5_S^+^ 454.0936, found 454.0939. HPLC, t_R_ = 5.229 min, 95.3% Purity, mobile phase: acetonitrile-water (75:25, *v/v*), λ = 254 nm.

##### 4.1.2.26 4′-(Trifluoromethoxy)-*N*-(4-(Trifluoromethoxy)phenyl)-[1,1′-Biphenyl]-4-Sulfonamide (D2)

From intermediate 2 (100 mg, 217.76 μmol), 4-trifluoromethoxyphenylboronic acid (44.84 mg, 217.76 μmol), according to the synthesis method of B1, a white solid can be obtained. Yield: 94 mg, 87.5%, mp:139–140°C. ^1^H NMR (400 MHz, DMSO-*d*_*6*_) δ 10.64 (s, 1H), 7.93–7.81 (m, 1H), 7.48 (d, *J* = 8.1 Hz, 1H), 7.31–7.20 (m, 1H). ^13^C NMR (100 MHz, DMSO-*d*_*6*_) δ 149.10 (s), 144.91 (s), 143.47 (s), 138.97 (s), 137.93 (s), 137.33 (s), 129.62 (s), 128.22 (s), 127.82 (s), 122.63 (s), 122.01 (s), 121.69 (s), 120.57 (q, *J* = 256.0 Hz), 120.47 (q, *J* = 256.0 Hz). ESI-HRMS: m/z [M+H]^+^ calcd for C_20_H_14_F_6_NO_4_S^+^ 478.0548, found 478.0555. HPLC, t_R_ = 5.427 min, 98.9% Purity, mobile phase: acetonitrile-water (75:25, *v/v*), λ = 254 nm.

##### 4.1.2.27 4′-(Trifluoromethoxy)-*N*-(3,4,5-Trimethoxyphenyl)-[1,1′-Biphenyl]-4-Sulfonamide (D3)

From intermediate 2 (44.84 mg, 217.76 μmol), 4-trifluoromethoxyphenylboronic acid (44.84 mg, 217.76 μmol), according to the synthesis method of B1, a white solid can be obtained. Yield: 94 mg, 84.6%, mp:136–137°C. ^1^H NMR (400 MHz, DMSO-*d*_*6*_) δ 10.19 (s, 1H), 7.86 (d, *J* = 19.9 Hz, 6H), 7.48 (d, *J* = 8.3 Hz, 2H), 6.43 (s, 2H), 3.66 (s, 6H), 3.56 (s, 3H). ^13^C NMR (100 MHz, DMSO-*d*_*6*_) δ 153.48 (s), 149.06 (s), 143.31 (s), 139.16 (s), 138.09 (s), 134.78 (s), 133.99 (s), 129.59 (s), 128.08 (s), 128.02 (s), 122.01 (s), 120.55 (q, *J* = 256.0 Hz), 98.57 (s), 60.51 (s), 56.23 (s)^.^ ESI-HRMS: m/z [M+H]^+^ calcd for C_22_H_21_F_3_NO_6_S^+^ 484.1042, found 484.1050. HPLC, t_R_ = 9.042 min, 99.7% Purity, mobile phase: acetonitrile-water (75:25, *v/v*), λ = 254 nm.

##### 4.1.2.28 *N*-(4-Phenoxyphenyl)-4′-(Trifluoromethoxy)-[1,1′-Biphenyl]-4-Sulfonamide (D4)

From intermediate 2 (75 mg, 160.49 μmol), 4-trifluoromethoxyphenylboronic acid (33.05 mg, 160.49 μmol), according to the synthesis method of B1, a white solid can be obtained. Yield: 68 mg, 84.5%, mp:144–145°C. ^1^H NMR (400 MHz, DMSO-*d*_*6*_) δ 10.28 (s, 1H), 7.92–7.79 (m, 6H), 7.49 (d, *J* = 8.2 Hz, 2H), 7.35 (dd, *J* = 10.7, 5.2 Hz, 2H), 7.12 (t, *J* = 8.2 Hz, 3H), 6.92 (d, *J* = 8.8 Hz, 4H). ^13^C NMR (100 MHz, DMSO-*d*_*6*_) δ 157.27 (s), 153.68 (s), 149.05 (s), 143.22 (s), 139.16 (s), 138.04 (s), 133.50 (s), 130.47 (s), 129.59 (s), 128.06 (s), 127.87 (s), 123.77 (s), 123.18 (s), 122.03 (s), 120.58 (q, *J* = 250.1 Hz), 120.05 (s), 118.67 (s). ESI-HRMS: m/z [M+H]^+^ calcd for C_25_H_19_F_3_NO_4_S^+^ 486.0987, found 486.0989. HPLC, t_R_ = 9.259 min, 97.8% Purity, mobile phase: acetonitrile-water (75:25, *v/v*), λ = 280 nm.

##### 4.1.2.29 *N*-(4-Fluorophenyl)-4′-(Trifluoromethoxy)-[1,1′-Biphenyl]-4-Sulfonamide (D5)

From intermediate 2 (300 mg, 762.94 μmol), 4-trifluoromethoxyphenylboronic acid (157.11 mg, 762.94 μmol), according to the synthesis method of B1, a white solid can be obtained. Yield: 267 mg, 81.9%, mp:148–149 °C. ^1^H NMR (400 MHz, DMSO-*d*_*6*_) δ 10.32 (s, 1H), 7.91–7.77 (m, 8H), 7.48 (d, *J* = 8.2 Hz, 3H), 7.18–7.07 (m, 5H). ^13^C NMR (100 MHz, DMSO-*d*_*6*_) δ 159.58 (d, *J* = 241.2 Hz), 149.07 (s), 143.29 (s), 138.94 (s), 137.99 (s), 134.27 (d, *J* = 2.6 Hz), 129.58 (s), 128.08 (s), 127.86 (s), 123.26 (d, *J* = 8.3 Hz), 122.00 (s), 120.54 (q, *J* = 256.7 Hz), 116.42 (d, *J* = 22.6 Hz). ESI-HRMS: m/z [M+H]^+^ calcd for C_19_H_14_F_4_NO_3_S^+^ 412.0631, found 412.0643. HPLC, t_R_ = 6.639 min, 99.3% Purity, mobile phase: acetonitrile-water (75:25, *v/v*), λ = 254 nm.

##### 4.1.2.30 *N*-(4-Methoxyphenyl)-4′-(Trifluoromethoxy)-[1,1′-Biphenyl]-4-Sulfonamide (D6)

From intermediate 2 (105 mg, 259.1 μmol), 4-trifluoromethoxyphenylboronic acid (53.36 mg, 259.1 μmol), according to the synthesis method of B1, a white oil can be obtained. Yield: 102 mg, 90%. ^1^H NMR (400 MHz, DMSO-*d*_*6*_) δ 10.41 (s, 1H), 7.91–7.79 (m, 6H), 7.47 (d, *J* = 8.2 Hz, 2H), 7.14 (t, *J* = 8.3 Hz, 1H), 6.77–6.70 (m, 2H), 6.64–6.58 (m, 1H), 3.66 (s, 3H). ^13^C NMR (100 MHz, DMSO-*d*_*6*_) δ 160.16 (s), 149.05 (s), 143.30 (s), 139.35 (s), 139.16 (s), 138.03 (s), 130.57 (s), 129.58 (s), 128.12 (s), 127.88 (s), 121.99 (s), 120.54 (q, *J* = 256.5 Hz), 112.28 (s), 109.52 (s), 106.10 (s), 55.32 (s). ESI-HRMS: m/z [M+H]^+^ calcd for C_20_H_17_F_3_NO_4_S^+^ 424.0830, found 424.0840. HPLC, t_R_ = 6.258 min, 99.5% Purity, mobile phase: acetonitrile-water (75:25, *v/v*), λ = 254 nm.

##### 4.1.2.31 *N*-(2,4-Dichlorophenyl)-4-(naphthalen-2-yl)benzenesulfonamide (D7)

From intermediate 2 (100 mg, 233.61 μmol), 4-trifluoromethoxyphenylboronic acid (40.18 mg, 233.61 μmol), according to the synthesis method of B1, a white solid can be obtained. Yield: 81 mg, 81%, mp:175–176°C. ^1^H NMR (400 MHz, DMSO-*d*_*6*_) δ 10.23 (s, 1H), 8.34 (s, 1H), 8.04 (dd, *J* = 8.3, 6.5 Hz, 4H), 8.00–7.95 (m, 1H), 7.91 (d, *J* = 1.7 Hz, 1H), 7.84 (d, *J* = 8.5 Hz, 2H), 7.62–7.55 (m, 3H), 7.40 (d, *J* = 2.4 Hz, 1H), 7.33 (d, *J* = 8.7 Hz, 1H). ^13^C NMR (100 MHZ, DMSO-*d*_*6*_) δ 144.64, 139.56, 136.02, 133.64, 133.17, 131.36, 130.56, 129.88, 129.21, 128.89, 128.46, 128.08, 128.02, 127.87, 127.23, 127.15, 126.71, 125.31. ESI-HRMS: m/z [M+H]^+^ calcd for C_22_H_16_Cl_2_NO_2_S^+^ 428.0279, found 428.0293. HPLC, t_R_ = 9.622 min, 97% Purity, mobile phase: acetonitrile-water (75:25, *v/v*), λ = 254 nm.

##### 4.1.2.32 4-Hydroxy-*N*-mesitylbenzenesulfonamide (12)

2,4,6-trimethylaniline (210.59 mg, 1.56 mmol) and pyridine (246.41 mg, 3.12 mmol) were added to 10 ml of dry dichloromethane, followed by *p*-iodobenzene sulfonyl chloride (200 mg, 1.04 mmol) dissolved in a small amount of dichloromethane was slowly dropped into the above solution. After stirring at room temperature for 6 h, added 50 ml dichloromethane (2 **×** 20 ml) and 1M hydrochloric acid (2 × 10 ml) to the reaction solution for extraction, then combined the organic phases, washed with distilled water twice, and then washed with saturated brine, dried over anhydrous sodium sulfate, filtered, concentrated under reduced pressure. The crude product was purified by silica gel column chromatography with dichloromethane and methanol to afford a white solid. Yield: 256 mg, 84.6%, mp: 208–209°C. ^1^H NMR (400 MHz, DMSO-*d*_*6*_) δ 10.38 (s, 1H), 8.92 (s, 1H), 7.45 (d, *J* = 8.7 Hz, 2H), 6.90–6.77 (m, 5H), 2.18 (s, 3H), 1.91 (s, 6H). ESI-MS: m/z [M - H]^-^ calcd for C_15_H_16_NO_3_S^−^ 290.1, found 290.2.

##### 4.1.2.33 4-((3,5-Dimethylbenzyl)oxy)-*N*-mesitylbenzenesulfonamide (C1)

Intermediate 3 (100 mg, 325.3 μmol), potassium carbonate (134.87 mg, 975.9 μmol), 3,5-dimethylbenzyl bromide (97.15 mg, 487.95 μmol) were added to anhydrous acetonitrile, and after stirring for 7 h at room temperature, the solvent of the reaction solution was evaporated, washed 3 times with water, 3 times with saturated sodium chloride solution, dried with anhydrous sodium sulfate for 2 h, filtered and separated by column chromatography (petroleum ether: ethyl acetate = 5:1) to obtain a white solid. Yield: 97 mg, 70.1%, mp: 152–153°C. ^1^H NMR (400 MHz, DMSO-*d*_*6*_) δ 9.04 (s, 1H), 7.56 (d, *J* = 8.8 Hz, 2H), 7.14 (d, *J* = 8.9 Hz, 2H), 7.04 (s, 2H), 6.98 (s, 1H), 6.80 (s, 2H), 5.11 (s, 2H), 2.28 (s, 7H), 2.18 (s, 3H), 1.90 (s, 7H). ^13^C NMR (100 MHZ, DMSO-*d*_*6*_) δ 161.79, 138.00, 137.80, 136.66, 136.59, 134.32, 131.27, 129.86, 129.43, 129.02, 125.94, 70.12, 21.27, 20.76, 18.69. ESI-HRMS: m/z [M+H]^+^ calcd for C_24_H_28_NO_3_S^+^ 410.1790, found 410.1791. HPLC, t_R_ = 9.676 min, 99.5% Purity, mobile phase: acetonitrile-water (75:25, *v/v*), λ = 254 nm.

##### 4.1.2.34 4-((2-Fluoro-6-(trifluoromethyl)benzyl)oxy)-*N*-mesitylbenzenesulfonamide (C2)

From intermediate 3 (60 mg, 195.18 μmol), 2-Fluoro-6-(trifluoromethyl)benzyl bromide (75.25 mg, 292.77 μmol), according to the synthesis method of C1, a white solid can be obtained. Yield: 78 mg, 82.7%, mp:154–155°C. ^1^H NMR (400 MHz, DMSO-*d*_*6*_) δ 9.09 (s, 1H), 7.76–7.68 (m, 3H), 7.59 (d, *J* = 8.8 Hz, 2H), 7.20 (d, *J* = 8.9 Hz, 2H), 6.81 (s, 2H), 5.25 (s, 2H), 2.19 (s, 2H), 1.91 (s, 5H). ^13^C NMR (100 MHz, DMSO-*d*_*6*_) δ 162.06 (d, *J* = 248.9 Hz), 161.55 (s), 137.77 (s), 136.69 (s), 134.98 (s), 132.74 (s), 131.24 (s), 130.93 (d, *J* = 9.7 Hz), 130.53 (d, *J* = 3.8 Hz), 129.47 (s), 129.11 (s), 125.26 (d, *J* = 3.2 Hz), 123.05 (q, *J* = 3.3 Hz), 125.89–118.43 (m), 120.96 (d, *J* = 22.7 Hz). ESI-HRMS: m/z [M+H]^+^ calcd for C_23_H_22_F_4_NO_3_S^+^ 468.1257, found 468.1245. HPLC, t_R_ = 9.691 min, 99.1% Purity, mobile phase: acetonitrile-water (75:25, *v/v*), λ = 254 nm.

##### 4.1.2.35 4-((4-Bromobenzyl)oxy)-*N*-mesitylbenzenesulfonamide (C3)

From intermediate 3 (139.89 mg, 480.13 μmol), 4-bromobenzyl bromide (100 mg, 400.11 μmol), according to the synthesis method of C1, a white solid can be obtained. Yield: 147 mg, 79.8%, mp:161–162°C. ^1^H NMR (400 MHz, DMSO-*d*_*6*_) δ 9.06 (s, 1H), 7.58 (dd, *J* = 20.7, 8.6 Hz, 4H), 7.43 (d, 2H), 6.79 (s, 2H), 5.19 (s, 2H), 2.18 (s, 3H), 1.88 (s, 6H). ^13^C NMR (100 MHZ, DMSO-*d*_*6*_) δ 161.60, 137.89, 136.67, 136.25, 134.67, 131.88, 131.22, 130.44, 129.43, 129.03, 121.55, 115.70, 69.48, 21.30, 18.29. ESI-HRMS: m/z [M+H]^+^ calcd for C_22_H_23_BrNO_3_S^+^ 460.0582, found 460.0587. HPLC, t_R_ = 9.530 min, 96.8% Purity, mobile phase: acetonitrile-water (75:25, *v/v*), λ = 254 nm.

### 4.2 Biological Activity Tests

#### 4.2.1 Reagents

All chemical reagents and raw materials were purchased from the experimental consumables management platform of Shandong University (http://syhc.sdu.edu.cn), and the purity is greater than 95%. High glucose DMEM medium was purchased from Hyclone, liposome 2000 was purchased from Invitrogen, fetal calf serum was purchased from Gibco, pancreatin was purchased from Gibco, plasmid GPR120-YFP and plasmid β-arrestin2-Rluc were purchased from Jinan Boshang Biotechnology Company, coelenterazine was purchased from Shanghai Jizhi Biochemical Co., Ltd, OPTI-MEM medium was purchased from Hyclone, Poloxamer F-127 and DMSO were purchased from Amresco, Ca^2+^ probe Fluo-4, am and HBSS (D-Hanks) were purchased from Biyuntian Biotechnology Co., Ltd.

Male 8-week-old C57BL/KsJ-db/db spontaneously diabetic mice were purchased from Beijing Weishanglide Biotechnology Co., Ltd.

#### 4.2.2 Optical Property

The stocking solution of probes was diluted with acetonitrile to 40 μM, and their ultraviolet absorption spectrum was scanned with an ultraviolet spectrophotometer. Secondly, the excitation and emission spectra of probes at 10, 20, 30, and 40 μM with PBS buffer were measured with a microplate reader. Moreover, the excitation and emission spectrum were recorded in four solvents: MeCN, DMSO, H_2_O, and EA, respectively.

#### 4.2.3 CCK8 Assay for Cytotoxicity

The cytotoxicity of probes was measured in HEK293, PC-3, and CHO cells using the CCK8 method. Cells were firstly seeded into a transparent 96-well plate (100 μl, 8000 cells per well). After the cells were iron-walled, the compound solution was diluted with serum-free medium to different concentrations for the test. After a 24-h incubation, the medium was removed from the plate, and the CCK8 working solution was added. Finally, the absorbance at 450 nm was scanned after a 1-h incubation by the microplate reader.

#### 4.2.4 Ca^2+^ Flow Assay

Ca^2+^ flow activity was determined in HEK293 cells stably transfecting the GPR120-Rluc gene. After incubating the cells for 24 h, the medium was removed from the plate, and the cells were washed with HBSS buffer (without Ca^2+^ and Mg^2+^). Subsequently, a 2-μM Fluo-4 AM working solution in HBSS buffer (without Ca^2+^ and Mg^2+^) was added to the plate (40 μl per well). After incubating the cells for 20 min at 37°C, compound solution at different concentrations in HBSS buffer (per well 160 μl) was added. Followed by a 40-min incubation, the solution was removed from the plate, HEPES buffer (200 μl per well) was added, and the fluorescence intensity was detected using the microplate reader (Ex: 488 nm; Em: 520 nm) (Watterson et al., 2017).

Ca^2+^ flow activity was determined in CHO cells with transient GPR40 gene by the same method as well, in which the GPR40 agonist TAK875 was used as a positive control.

#### 4.2.5 Bioluminescence Resonance Energy Transfer Activity Test

GPR120-YFP and *β*-arresting-2-Rluc plasmids were transfected into HEK293 cells. After a 24-h incubation, the cells were seeded in a black 96-well plate (100 μl, 20,000 cells per well). Followed by a 24-h incubation, the solution was removed from the plate, and compounds with different concentrations (100 μl per well) and coelenterazine (10 μM, 100 μl per well) were added, followed by the fluorescence intensity was measured at 460 and 520 nm with the microplate reader (Christiansen et al., 2016; Hill et al., 2018).

#### 4.2.6 Fluorescence Imaging

HEK293 and PC-3 cells were seeded in fluorescent confocal dishes (5 × 10^3^ per well). After the cells were iron-walled, the medium was removed from the dishes, the cells were washed once with a serum-free medium, and the probe was added. After incubating the cells for 10 min, the confocal dishes were observed by an inverted fluorescence microscope.

#### 4.2.7 Bioluminescence Resonance Energy Transfer Saturation Experiment

HEK293 cells stably transfecting the GPR120-Rluc gene were seeded in two black 96-well plates (100 μl, 2 × 10^4^ cells per well). After incubating the cells for 24 h, the medium of the non-specific plate was removed, and positive control GSK134647A (15 μM, 50 μl per well) was added into each well. After a 40-min incubation, the medium was removed, and probe N1 was added into two plates (100 μl per well). Followed by incubation for 40 min, coelenterazine was added to the plates (final concentration: 10 μM, 100 μl per well for specific plates; 50 μl per well for non-specific plates). Finally, the fluorescence intensity was measured at 460 and 520 nm with the microplate reader after a 10-min incubation (Christiansen et al., 2016).

#### 4.2.8 Developing Bioluminescence Resonance Energy Transfer Competitive Experiment

HEK293 cells stably transfected with the GPR120-Rluc gene were seeded in a black 96-well plate (100 μl, 2 × 10^4^ cells per well). After the cells were incubated for 24 h, the medium was removed, and probe N1 (400 nM, 50 μl per well) was added. After a 30-min incubation, positive controls GSK134647A and TUG-891 (100 μl per well) were added. Followed by a 40-min incubation, coelenterazine was added to (final concentration: 10 μM, 50 μl per well) the plates. Thenceforward, the fluorescence intensity was measured at 450 and 550 nm with the microplate reader after a 10-min incubation.

Using the same method, the BRET competition experiment was conducted at different concentrations of probe N1 (200 nM, 500 nM, 1000 nM).

#### 4.2.9 Bioluminescence Resonance Energy Transfer Kinetic Binding Experiment

HEK293 cells stably transfecting the GPR120-Rluc gene were seeded in two entirely black 96-well plates (100 μl, 2 × 10^4^ cells per well). After a 24-h incubation, the medium was removed from the non-specific plate, coelenterazine was added to (final concentration: 10 μM, 50 μl per well) the plates, and BRET value was measured after a 5-min incubation. Probe N1 (500 nM, 100 μl per well) was then added, and the BRET value was recorded in a 30-s interval for 6 minutes. Finally, GSK137647A was added (50 μl per well), and BRET value was measured in a 30-s interval for 30 min.

#### 4.2.10 The Activity Experiments of Agonists

HEK293 cells stably transfected with the GPR120-Rluc gene were seeded in a black 96-well plate (100 μl, 2 × 10^4^ cells per well). After a 24-h incubation, the medium was removed, probe N1 (400 nM, 50 μl per well) was added, and 10 μM of the compound was added after a 30-min incubation as well. Followed by a 40-min incubation, coelenterazine (final concentration: 10 μM, 50 μl per well) was added to the plate. Afterward, the fluorescence intensity was measured at 450 and 550 nm with the microplate reader after another 10-min incubation.

#### 4.2.11 The Bias of Agonists

The bias was quantified by Eq. 1, in which β is the bias factor, E_max_ is the maximum effect produced by the agonist, and Pl and P2 represents the G_q_ and β-arrestin2 signal pathway, respectively.

Formula 1:

$$\beta =\log\left({\left(\frac{E_{\max,P1}}{EC_{50,P1}}\frac{EC_{50,P2}}{E_{\max,P2}}\right)}_{lig}\times {\left(\frac{E_{\max,P2}}{EC_{50,P2}}\frac{EC_{50,P1}}{E_{\max,P1}}\right)}_{ref}\right)$$ (1)

#### 4.2.12 The Blood Glucose Experiments

All animal studies were approved by the Ethics Committee and IACUC of Cheeloo College of Medicine, Shandong University, and were conducted in compliance with European Guidelines for the Care and Use of Laboratory Animals. After adaptive feeding for 10–15 days, the db/db mice were divided into positive control group (30 mg/kg), low content compound group (10 mg/kg), high content compound group (30 mg/kg), and negative control group (the same volume dosing solvent). These mice were administrated by gavage once a day (administration volume, 20 ml/kg) for four consecutive weeks. During the administration period, the fasting blood glucose (without food and water for 6 h) was measured twice a week by using a blood glucose meter. The blood glucose level was measured for four consecutive weeks. The data was processed with Graphpad 8.2 software (Cox et al., 2017; Zhang et al., 2017).

#### 4.2.13 Glucose Tolerance Test

On the last day of the dosing cycle, the fasting blood glucose of mice was measured (without food and water for 6 h), and glucose solution (100 mg/kg) was injected. The blood glucose was measured at six-time points (5, 10, 30, 60, and 120 min). Finally, the area under the curve was analyzed with SPSS 24 software.

## Data Availability

The original contributions presented in the study are included in the article/Supplementary Material, further inquiries can be directed to the corresponding author.

## Abbreviations

BRET, bioluminescence resonance energy transfer; Ca^2+^, calcium ion; FFA4, free fatty acid receptor 4; GPCRs, G protein-coupled receptors; GPR40, G protein-coupled receptor 40; GPR120, G protein-coupled receptor 120; Rluc, Renilla luciferase.
