# Supplementary material for: Fluorescent Ligand-Based Discovery of Small-Molecule Sulfonamide Agonists for GPR120
Source: Front Chem. 2022 Jan 31;10:816014. doi: 10.3389/fchem.2022.816014 (PMC8841740; doi:10.3389/fchem.2022.816014)
Supplement: Supplementary file 1 [file DataSheet1.docx]

Supplementary Information

Fluorescent Ligand-based Discovery of Small-molecule Sulfonamide Agonists for GPR120

Siyue Ma^1,＃^, Zhenzhen Li^1,#^, Yueli Yang^1^, Ling Zhang^1^, Minyong Li^1^, Lupei Du^1*^

^1^Department of Medicinal Chemistry, Key Laboratory of Chemical Biology (MOE), School of Pharmacy, Cheeloo College of Medicine, Shandong University, Jinan, Shandong 250012, China

# These authors contributed equally.

**Table of contents**

1. The excitation and emission spectra of fluorescent ligands.............................................................S2

2. The UV-visible absorption spectra of fluorescent ligands **N1-N3**...................................................S4

3. Cell imaging with fluorescent ligands..............................................................................................S4

4. BRET competitive binding experiment............................................................................................S5

5. GPR40 Ca^2+^ assay of probes.............................................................................................................S6

6. The cytotoxicity of probes................................................................................................................S6

7. The weight changes of db/db mice...................................................................................................S7

8. The chemical structure of GPR120 small-molecule agonists...........................................................S8

NMR, MS and HRMS data of compounds...........................................................................................S9

HPLC data of final compounds..........................................................................................................S41

1. **The excitation and emission spectra of fluorescent ligand**


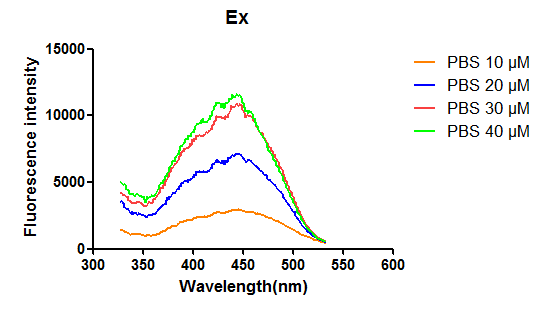

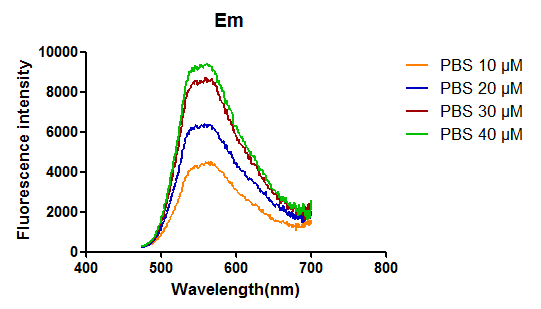

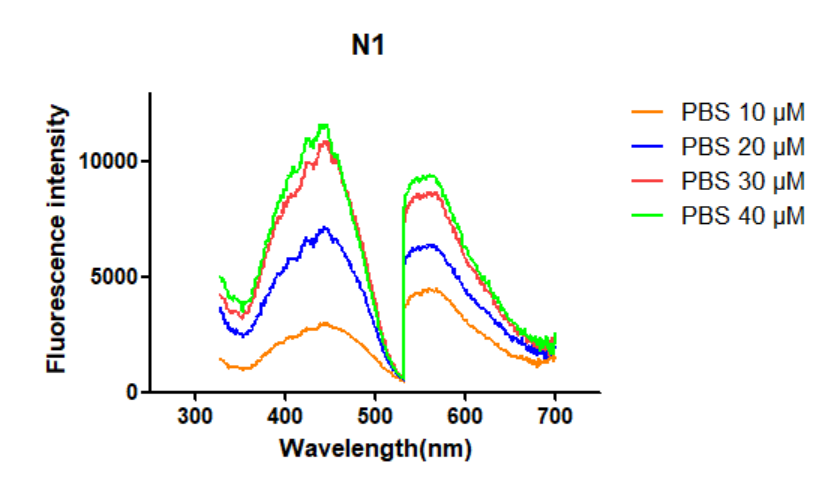


**Figure S1.** The excitation (444 nm) and emission (562 nm) spectra of fluorescent probe **N1** (10 μM, 20 μM, 30 μM and 40 μM) in PBS.


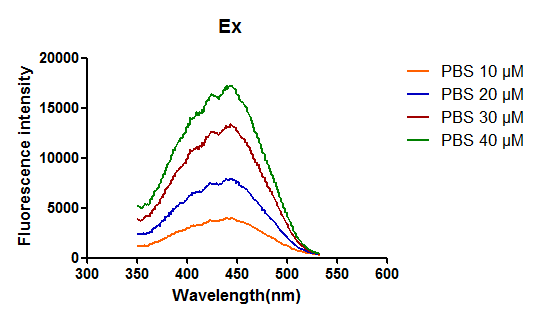

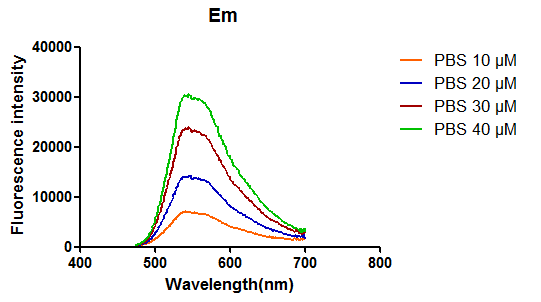

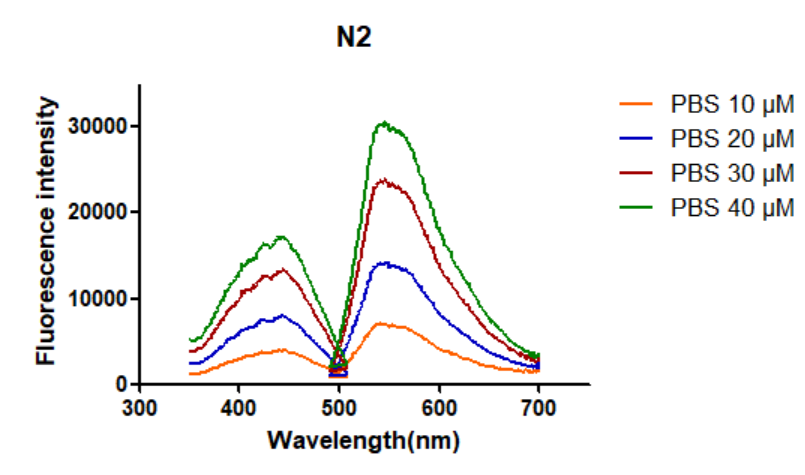


**Figure S2.** The excitation (439 nm) and emission (562 nm) spectra of fluorescent probe **N2** (10 μM, 20 μM, 30 μM, 40 μM) in PBS.


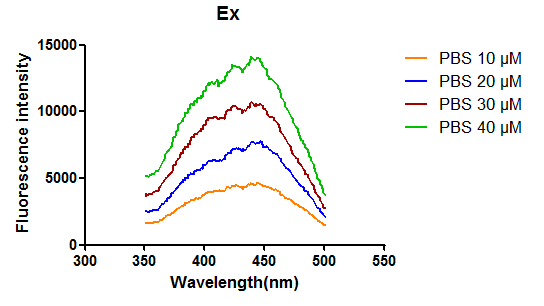

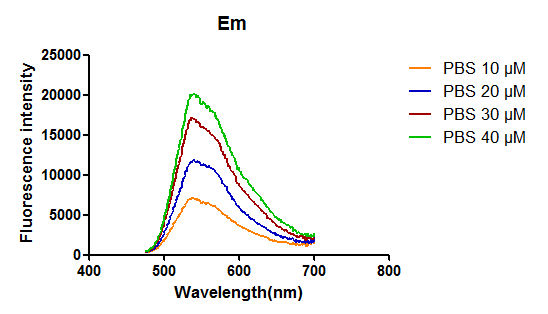

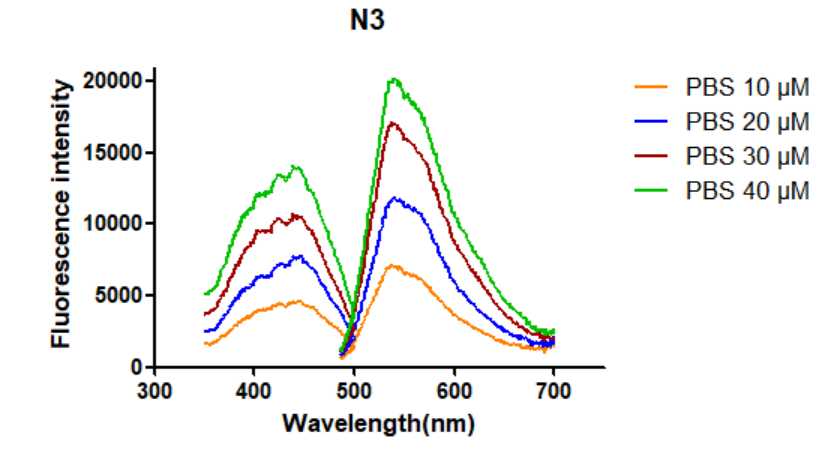


**Figure S3.** The excitation (438 nm) and emission (560 nm) spectra of fluorescent probe **N3** (10 μM, 20 μM, 30 μM, 40 μM) in PBS.


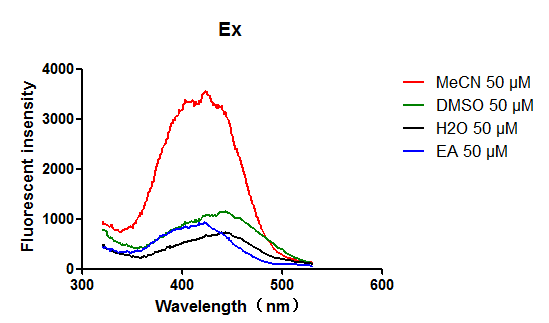

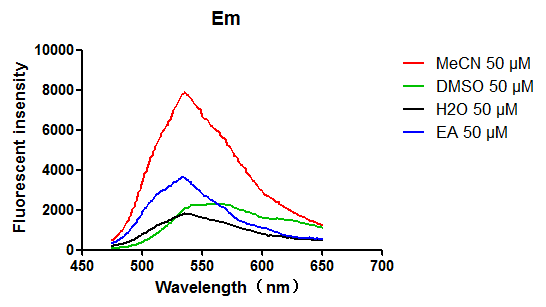


**Figure S4.** The excitation (444 nm) and emission (562 nm) spectra of fluorescent probe ***N1*** (50 μM) in MeCN, DMSO, H_2_O, EA.


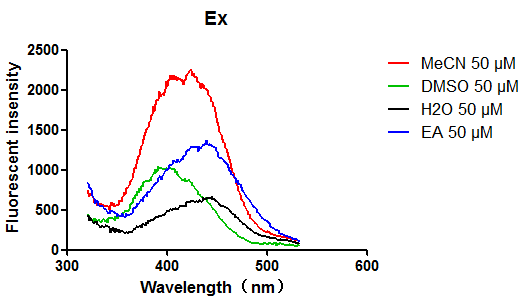

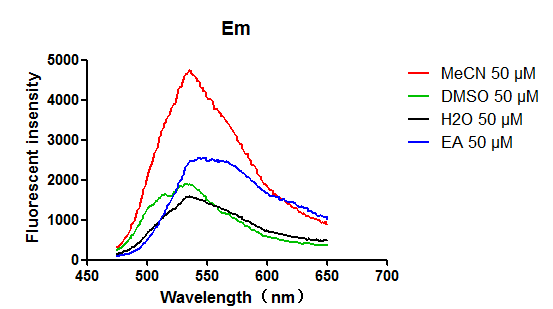


**Figure S5.** The excitation (439 nm) and emission (562 nm) spectra of fluorescent probe ***N2*** (50 μM) in MeCN, DMSO, H_2_O, EA.


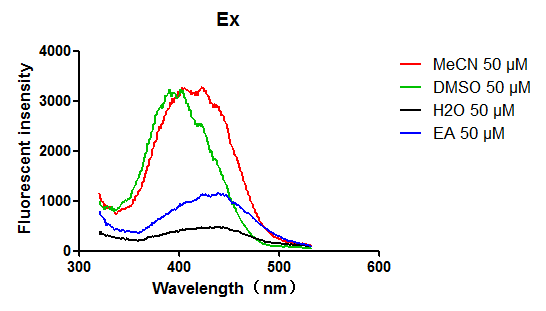

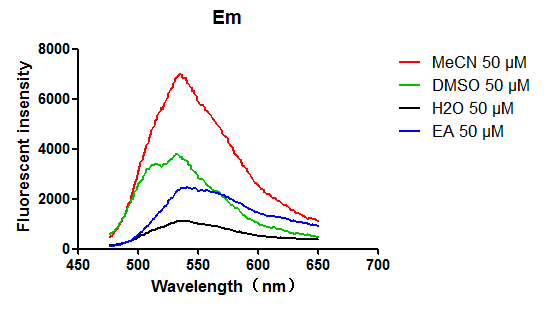


**Figure S6.** The excitation (438 nm) and emission (560 nm) spectra of fluorescent probe ***N3*** (50 μM) in MeCN, DMSO, H_2_O, EA.

1. **The UV-visible absorption spectra of fluorescent ligands *N1-N3***


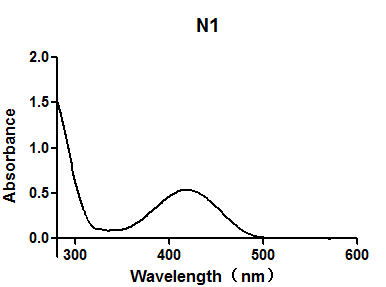

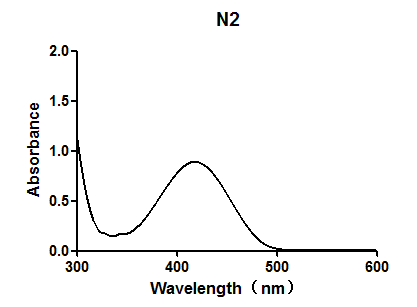

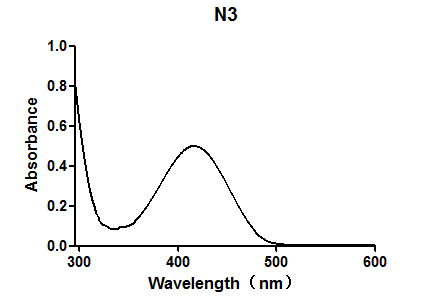


**Figure S7.** The UV-visible absorption spectra of fluorescent ligands ***N1-N3*** in MeCN.

1. **Cell imaging with fluorescent ligands**


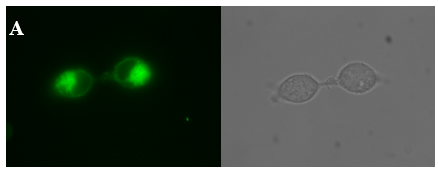

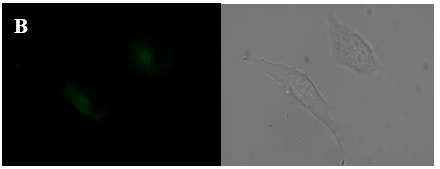


**Figure S8.** Probe ***N1*** was exposed for 500 ms and performed in the GFP channel. 63x lens magnification. (A) Probe ***N1*** was 0.5 μM in HEK293 cells. (B) Probe ***N1*** was 0.5 μM in PC-3 cells.


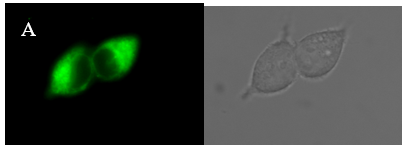

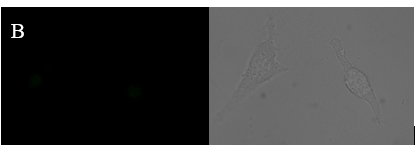


**Figure S9.** Probe ***N2*** was exposed for 500 ms and performed in the GFP channel. 63x lens magnification. (A) Probe ***N2*** was 5 μM in HEK293 cells. (B) Probe ***N2*** was 5 μM in PC-3 cells.


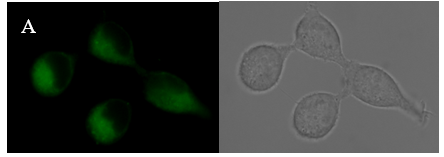

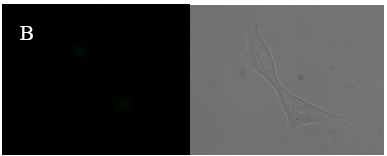


**Figure S10.** Probe ***N3*** was exposed for 500 ms and performed in the GFP channel. 63x lens magnification. (A) Probe ***N3*** was 5 μM in HEK293 cells. (B) Probe ***N3*** was 5 μM in PC-3 cells.

1. **BRET competitive binding experiment**


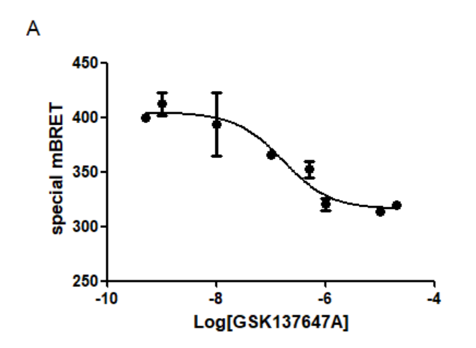

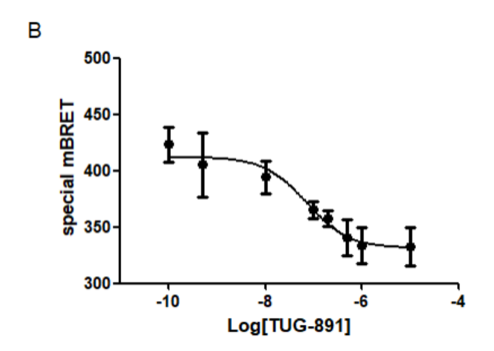


**Figure S11.** (A) Competitive binding of probe ***N1*** (400 nM) to the agonist GSK134647A. (B) Competitive binding of probe ***N1*** (400 nM) to the agonist TUG-891.

**
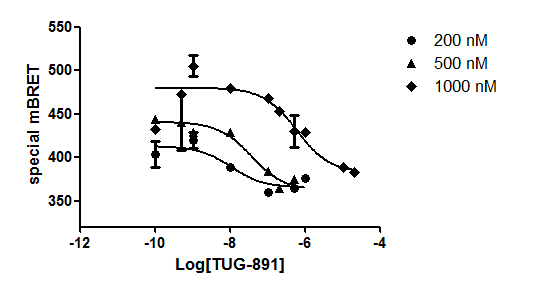
**

**Figure S12.** Competitive binding of different concentrations of probe ***N1*** to the positive drug TUG-891.

1. **GPR40 Ca^2+^ assay of probes**


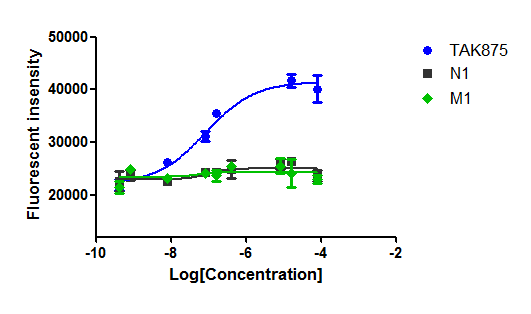


**Figure S13.** The Ca^2+^ response of probes ***N1***, TAK875 against GPR40. TAK875 is a positive drug against GPR40 reported in the literature.

1. **The cytotoxicity of probes**


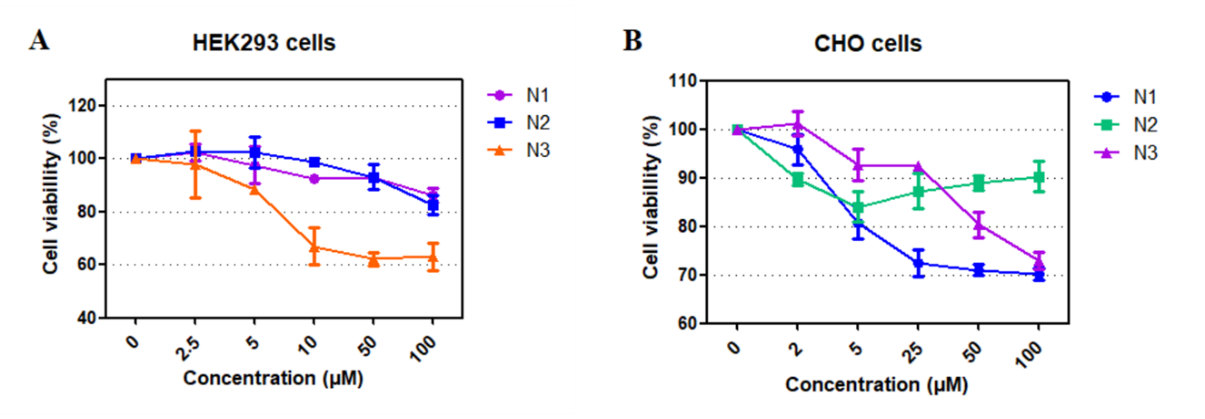

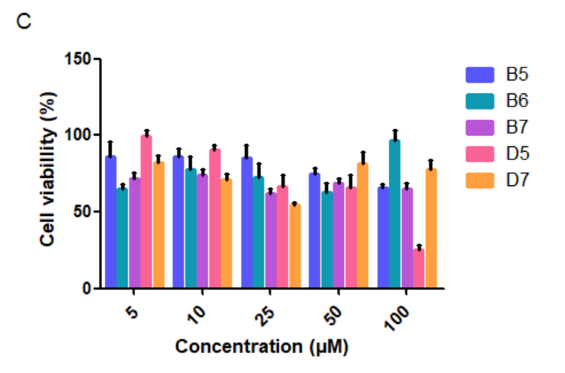

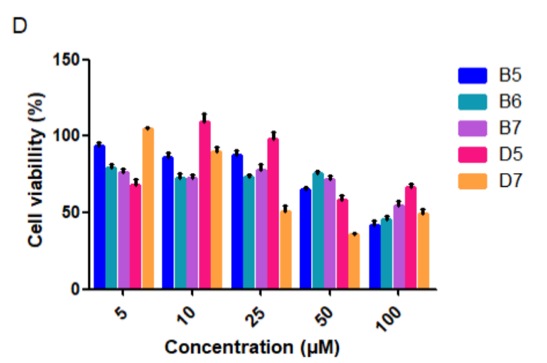


**Figure S14.** (A) (B) Cell viability (%) of probes ***N1-N3*** in HEK293 cells and CHO cells after 24 h. (C) (D) Cell viability (%) of modified compounds (**B5, B6, B7, D5, D7**) in HEK293 cells and CHO cells after 24 h.

1. **The weight changes of db/db mice**


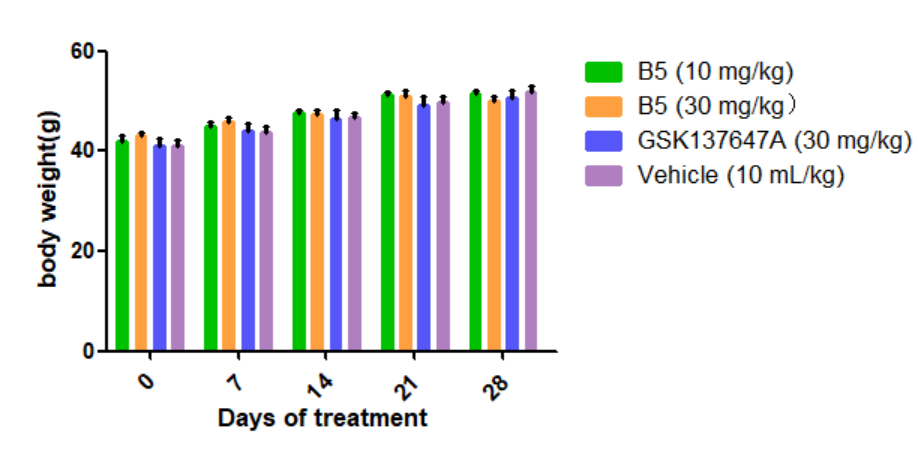


**Figure S15.** The weight changes of db/db mice after four weeks of continuous administration.

1. **The chemical structure of GPR120 small-molecule agonists**

**Table S1.** **The chemical structure of GPR120 small-molecule agonists**

| **Compd** | **Structure** | **Compd** | **Structure** |
| --- | --- | --- | --- |
| **A1** |  | **B1** |  |
| **B2** |  | **B3** |  |
| **B4** |  | **B5** |  |
| **B6** |  | **B7** |  |
| **B8** |  | **C1** |  |
| **C2** |  | **C3** |  |
| **D1** |  | **D2** |  |
| **D3** |  | **D4** |  |
| **D5** |  | **D6** |  |
| **D7** |  |  |  |

**NMR, MS and HRMS data of compounds**


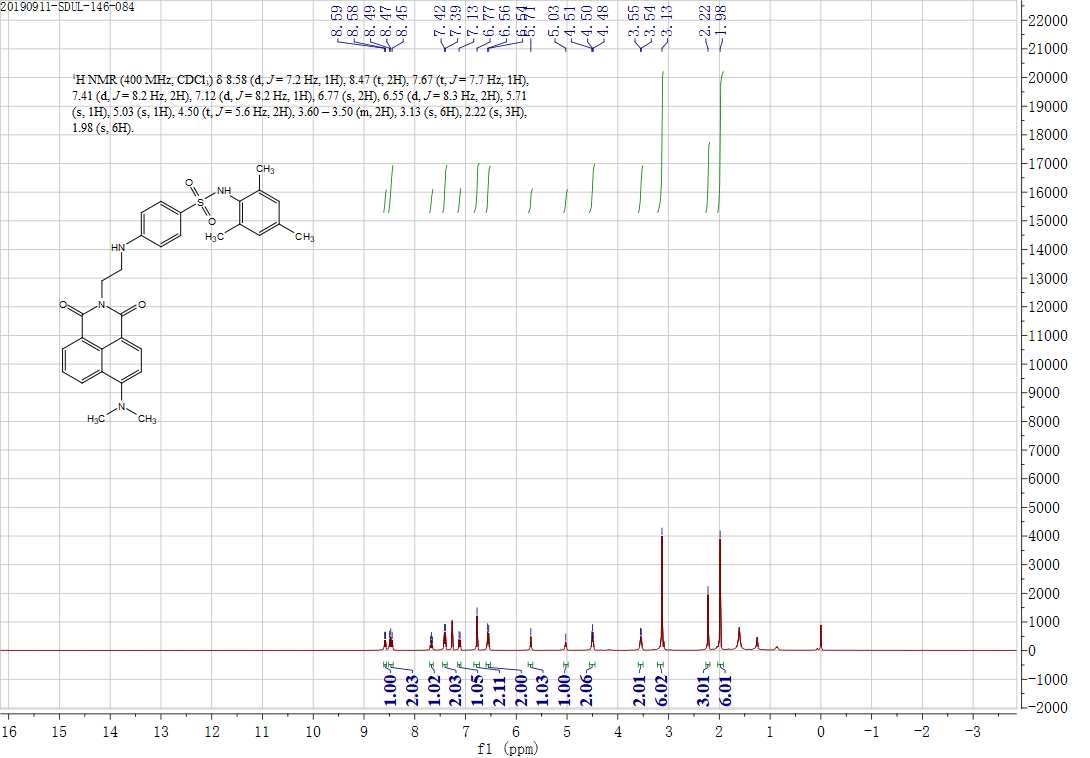


^1^H-NMR spectrum of compound **N1**.


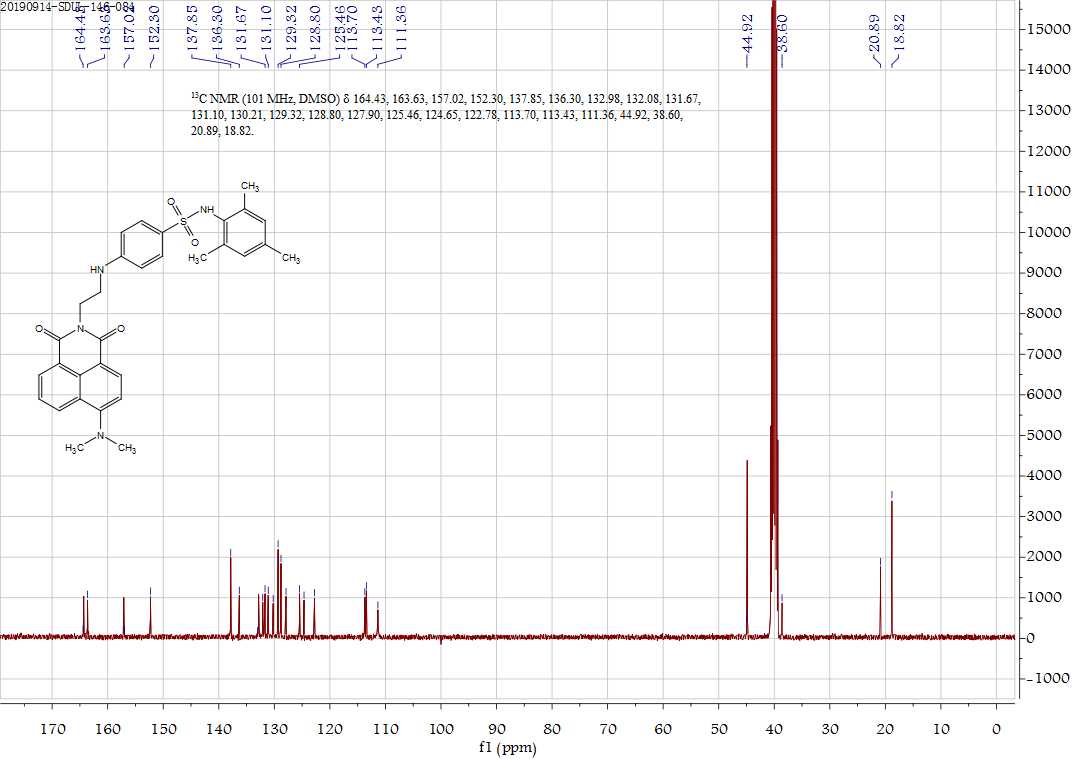


^13^C-NMR spectrum of compound **N1**.


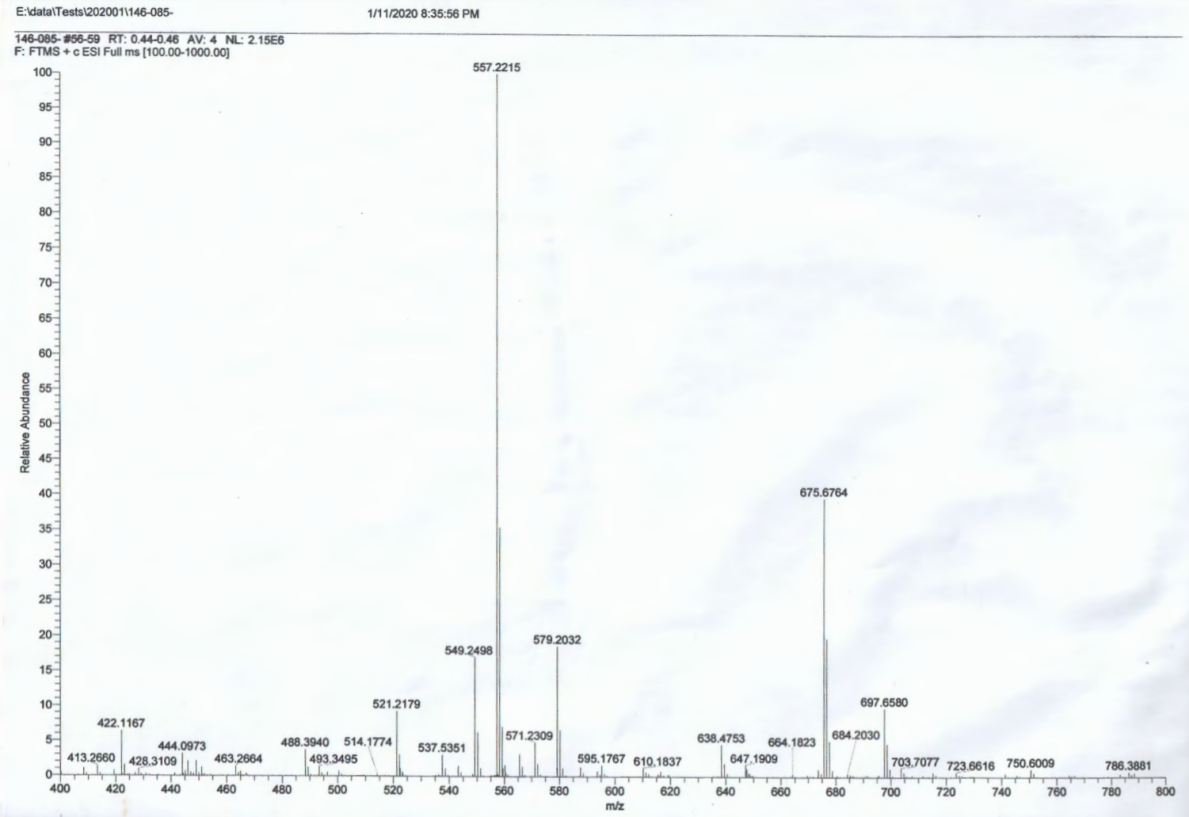


HRMS spectrum of compound **N1**.


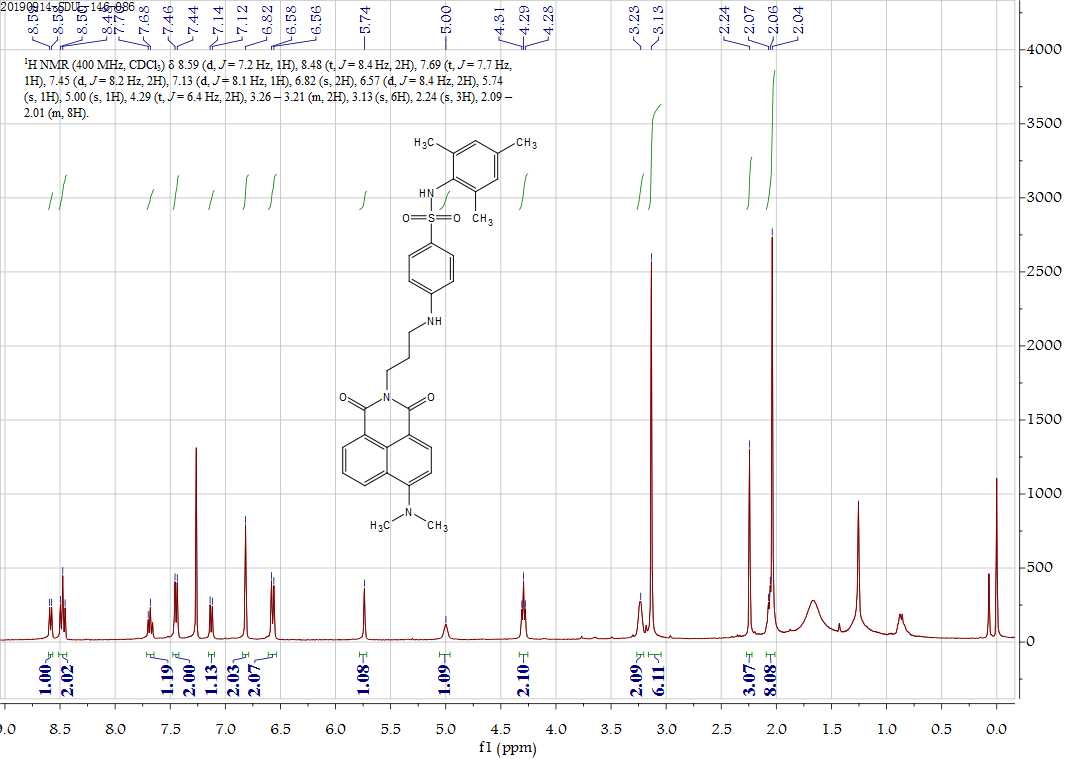


^1^H-NMR spectrum of compound **N2**.


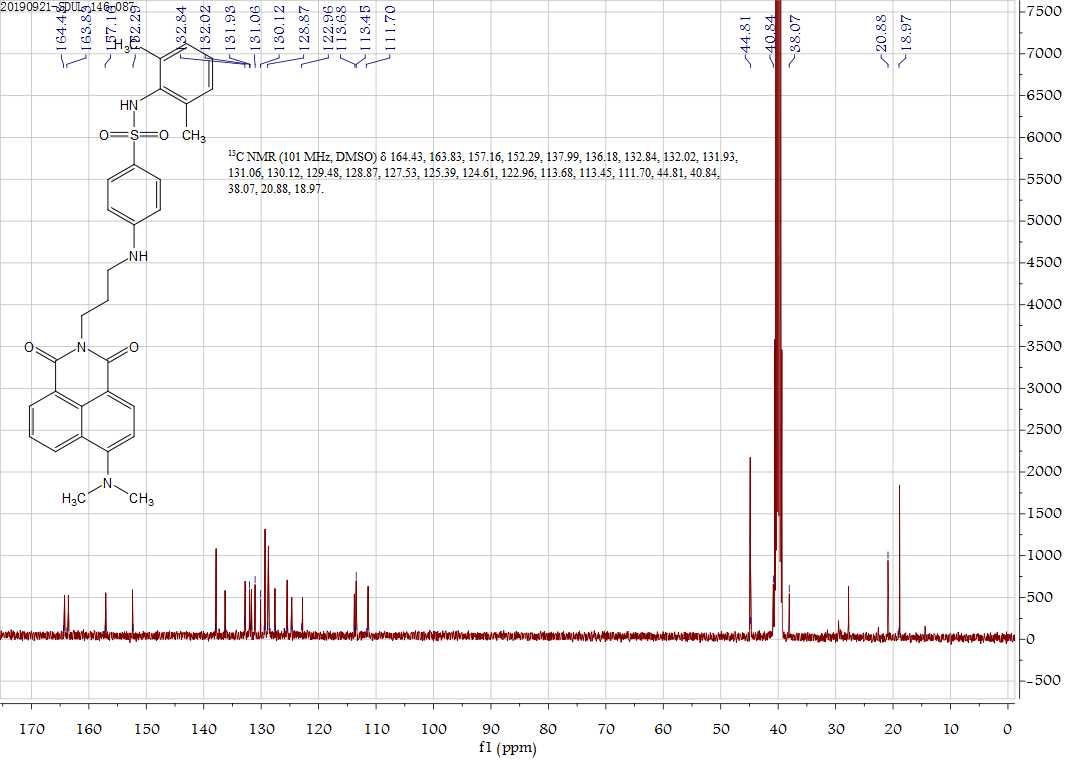


^13^C-NMR spectrum of compound **N2**.


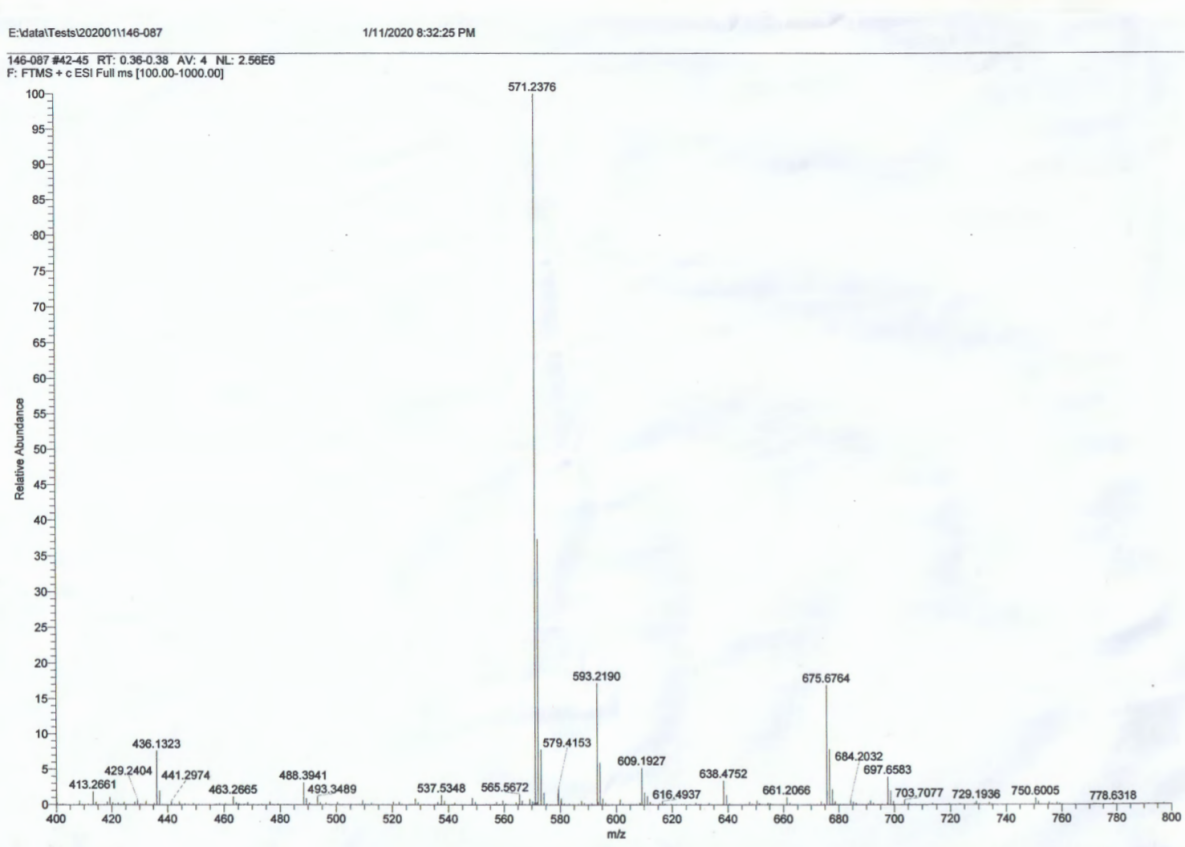


HRMS spectrum of compound **N2**.


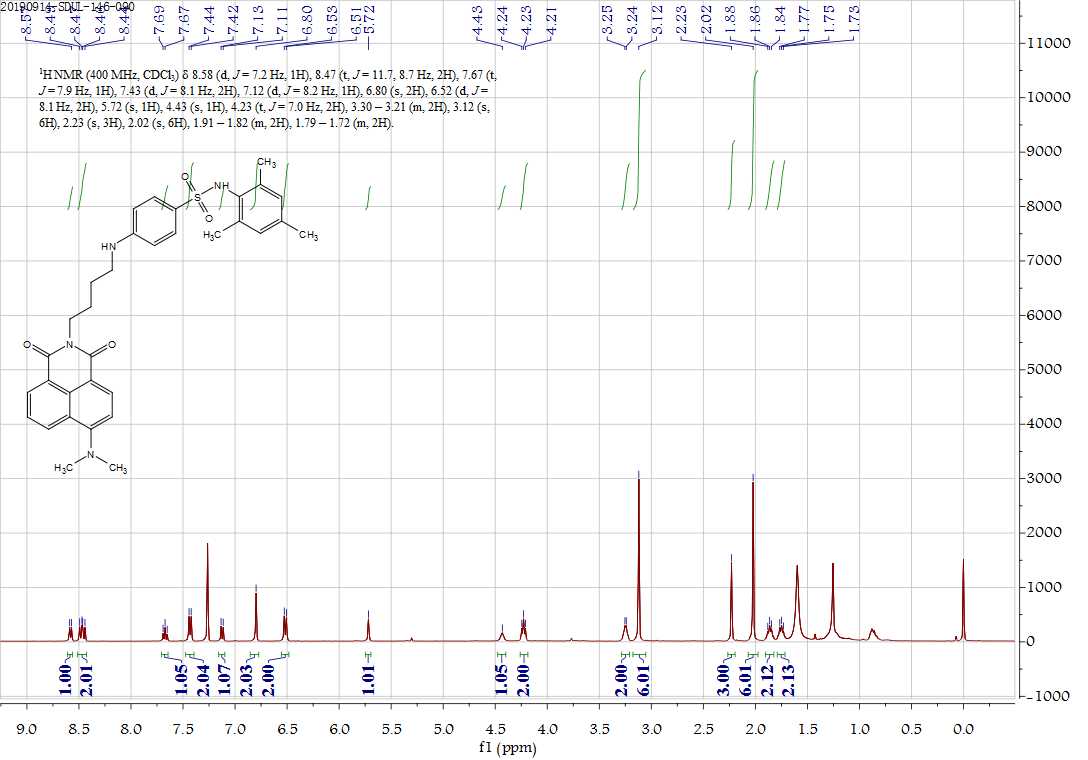


^1^H-NMR spectrum of compound **N3**.


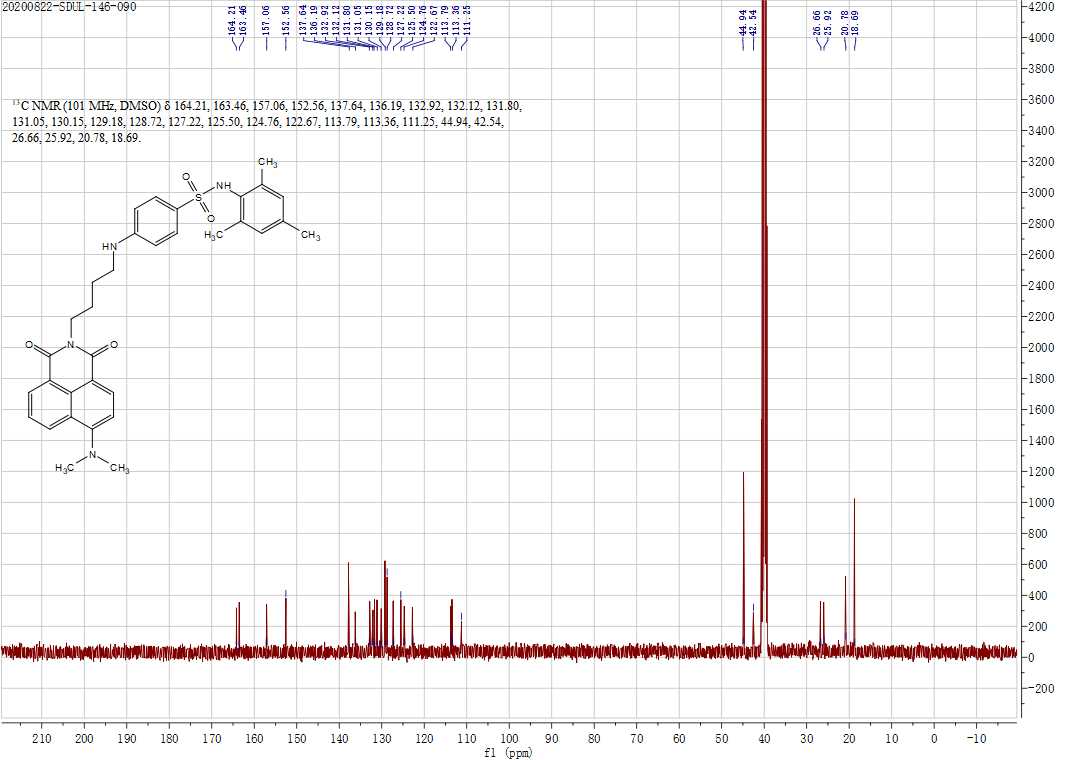


^13^C-NMR spectrum of compound **N3**.


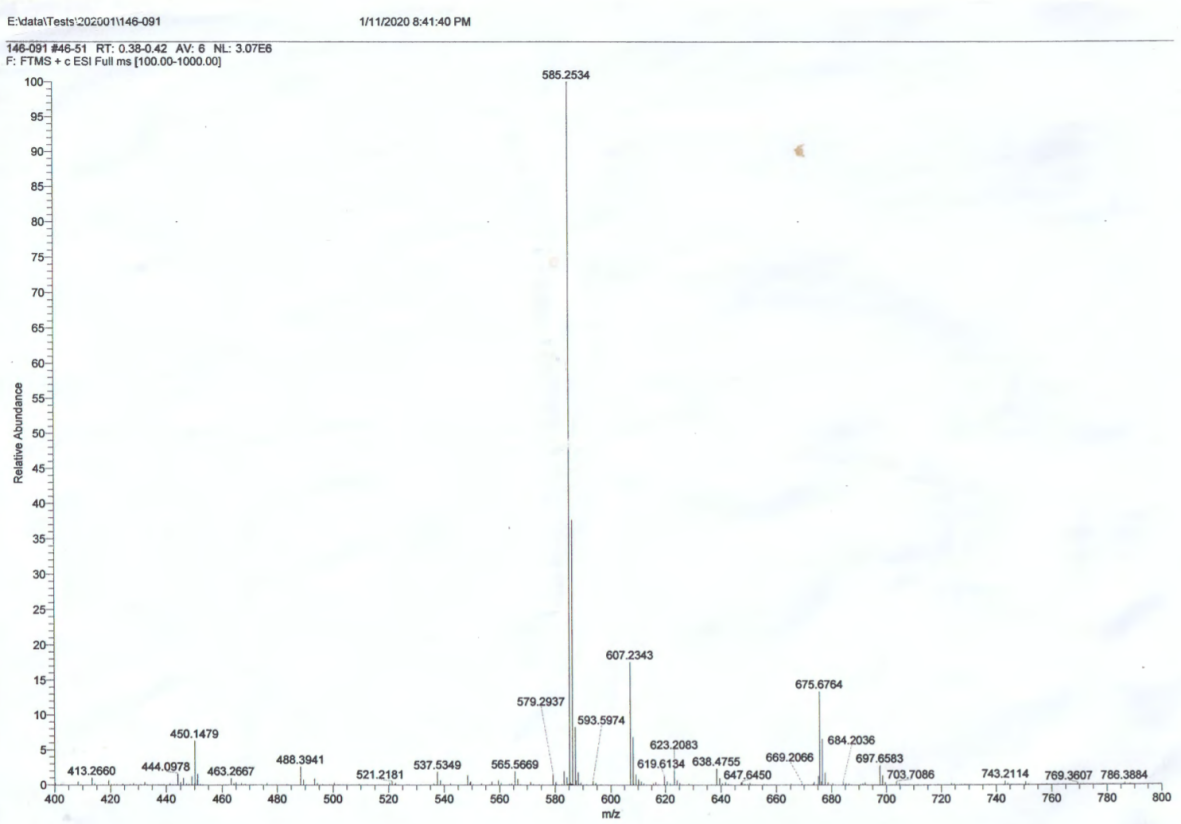


HRMS spectrum of compound **N3**.


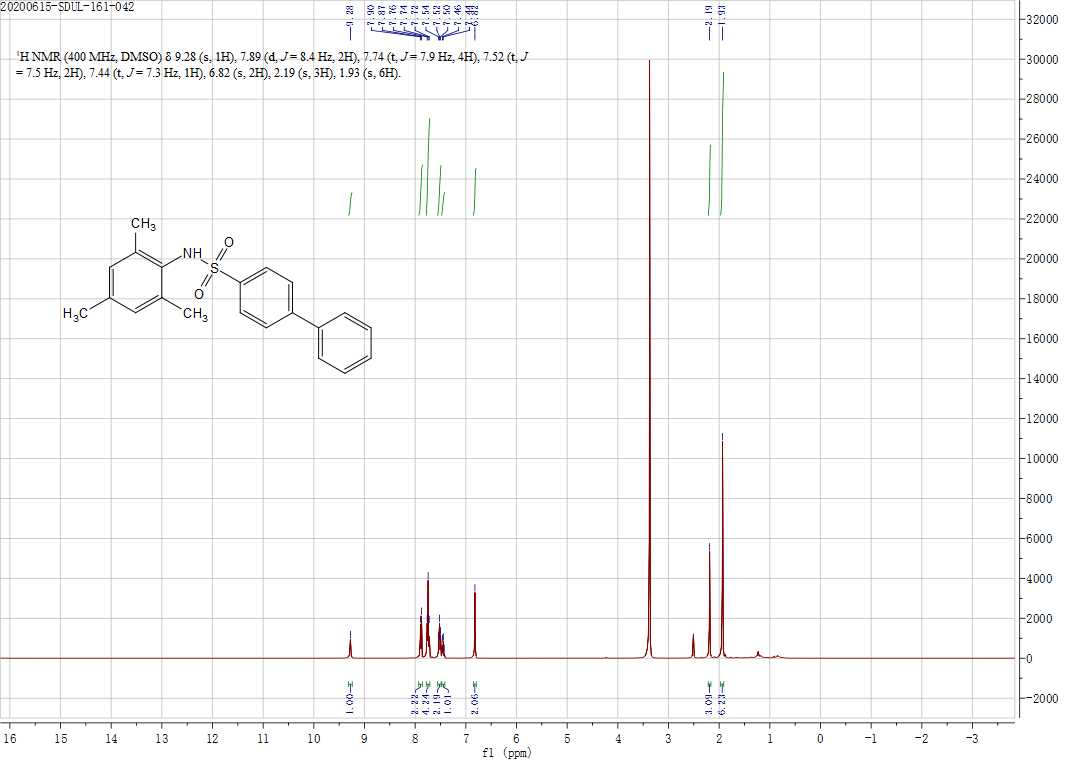


^1^H-NMR spectrum of compound **B1**.

**
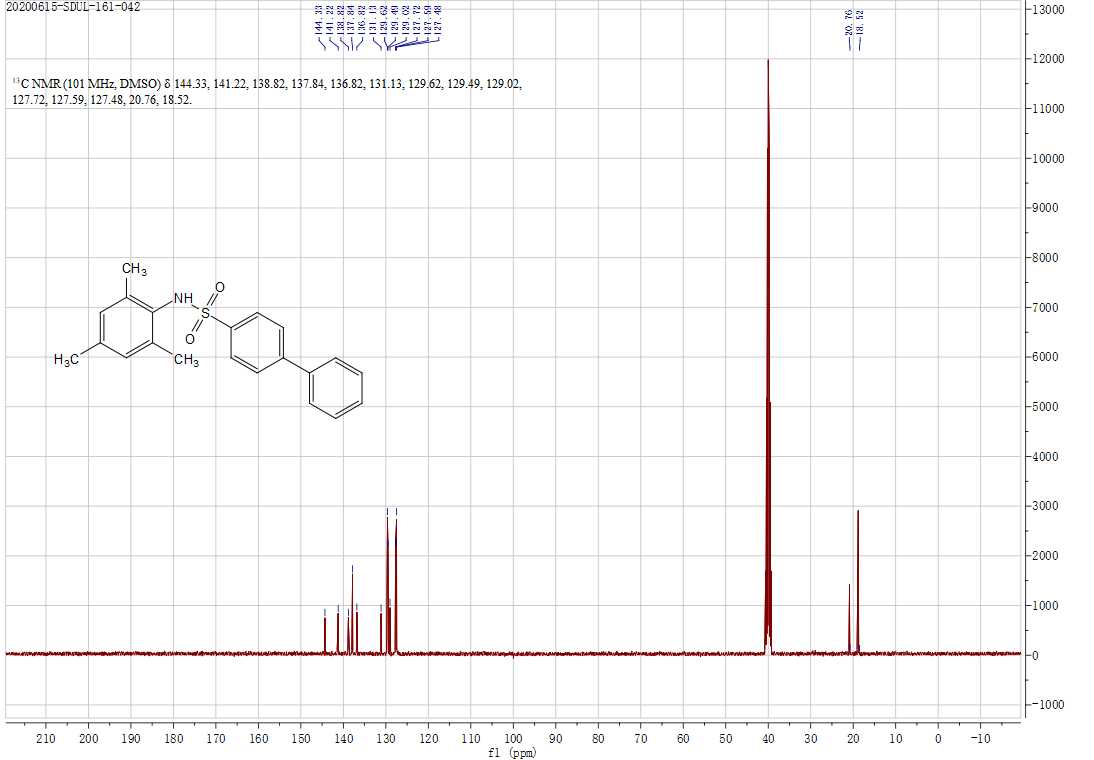
**

^13^C-NMR spectrum of compound **B1**.

HRMS spectrum of compound **B1**.


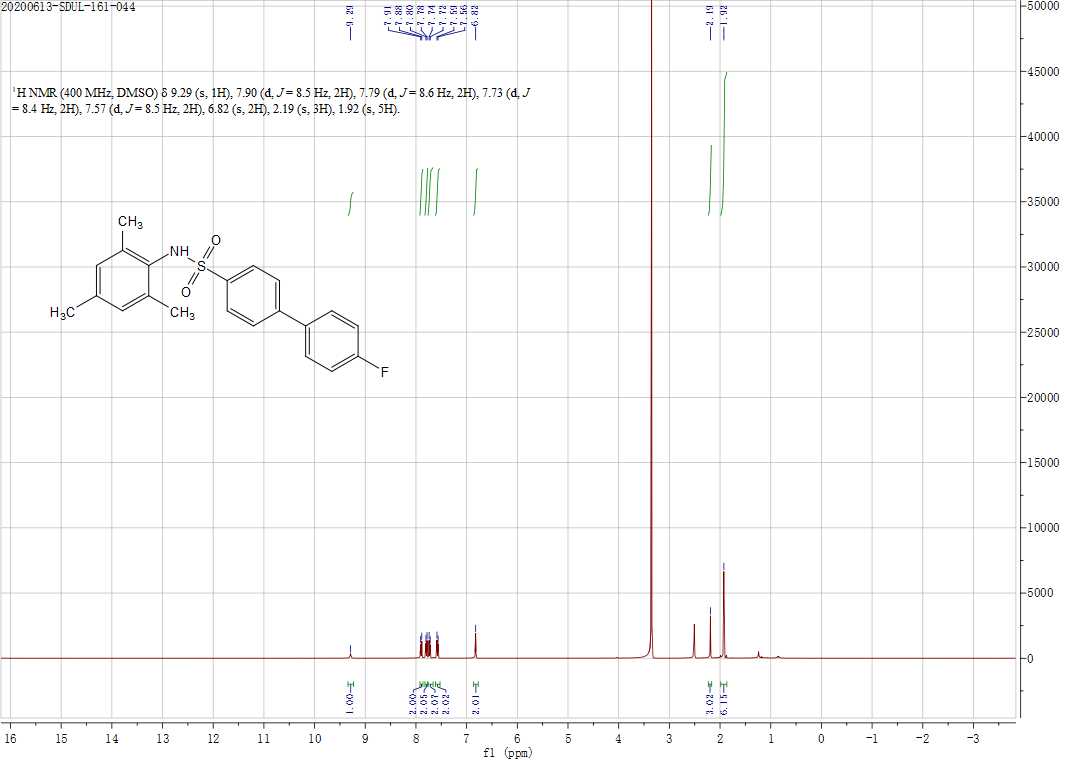


^1^H-NMR spectrum of compound **B2**.


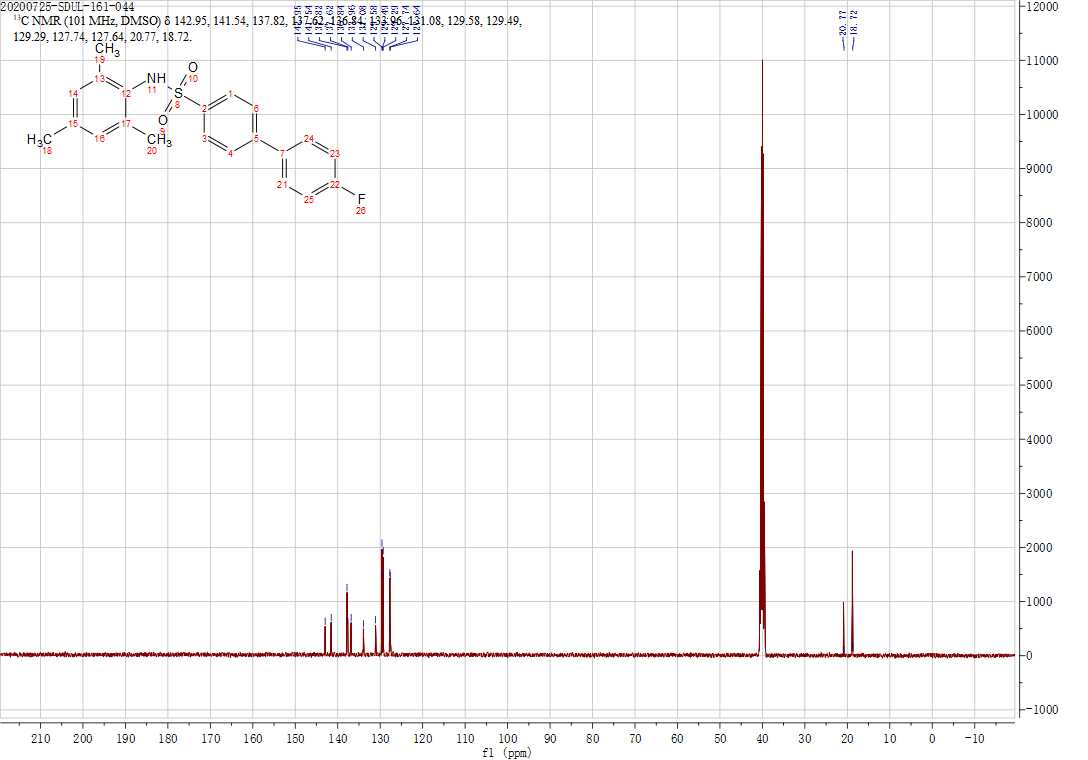


^13^C-NMR spectrum of compound **B2**.

HRMS spectrum of compound **B1**.


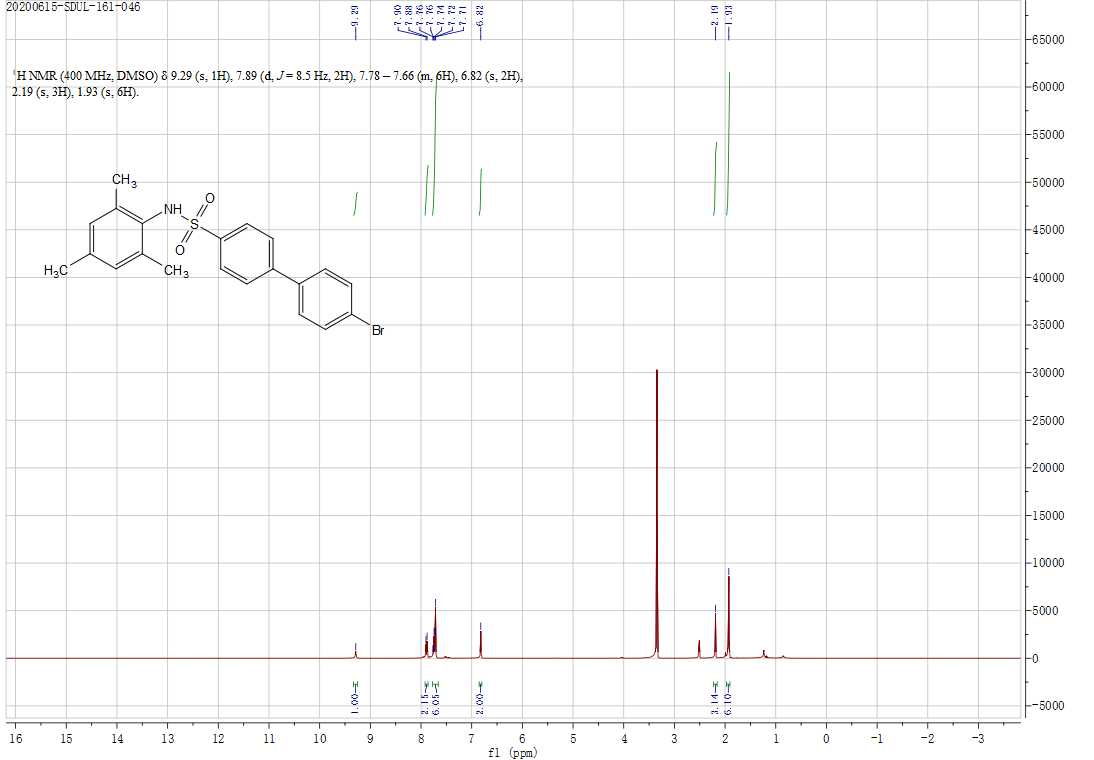


^1^H-NMR spectrum of compound **B3**.

**
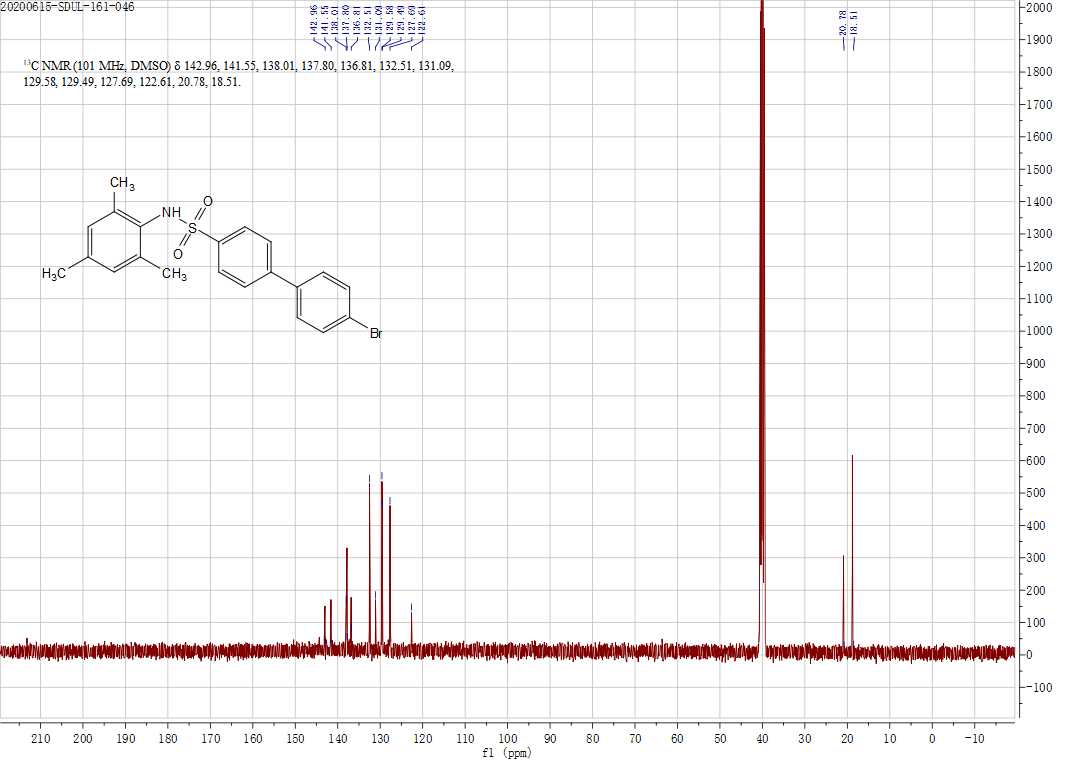
**

^13^C-NMR spectrum of compound **B3**.

HRMS spectrum of compound **B3**.

**
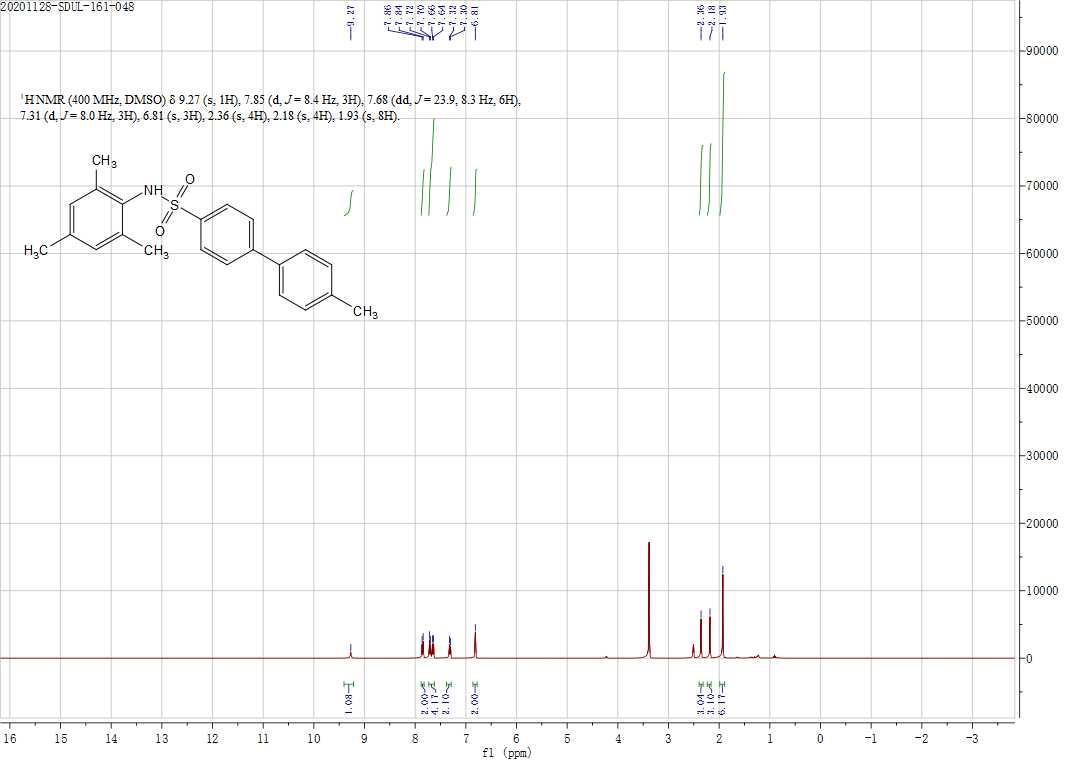
**

^1^H-NMR spectrum of compound **B4**.


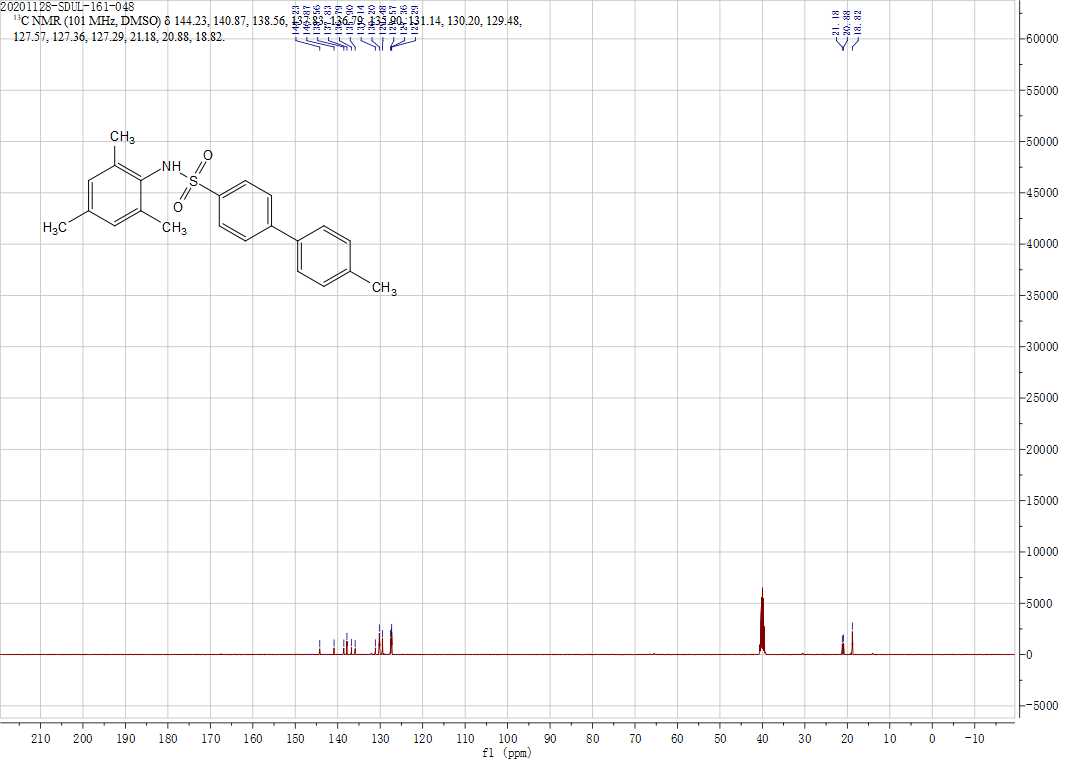


^13^C-NMR spectrum of compound **B4**.

HRMS spectrum of compound **B4**.

**
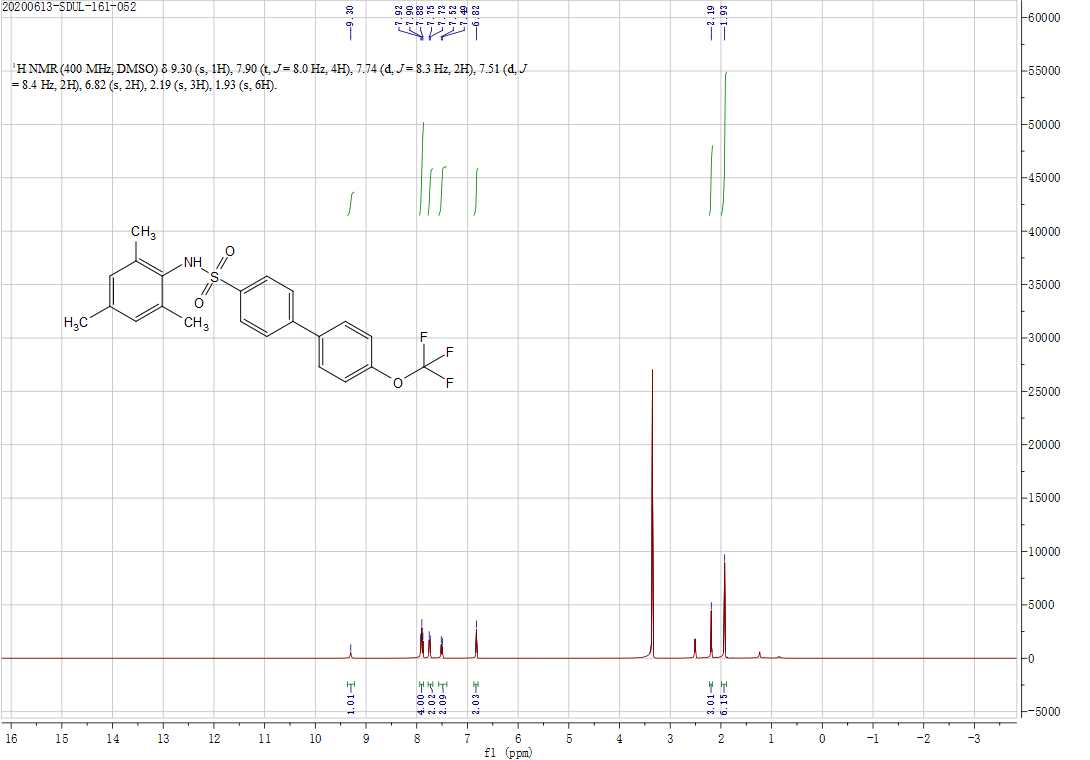
**

^1^H-NMR spectrum of compound **B5**.


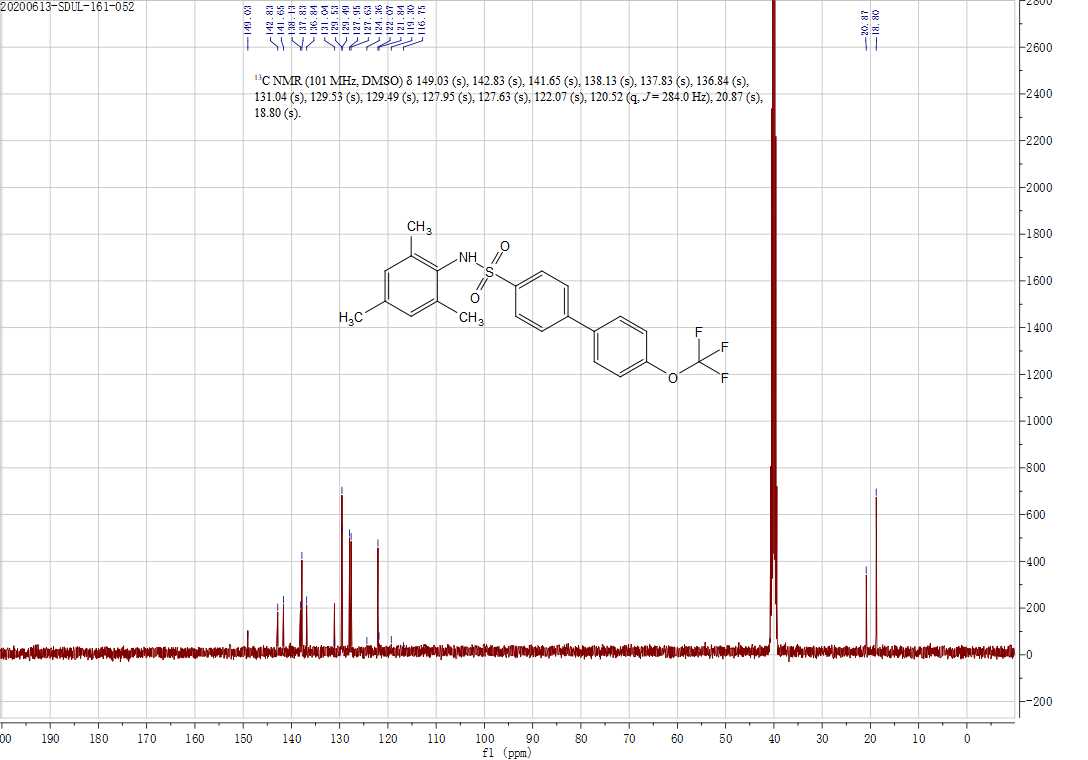


^13^C-NMR spectrum of compound **B5**.

HRMS spectrum of compound **B5**.


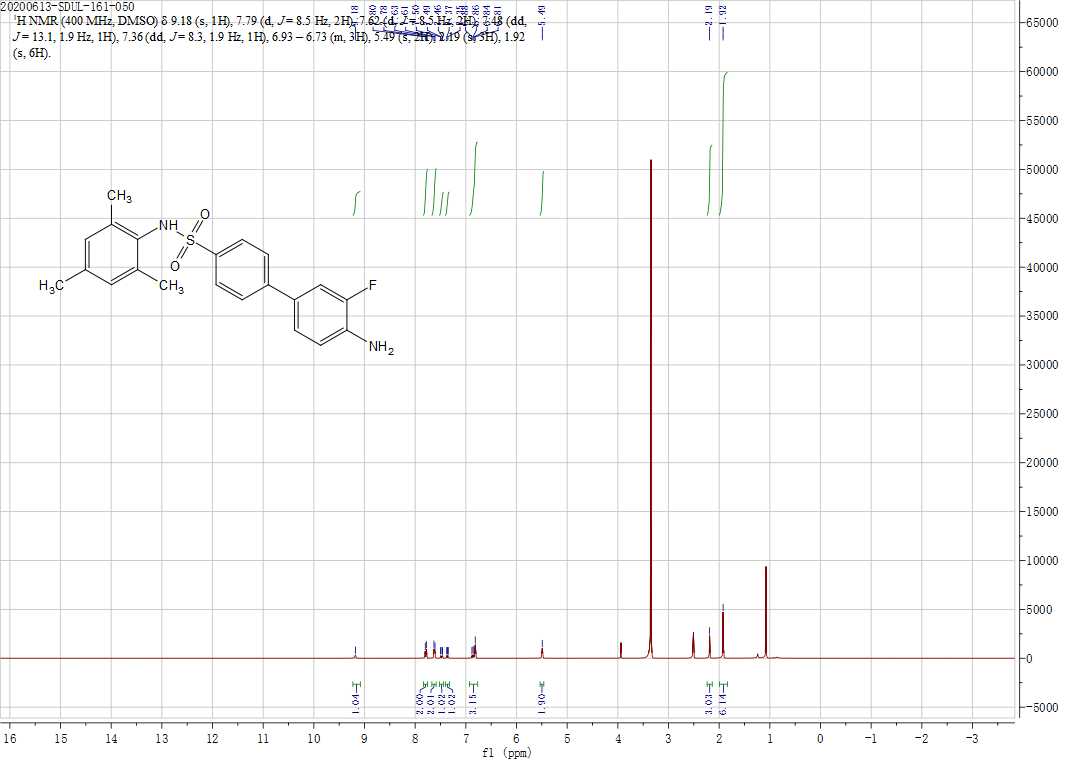


^1^H-NMR spectrum of compound **B6**.


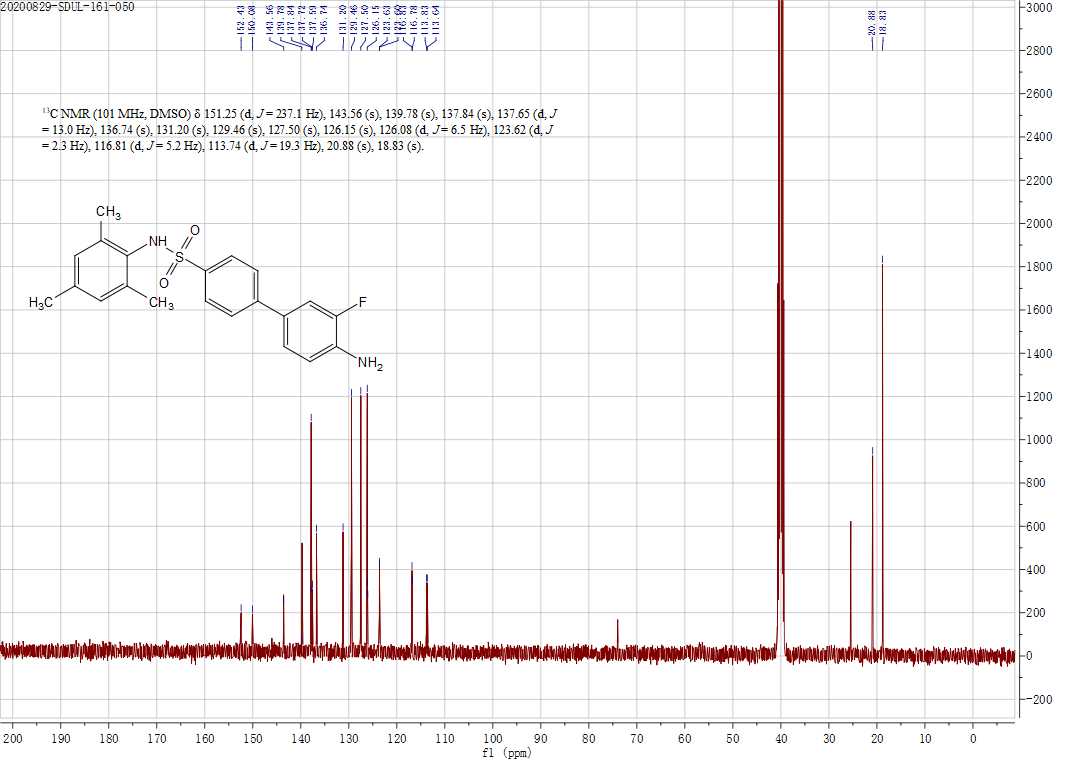


^13^C-NMR spectrum of compound **B6**.

HRMS spectrum of compound **B6.**


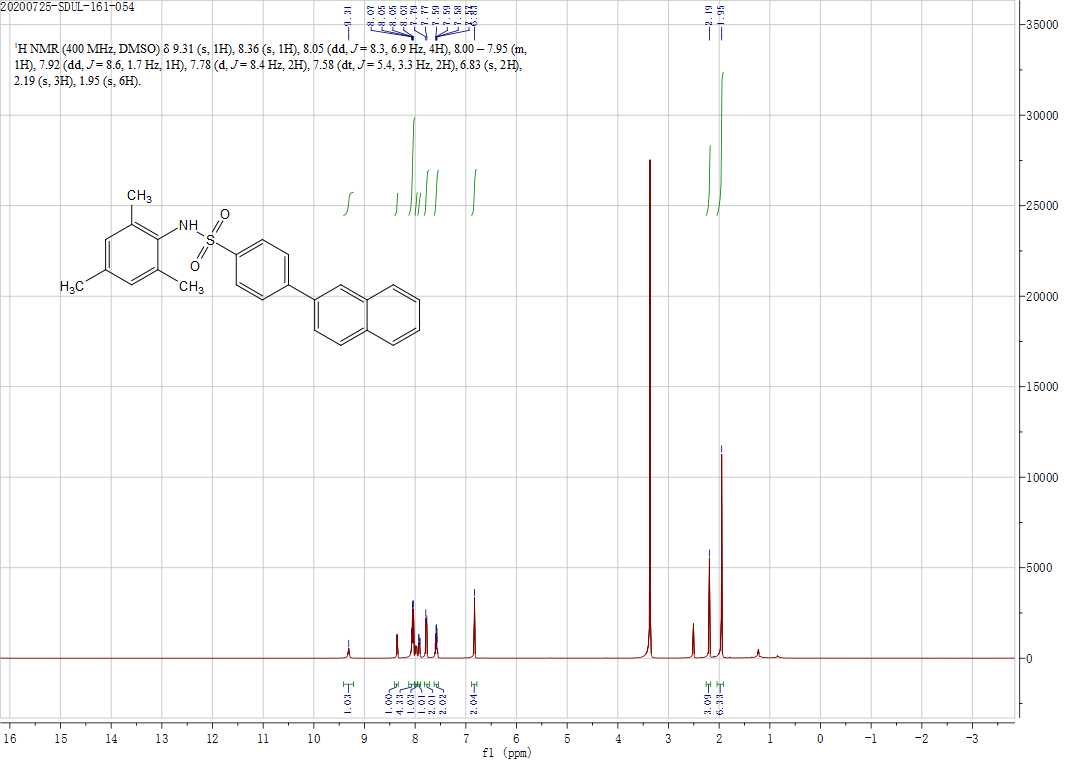


^1^H-NMR spectrum of compound **B7**.


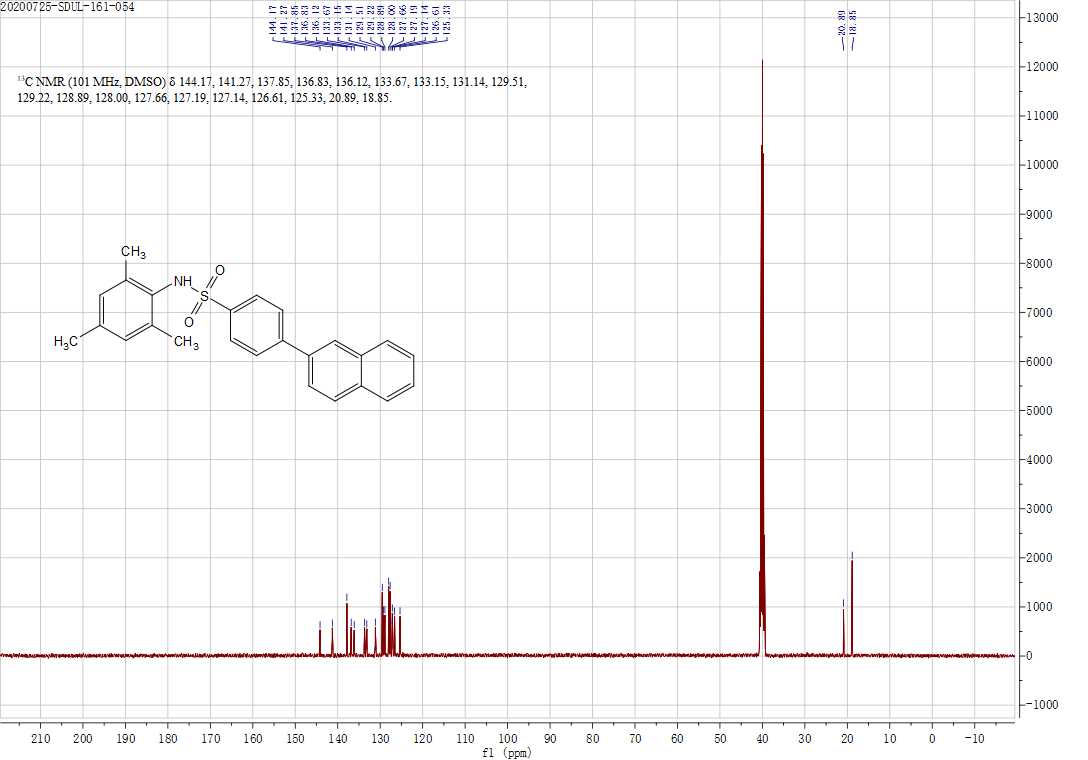


^13^C-NMR spectrum of compound **B7**.

HRMS spectrum of compound **B7.**

**
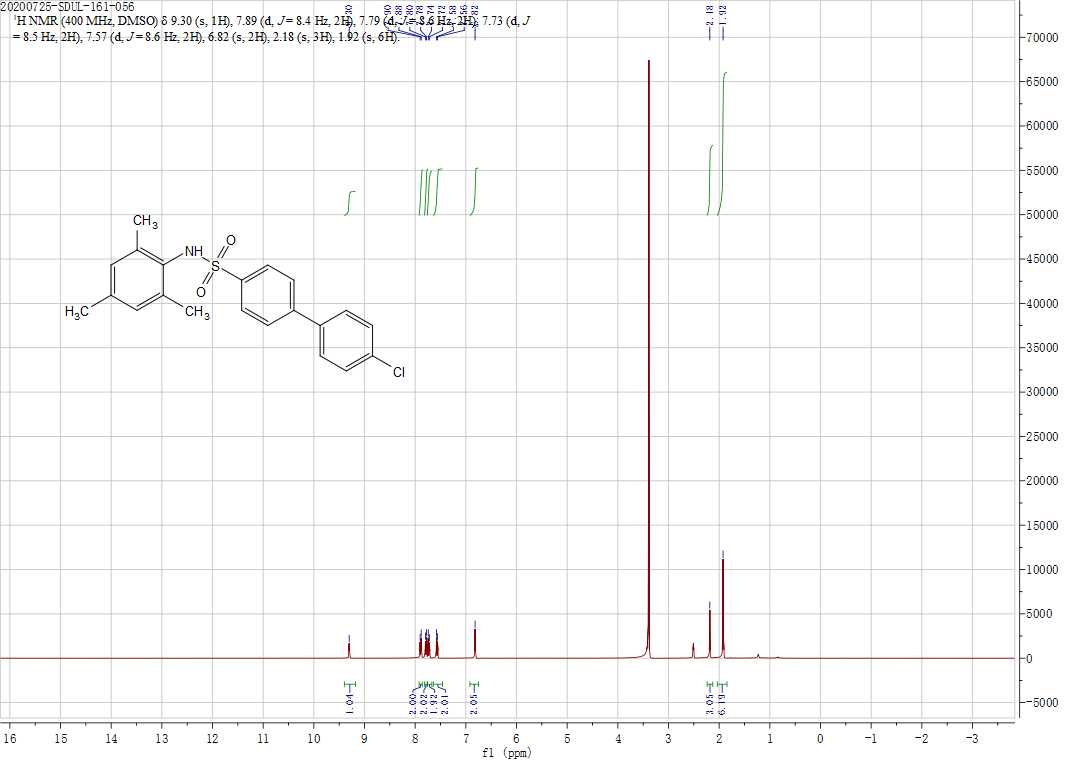
**

^1^H-NMR spectrum of compound **B8**.

**
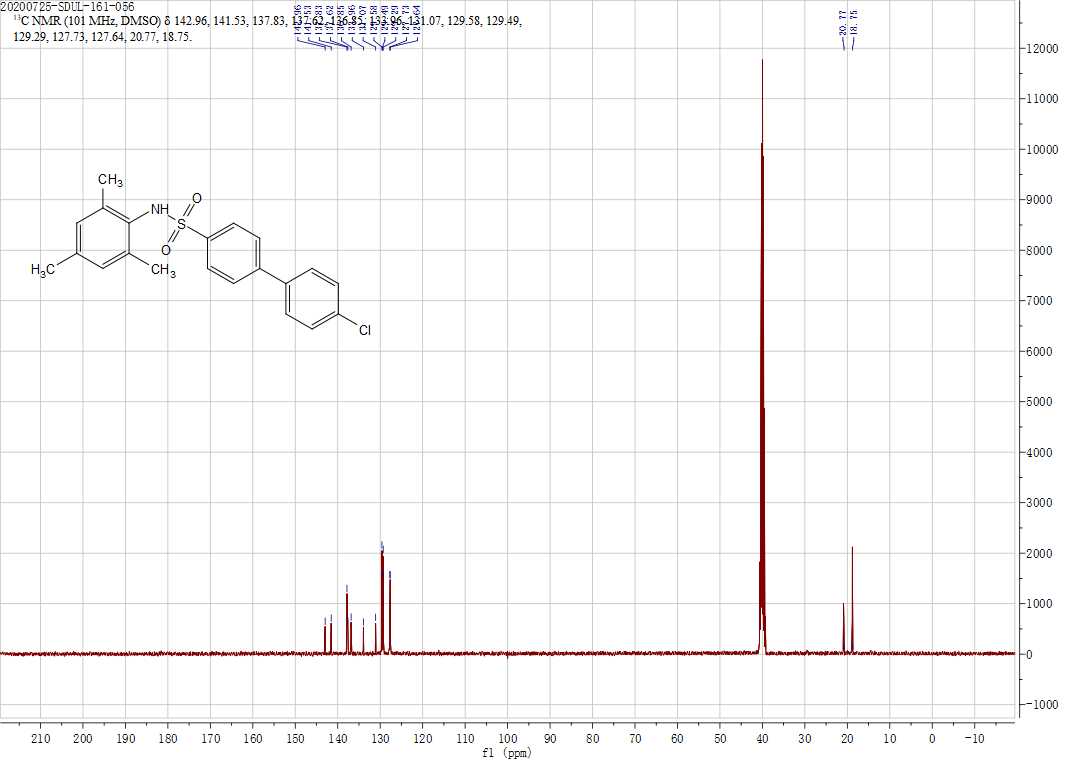
**

^13^C-NMR spectrum of compound **B8**.

HRMS spectrum of compound **B8.**

**
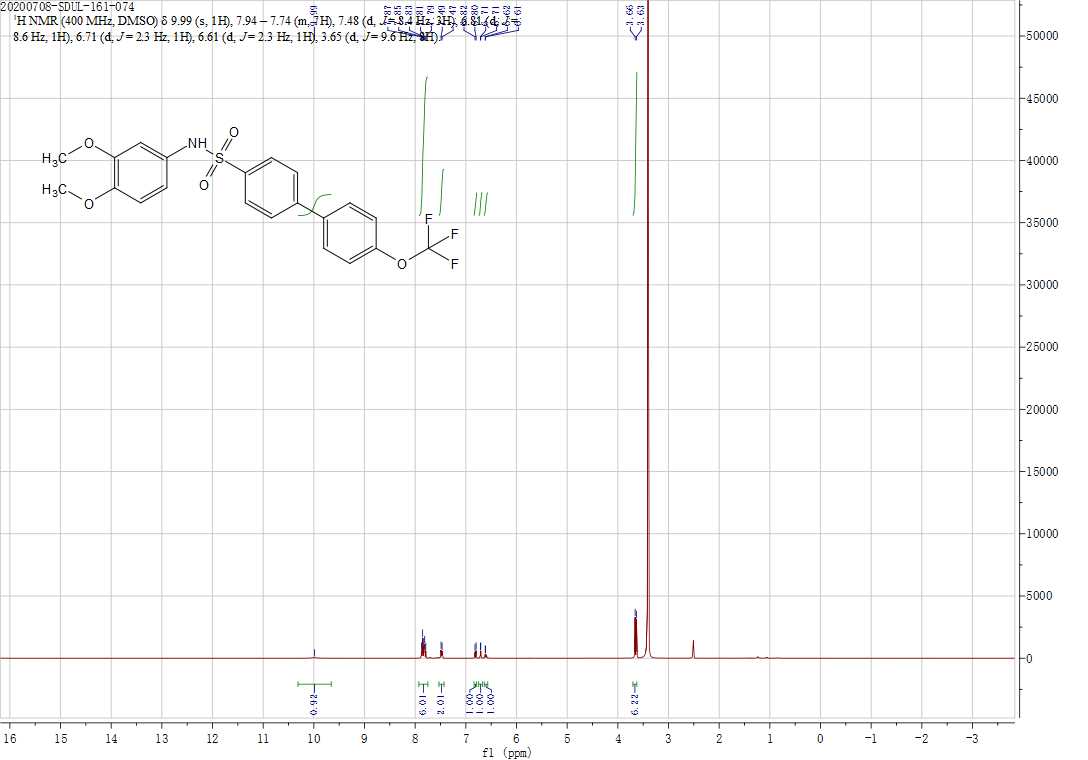
**

^1^H-NMR spectrum of compound **D1**.


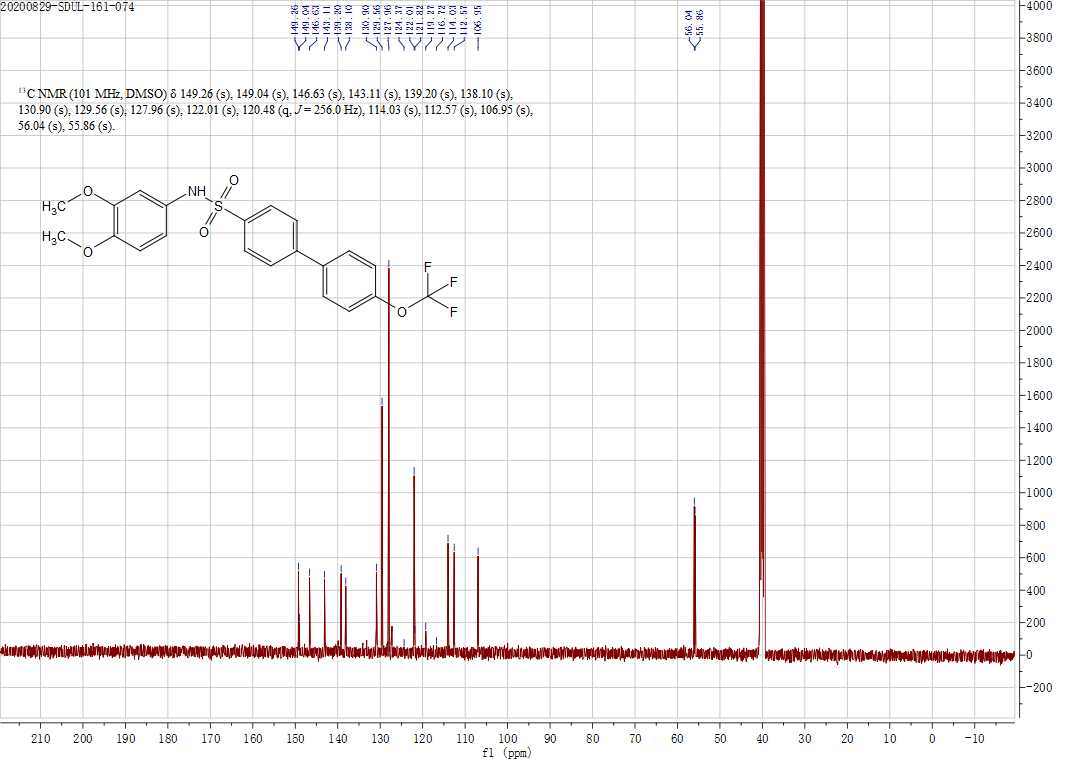


^13^C-NMR spectrum of compound **D1**.

HRMS spectrum of compound **D1.**

**
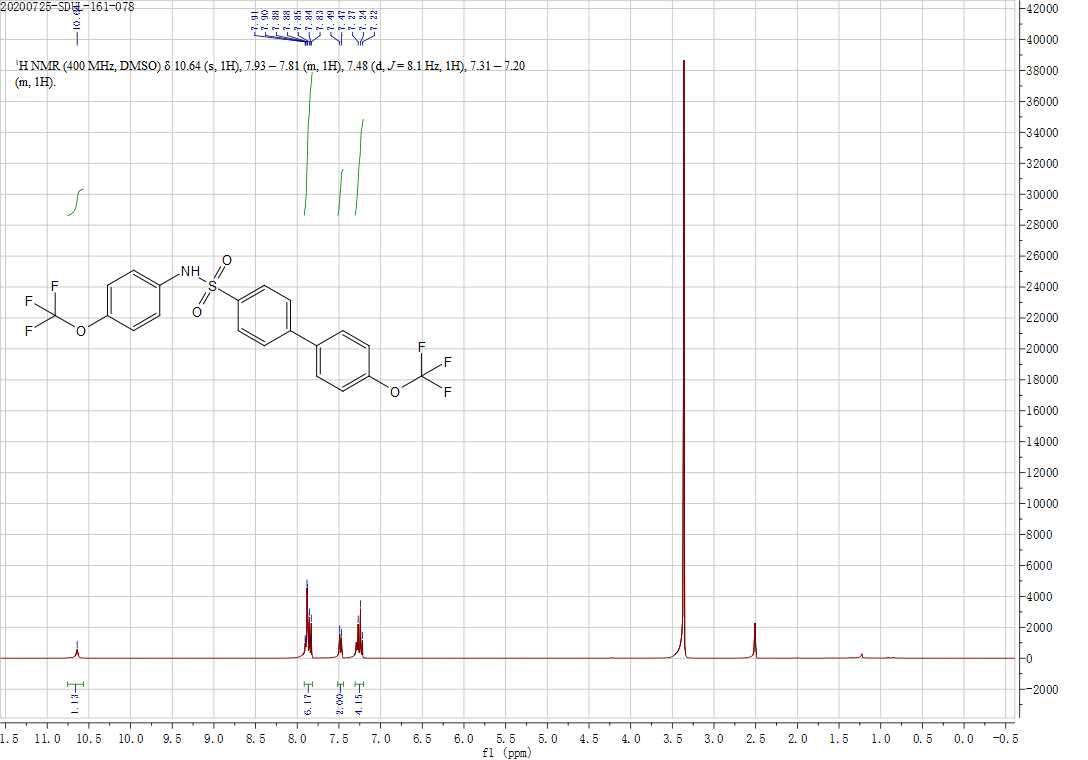
**

^1^H-NMR spectrum of compound **D2**.


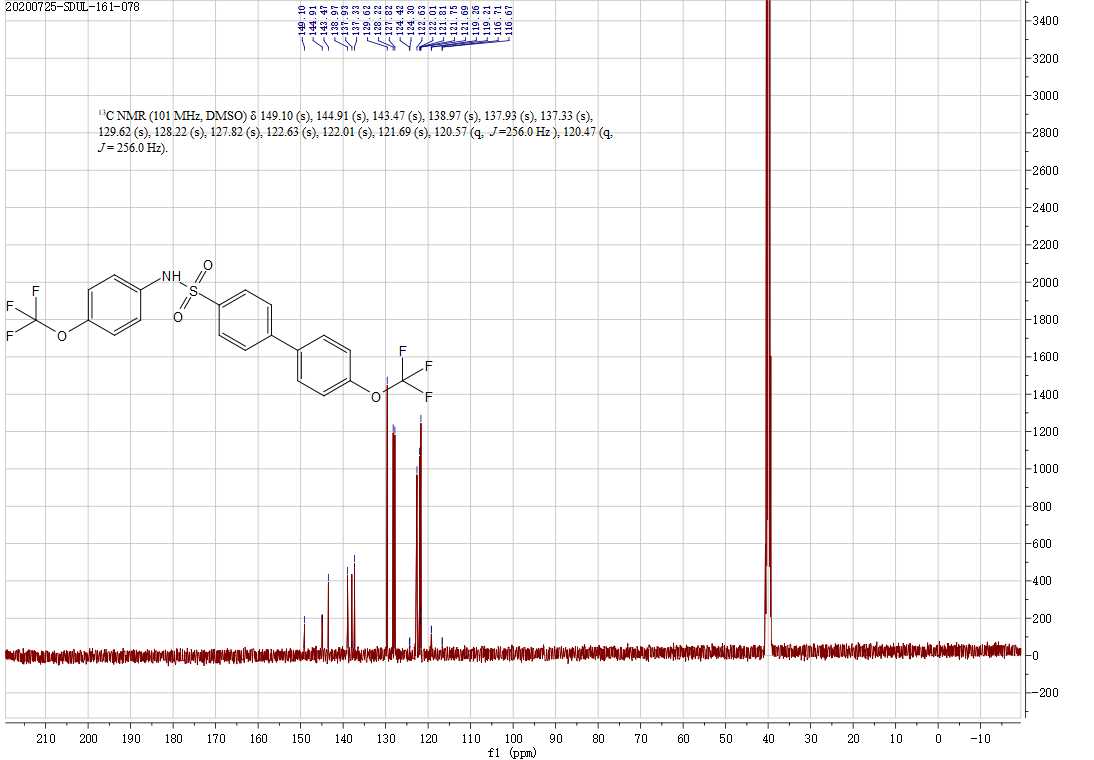


^13^C-NMR spectrum of compound **D2**.

HRMS spectrum of compound **D2.**

**
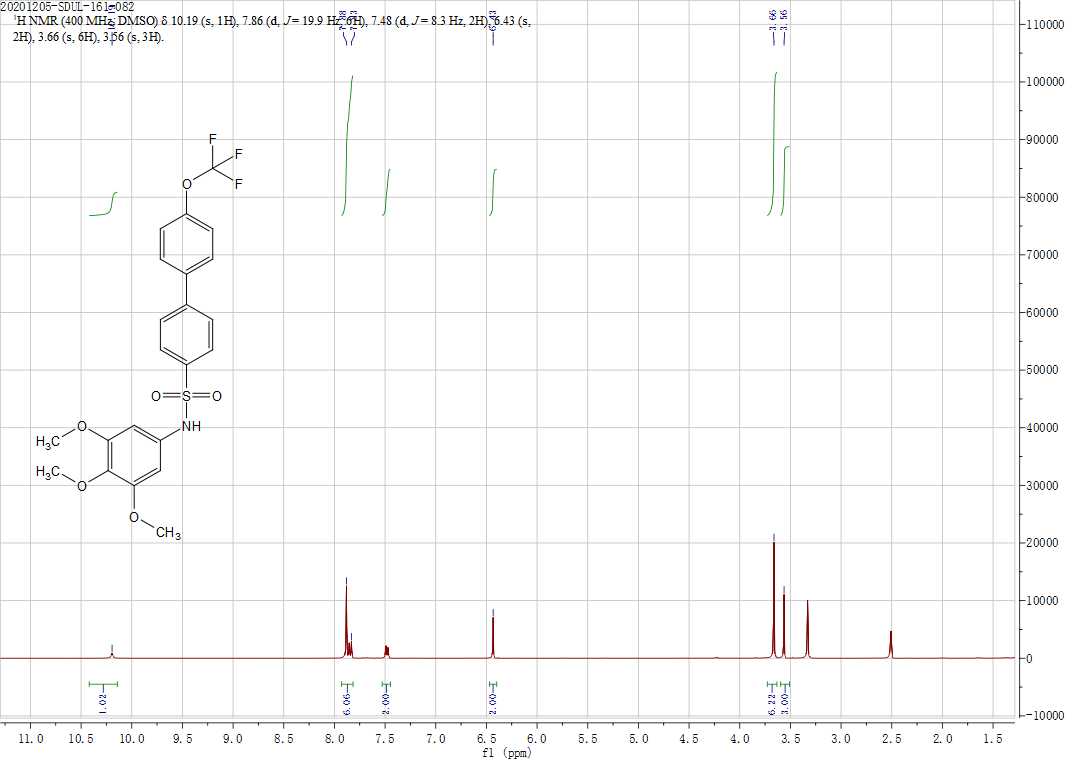
**

^1^H-NMR spectrum of compound **D3**.


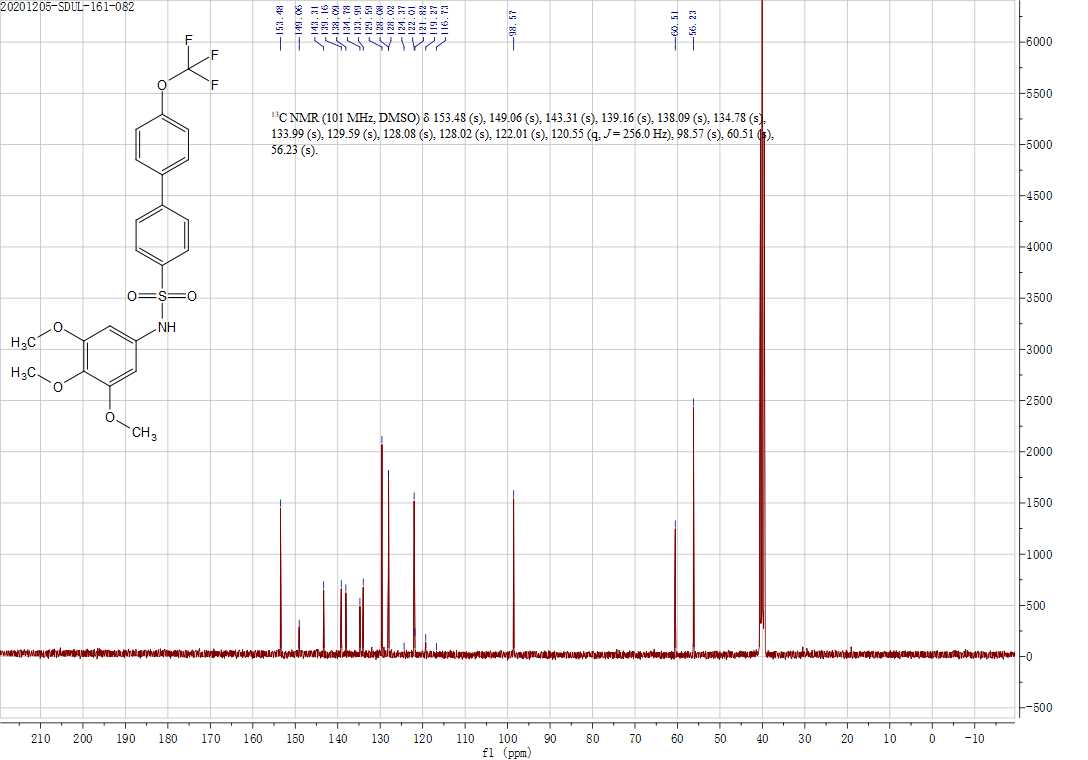


^13^C-NMR spectrum of compound **D3**.

HRMS spectrum of compound **D3.**

**
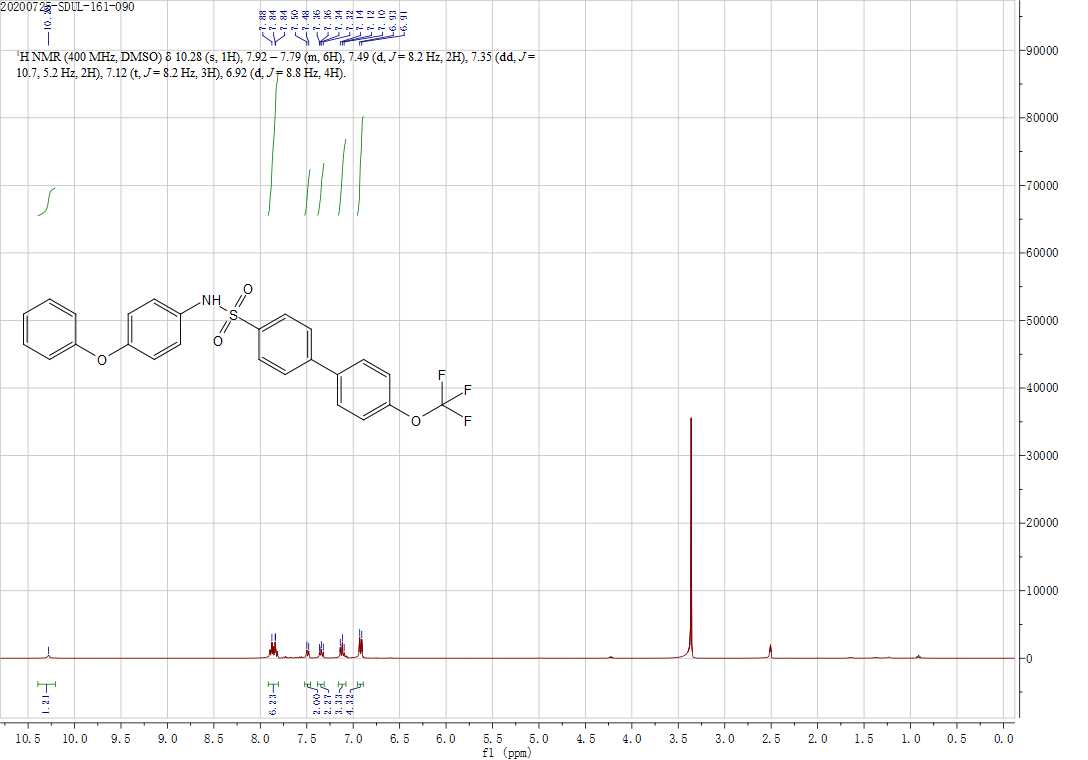
**

^1^H-NMR spectrum of compound **D4**.


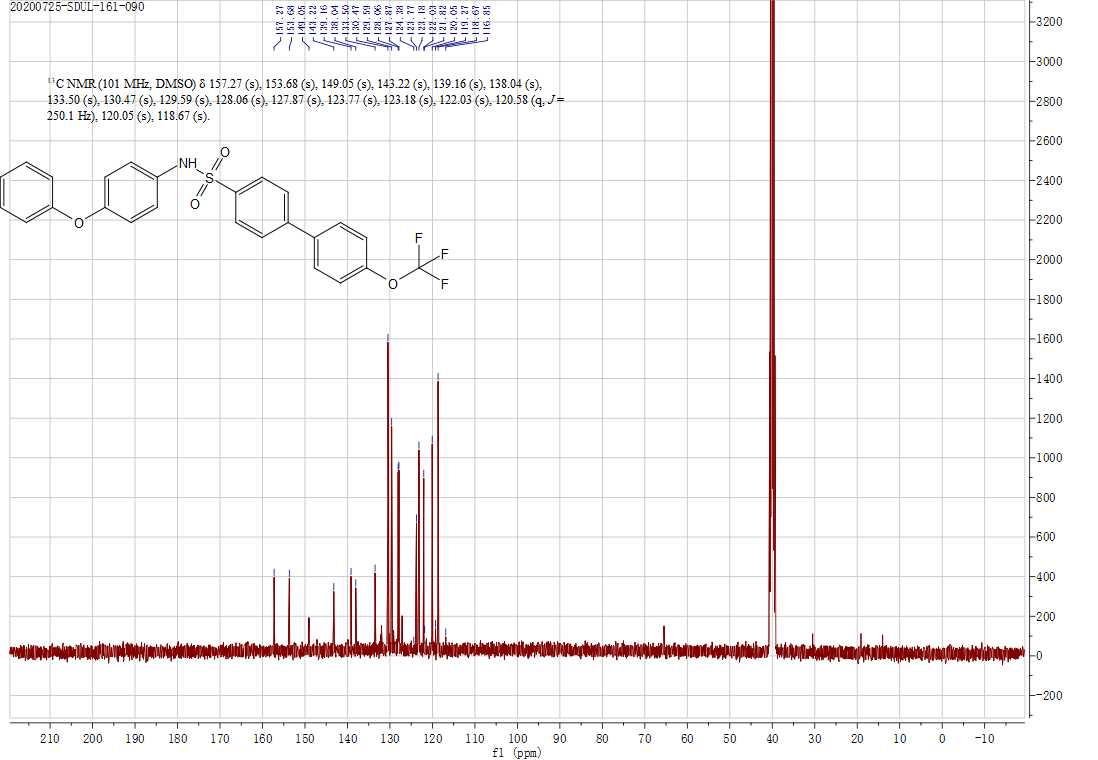


^13^C-NMR spectrum of compound **D4**.

HRMS spectrum of compound **D4.**

**
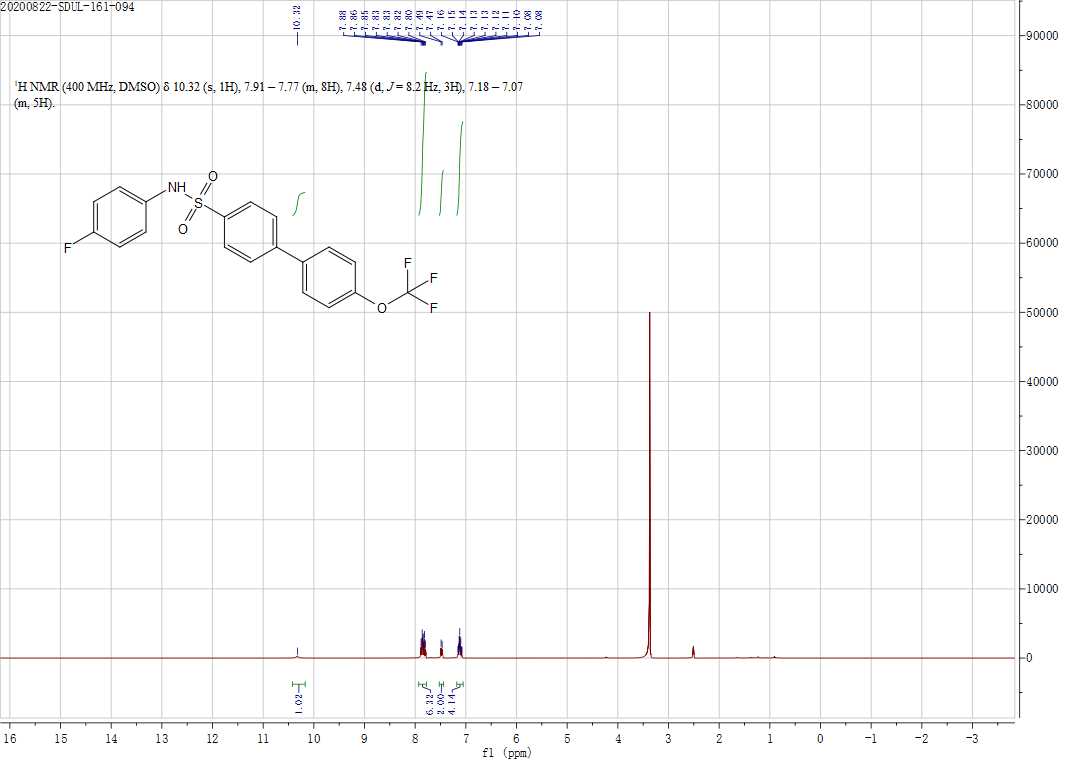
**

^1^H-NMR spectrum of compound **D5**.


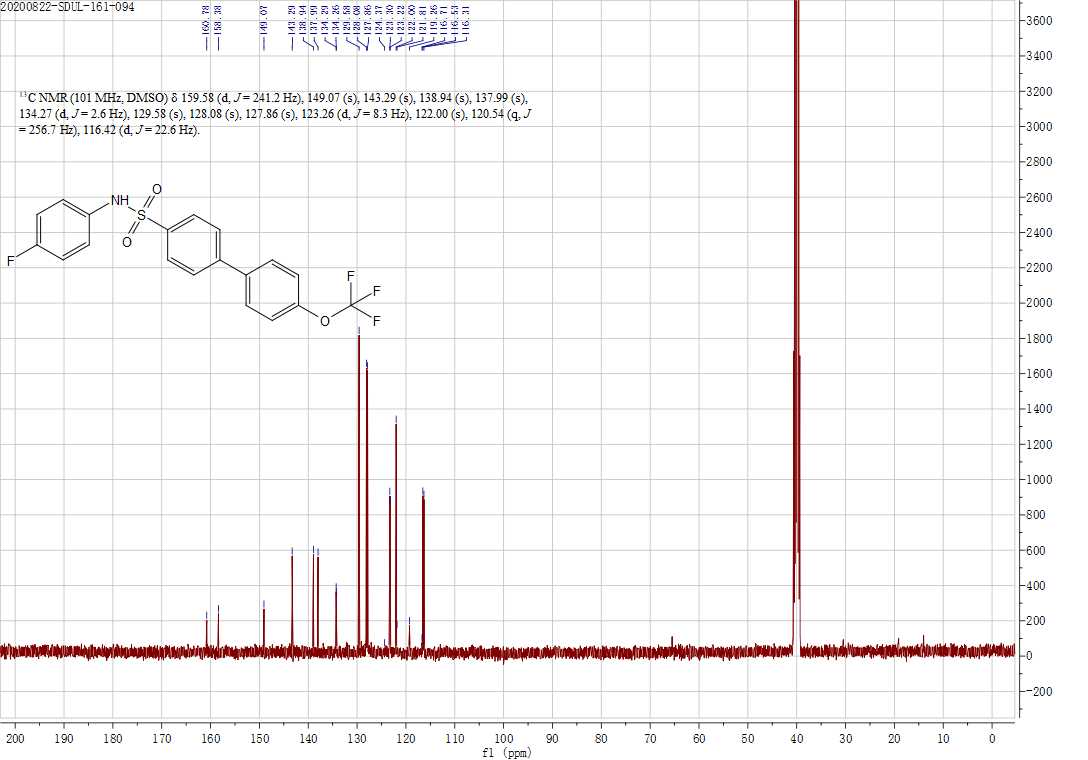


^13^C-NMR spectrum of compound **D5**.

HRMS spectrum of compound **D5.**

**
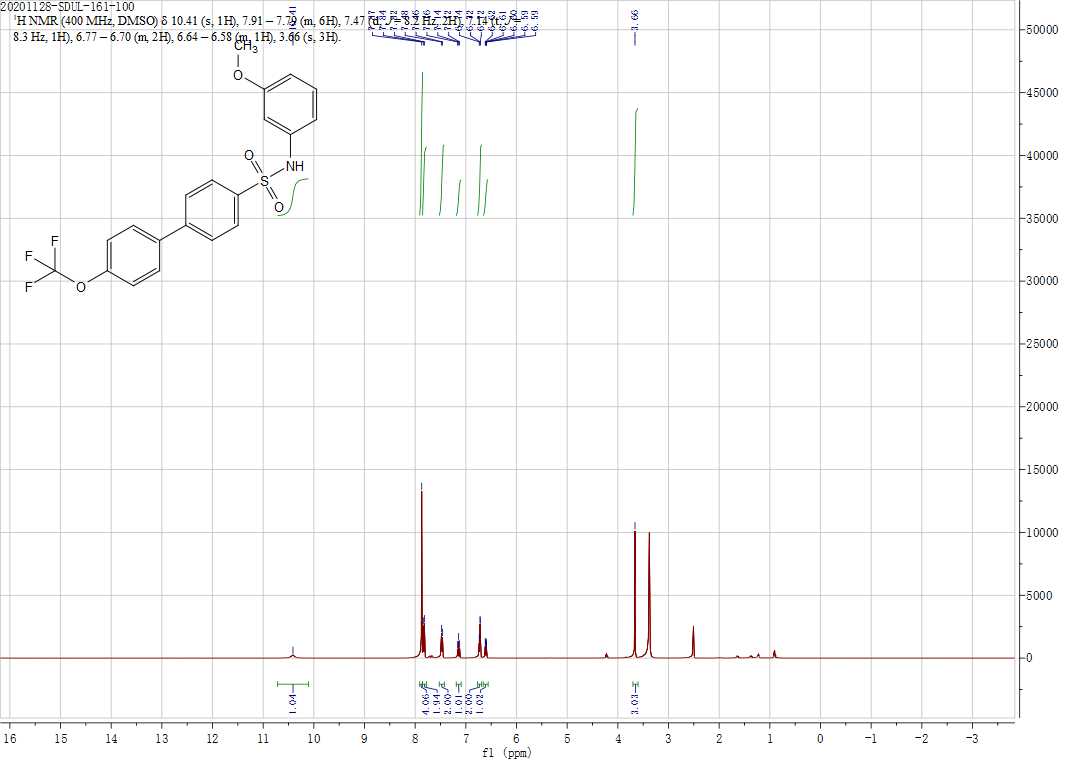
**

^1^H-NMR spectrum of compound **D6**.


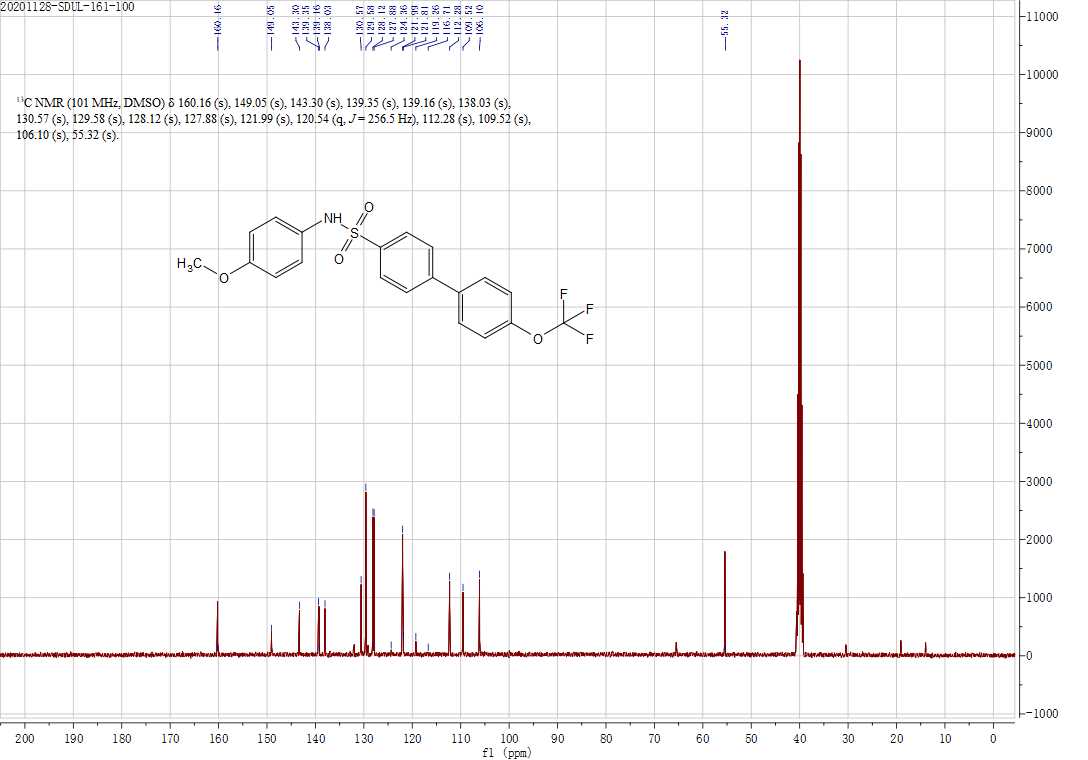


^13^C-NMR spectrum of compound **D6**.

HRMS spectrum of compound **D6.**

**
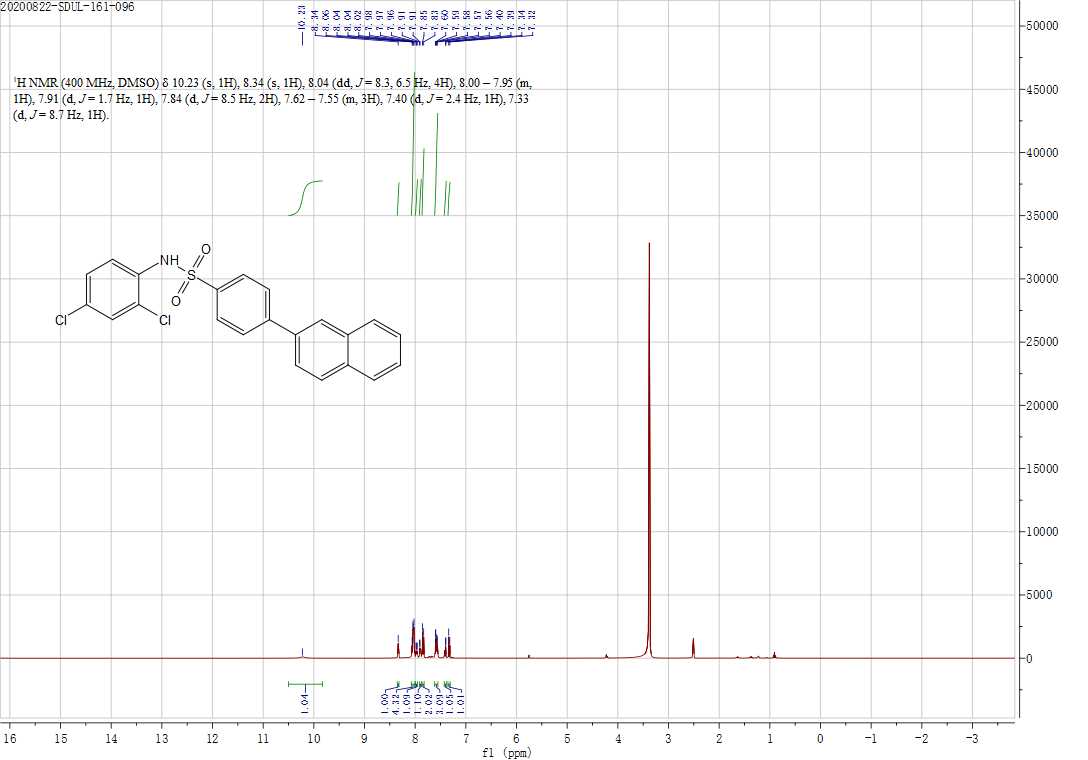
**

^1^H-NMR spectrum of compound **D7**.


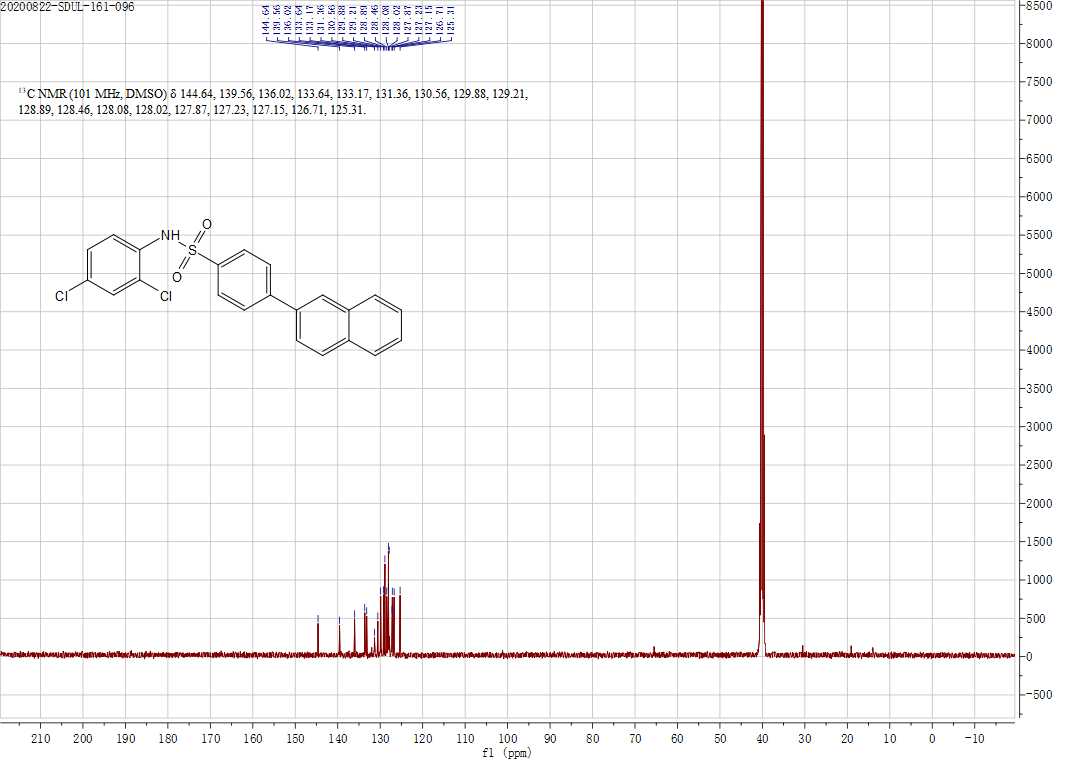


^13^C-NMR spectrum of compound **D7**.

HRMS spectrum of compound **D7.**

**
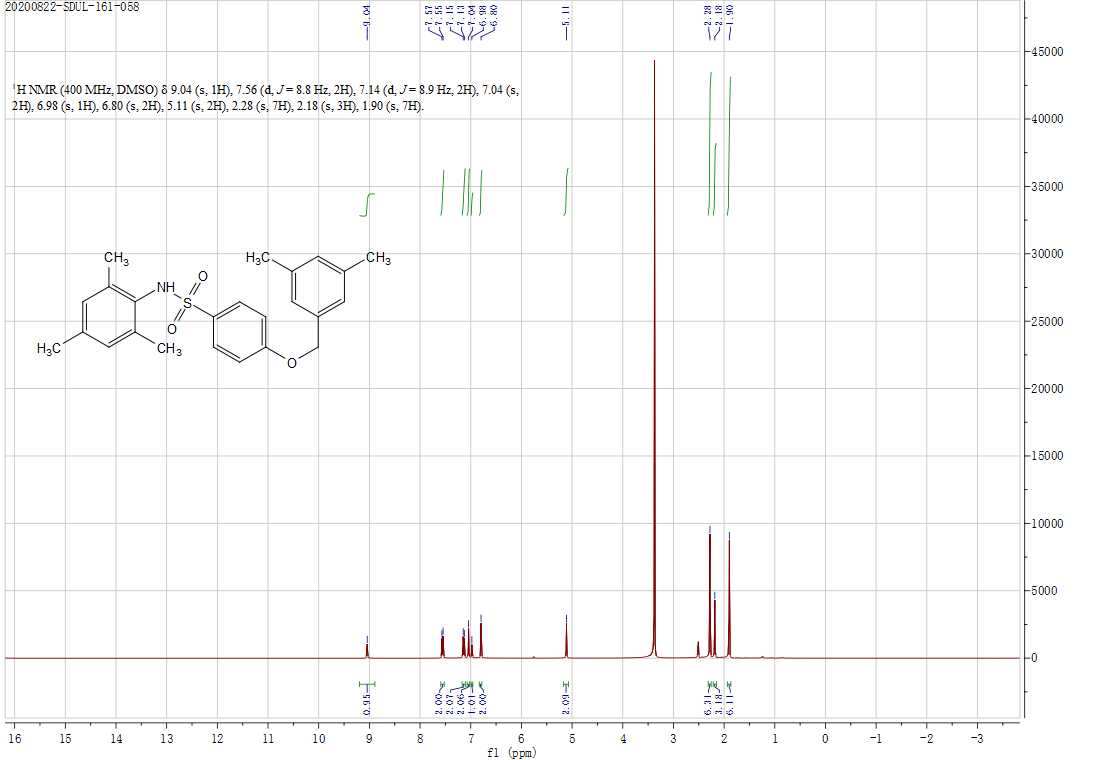
**

^1^H-NMR spectrum of compound **C1**.


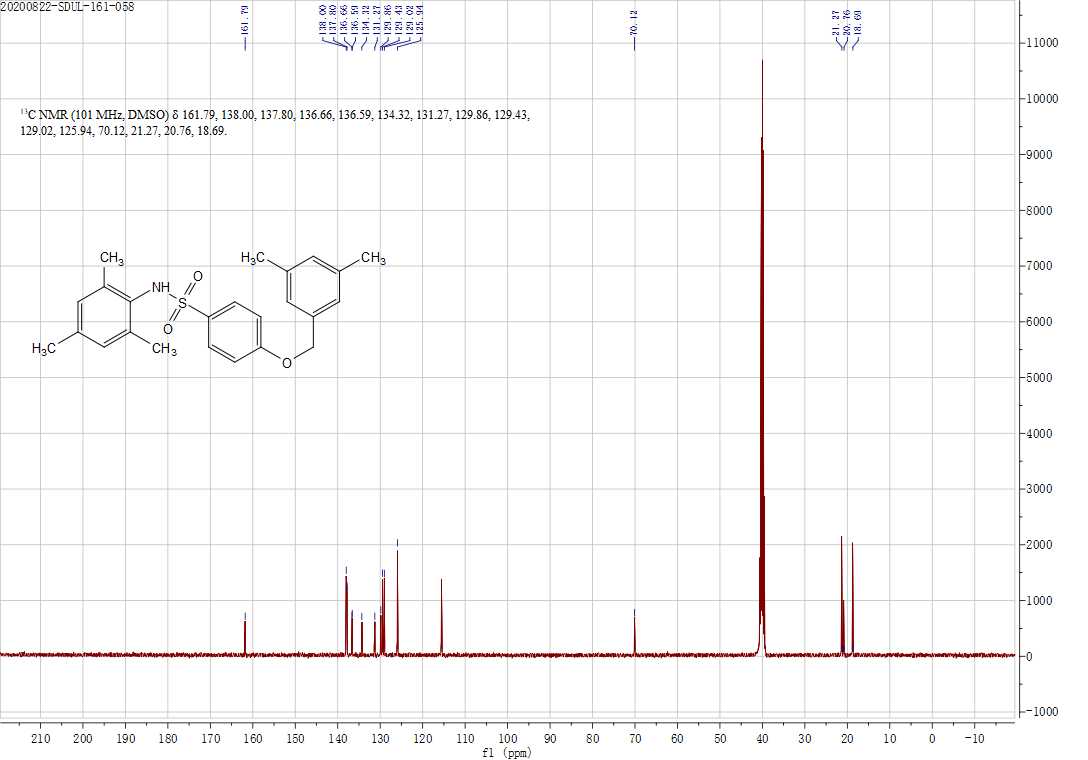


^13^C-NMR spectrum of compound **C1**.

HRMS spectrum of compound **C1.**

**
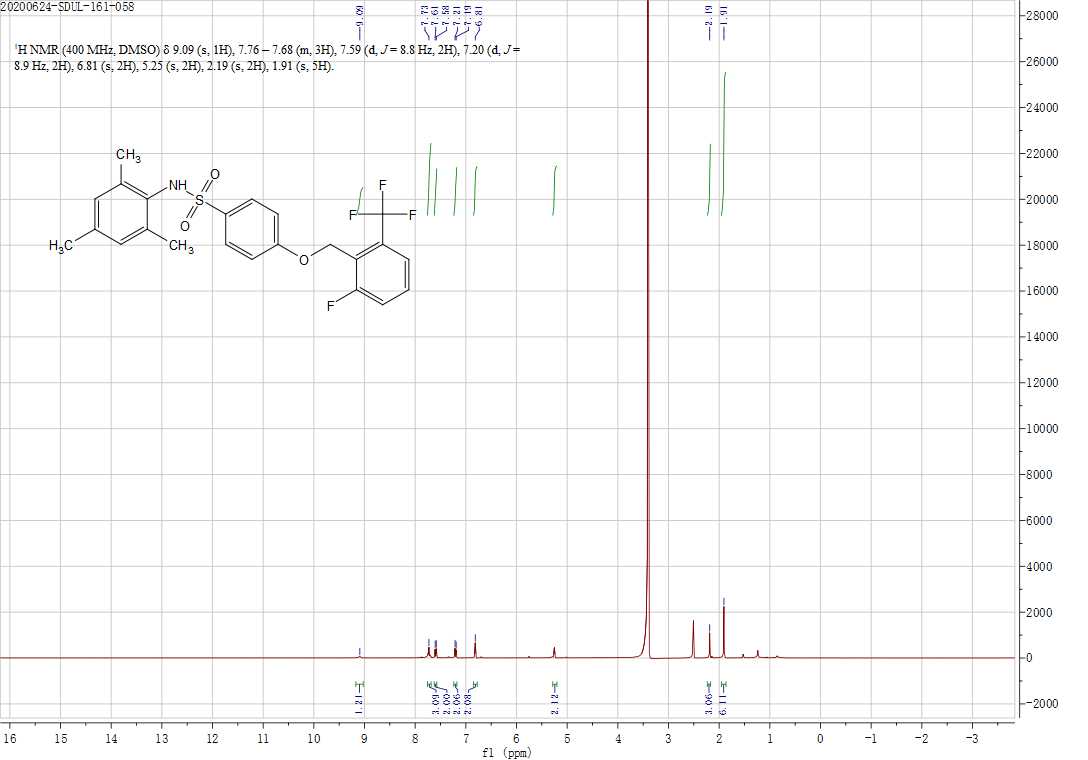
**

^1^H-NMR spectrum of compound **C2**.


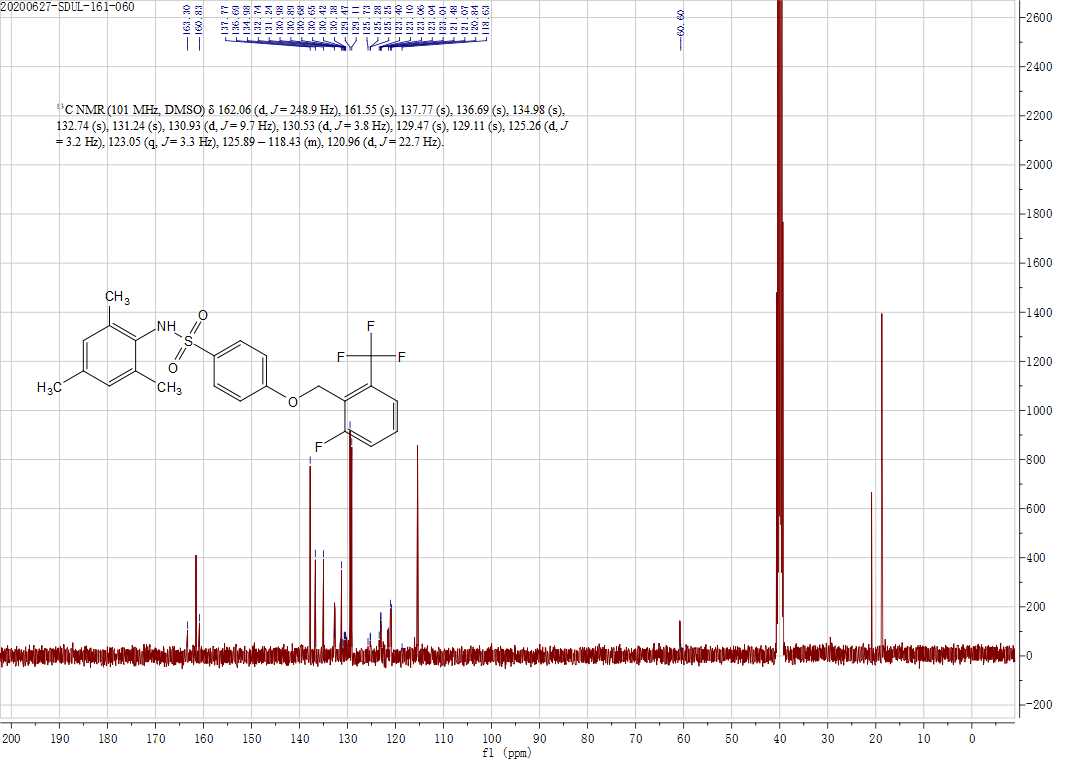


^13^C-NMR spectrum of compound **C2**.

HRMS spectrum of compound **C2.**

**
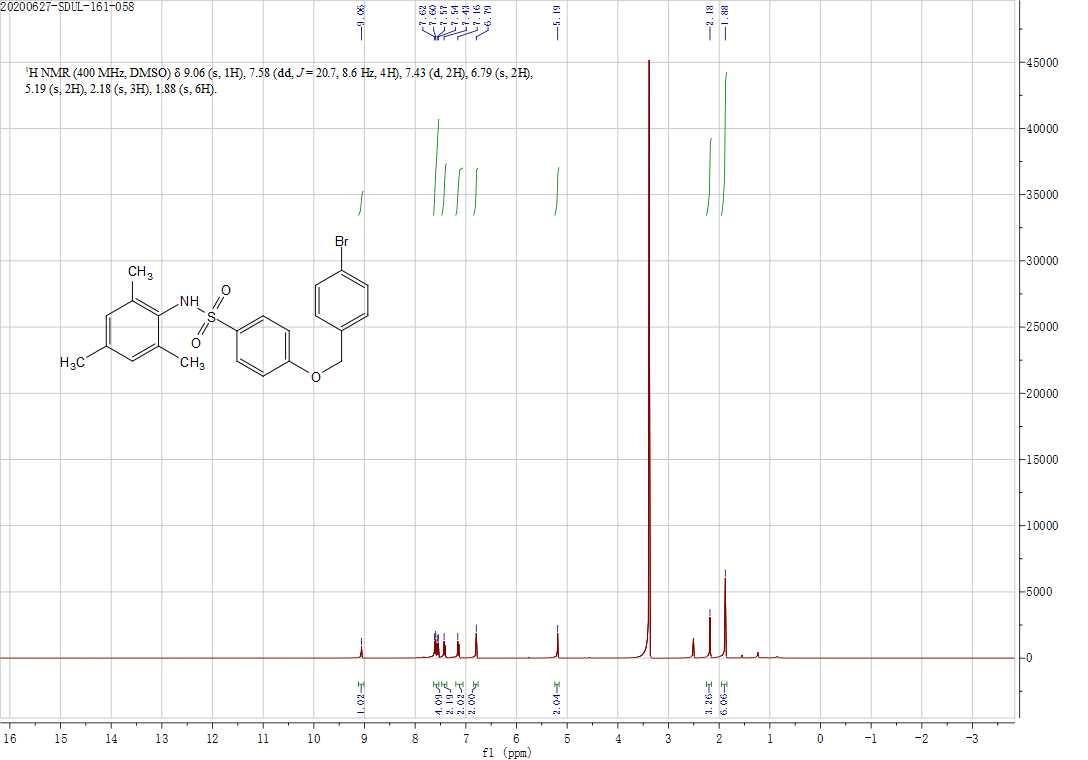
**

^1^H-NMR spectrum of compound **C3**.


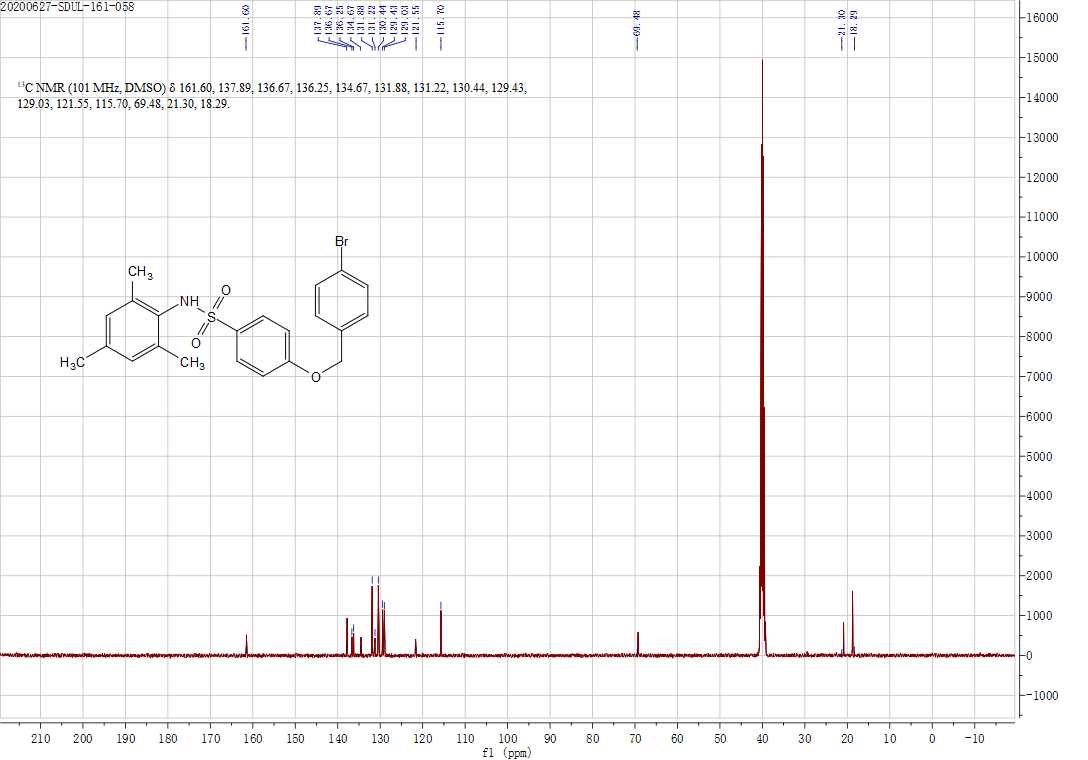


^13^C-NMR spectrum of compound **C3**.

HRMS spectrum of compound **C3.**

**HPLC data of final compounds**


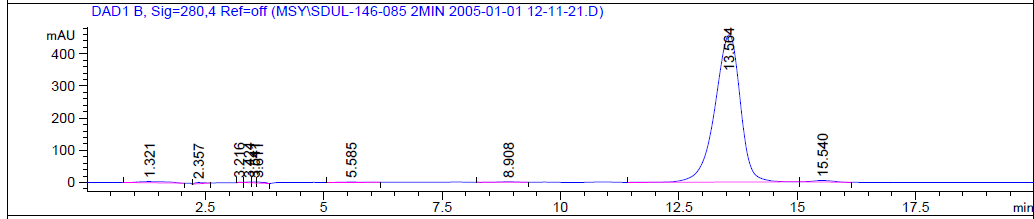


HPLC analysis of probe **N1**.


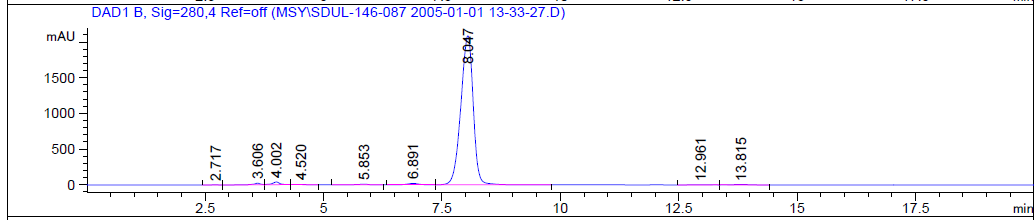


HPLC analysis of probe **N2**.


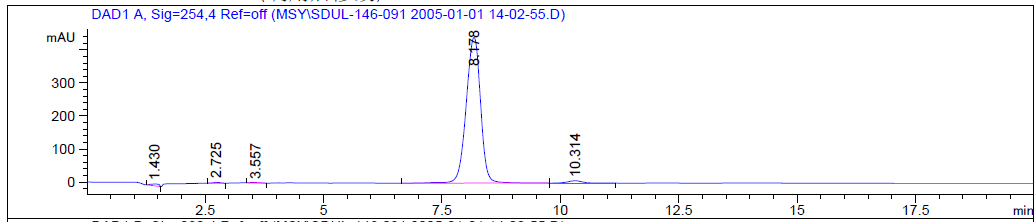


HPLC analysis of probe **N3**.


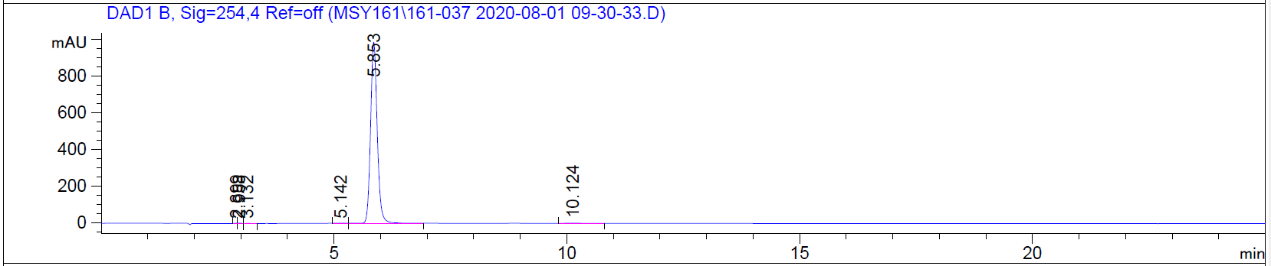


HPLC analysis of probe **A1**.


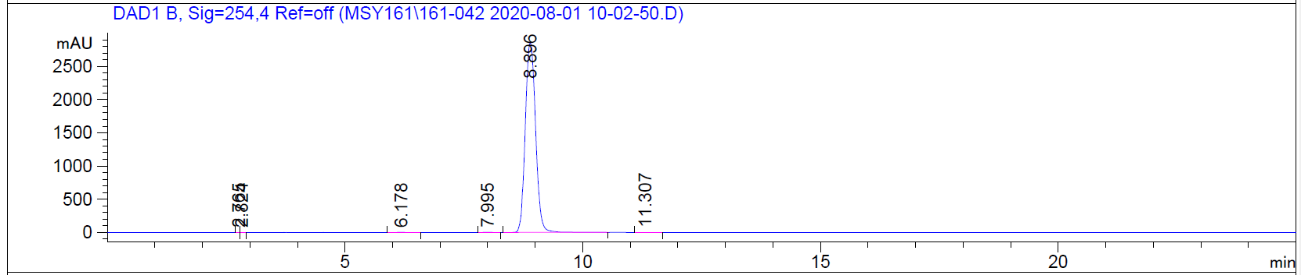


HPLC analysis of probe **B1**.


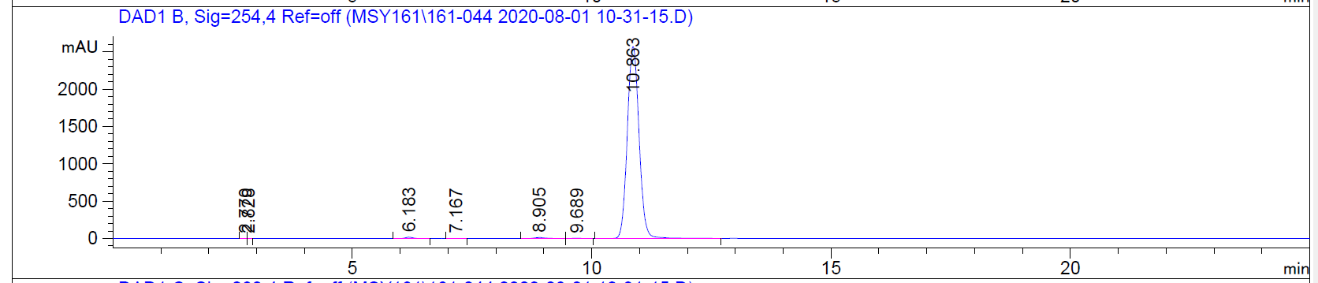


HPLC analysis of probe **B2**.


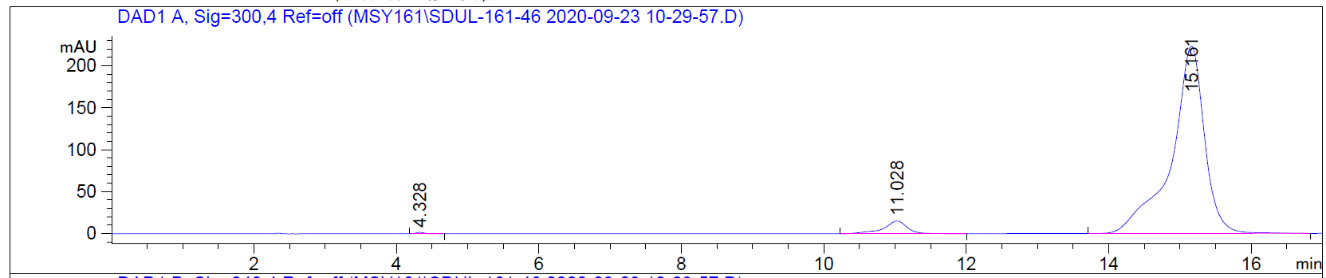


HPLC analysis of probe **B3**.


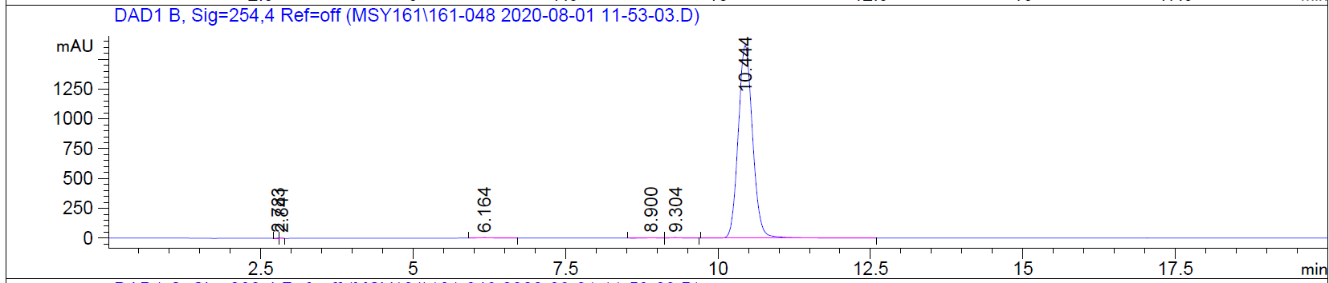


HPLC analysis of probe **B4**.


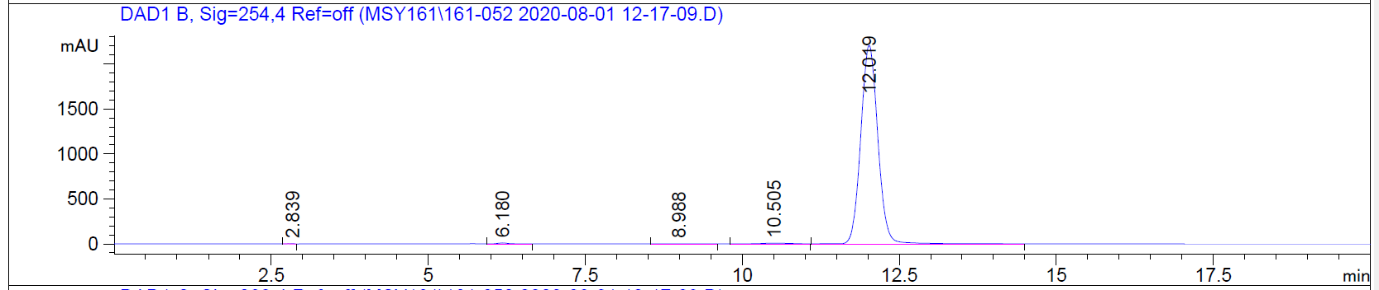


HPLC analysis of probe **B5**.


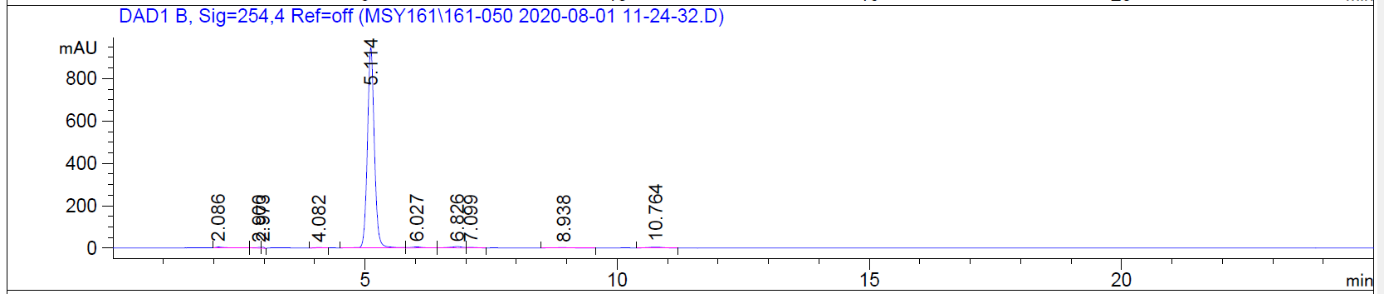


HPLC analysis of probe **B6**.


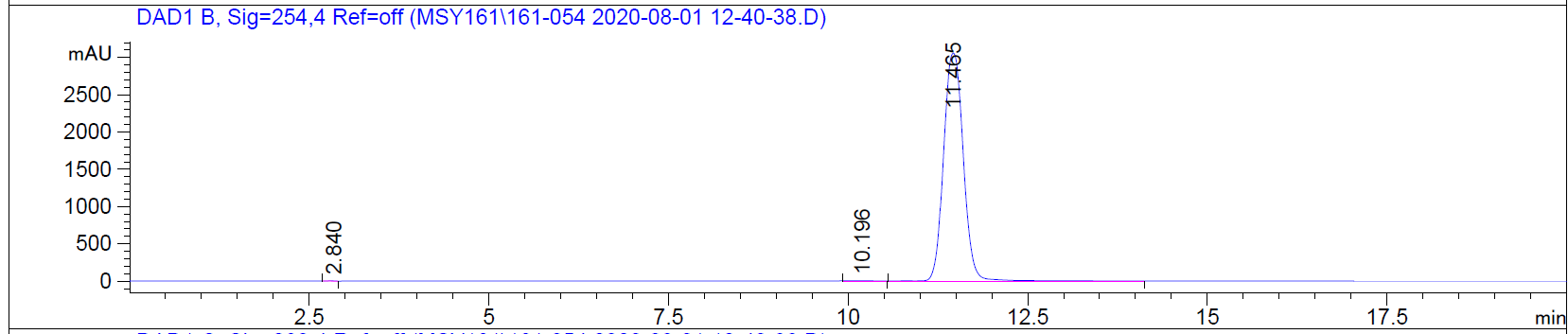


HPLC analysis of probe **B7**.


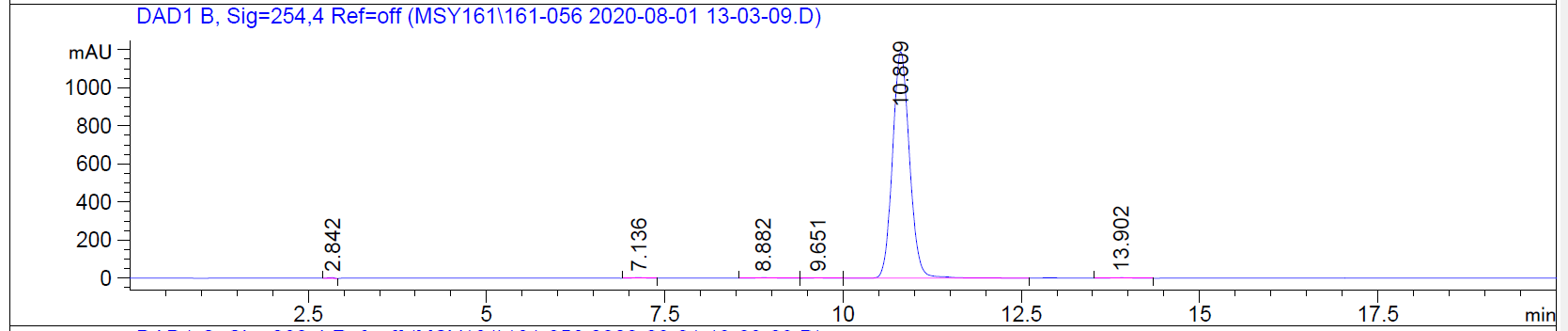


HPLC analysis of probe **B8**.


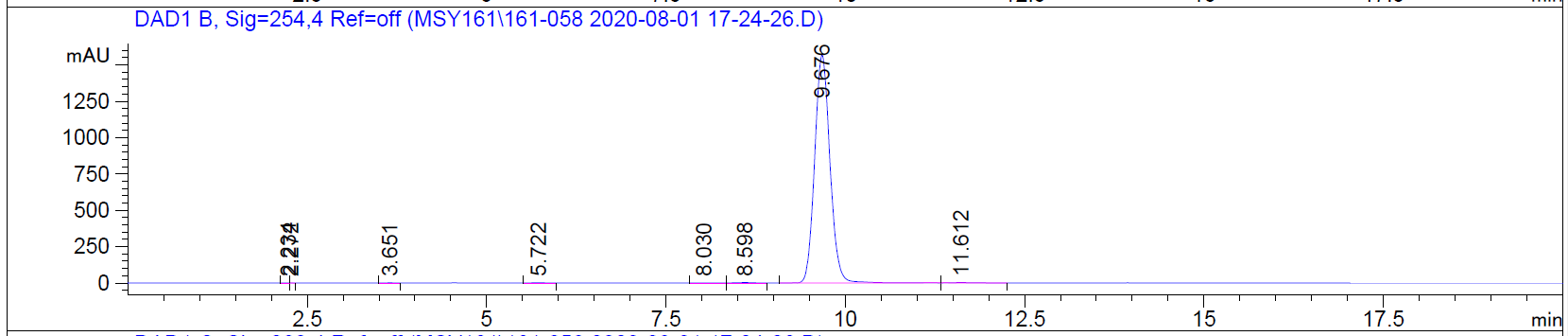


HPLC analysis of probe **C1**.


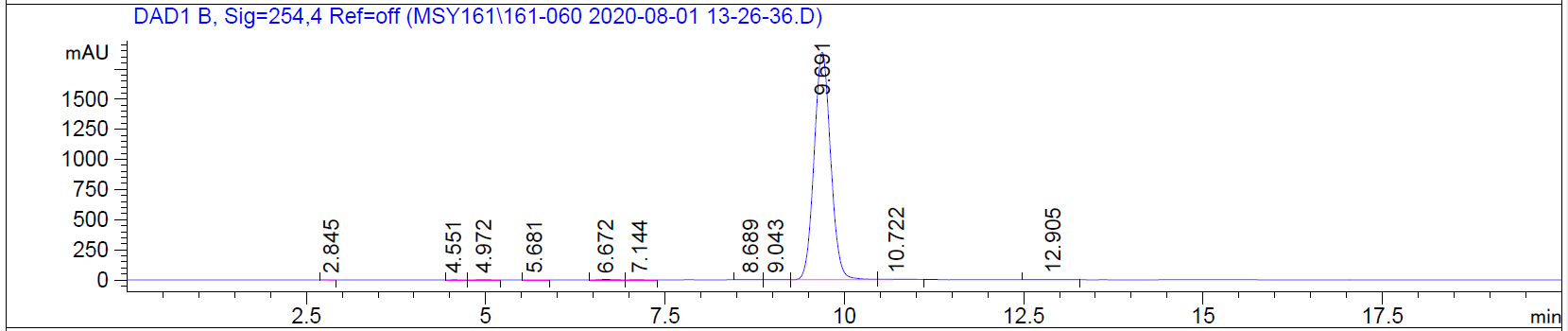


HPLC analysis of probe **C2**.


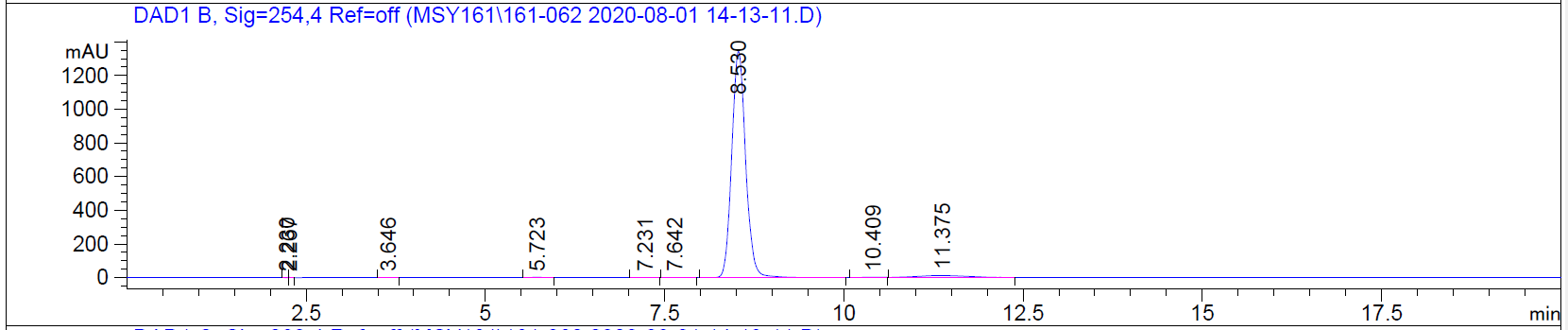


HPLC analysis of probe **C3**.


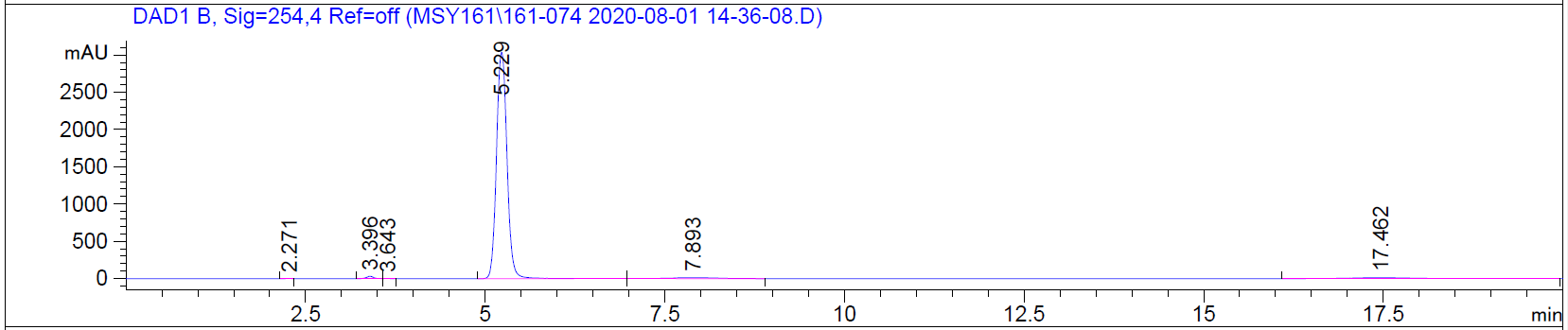


HPLC analysis of probe **D1**.


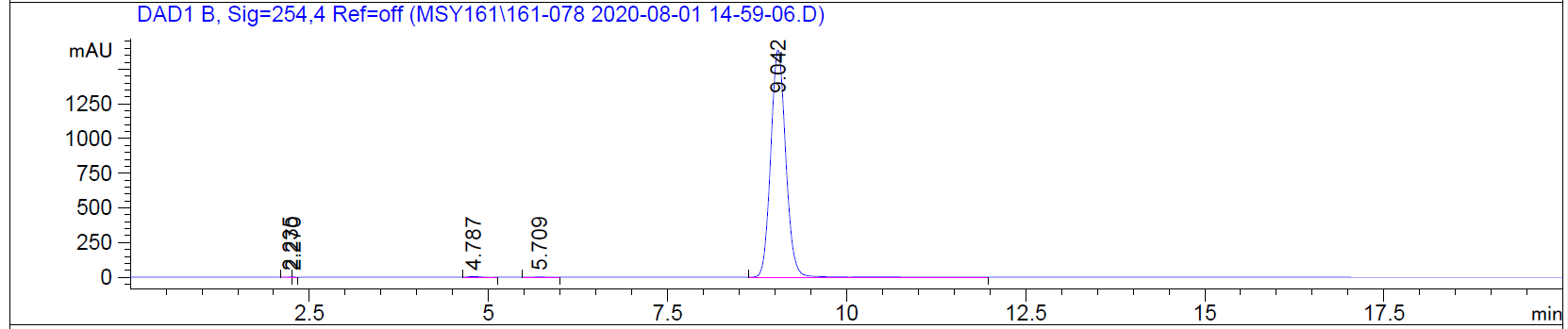


HPLC analysis of probe **D2**.


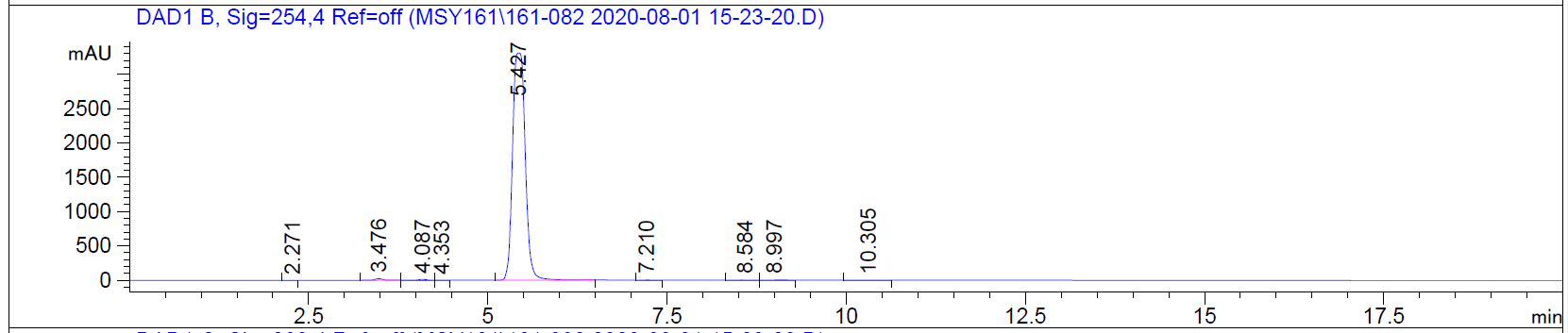


HPLC analysis of probe **D3**.


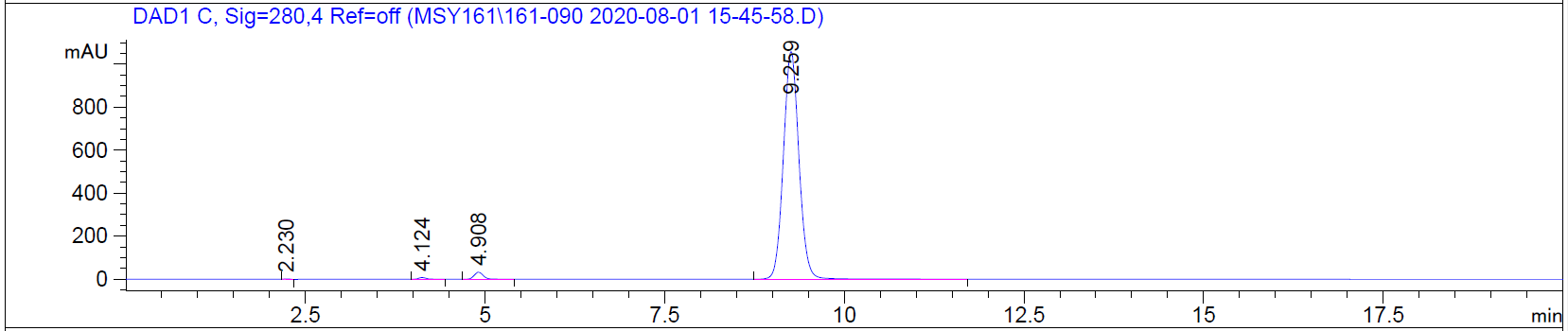


HPLC analysis of probe **D4**.


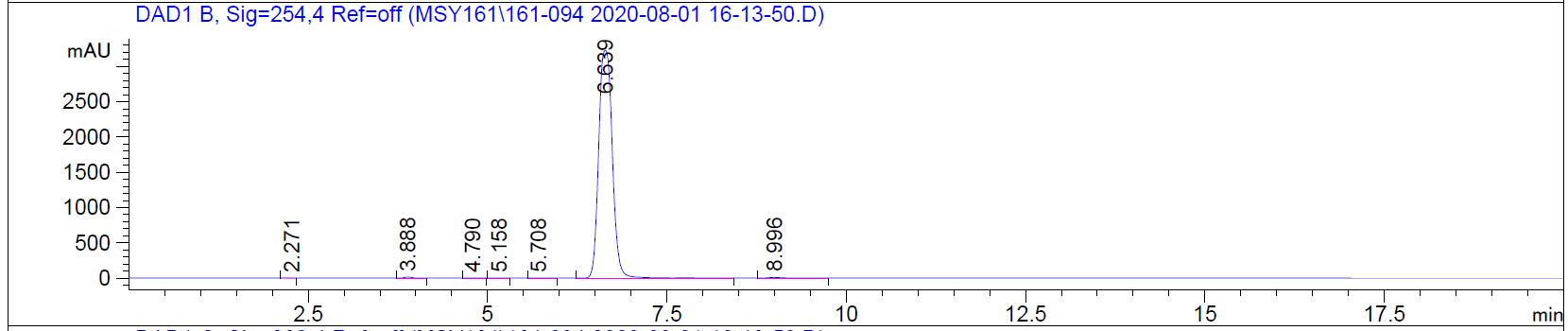


HPLC analysis of probe **D5**.


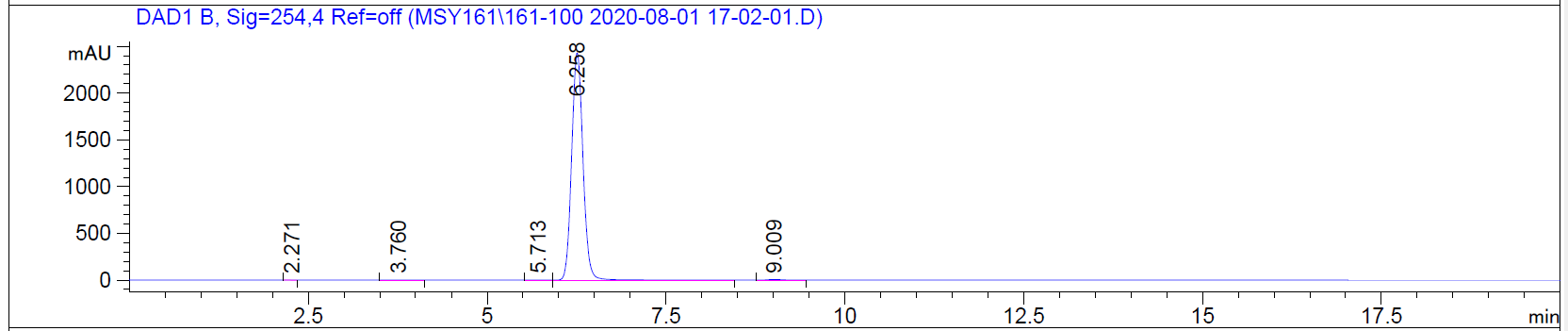


HPLC analysis of probe **D6**.


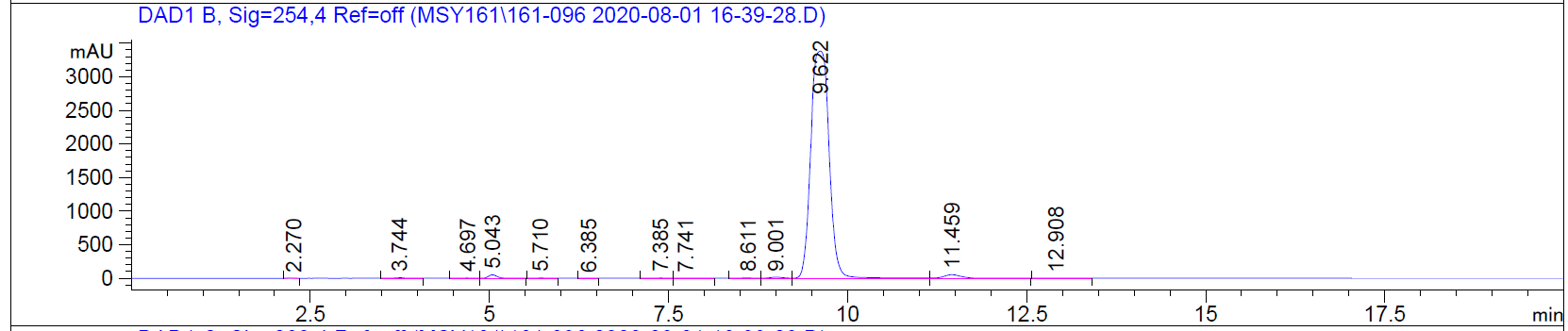


HPLC analysis of probe **D7**.
